# Shielding Probabilistically Checkable Proofs: Zero-Knowledge PCPs from Leakage Resilience

**DOI:** 10.3390/e24070970

**Published:** 2022-07-13

**Authors:** Mor Weiss

**Affiliations:** The Alexander Kofkin Faculty of Engineering, Bar-Ilan University, Ramat-Gan 5290002, Israel; mor.weiss@biu.ac.il

**Keywords:** Probabilistically Checkable Proofs, zero knowledge, leakage resilience

## Abstract

Probabilistically Checkable Proofs (PCPs) allows a randomized verifier, with oracle access to a purported proof, to probabilistically verify an input statement of the form “x∈L” by querying only a few proof bits. *Zero-Knowledge* PCPs (ZK-PCPs) enhance standard PCPs to additionally guarantee that the view of any (possibly malicious) verifier querying a bounded number of proof bits can be efficiently simulated up to a small statistical distance. The first ZK-PCP construction of Kilian, Petrank and Tardos (STOC 1997), and following constructions employing similar techniques, necessitate that the *honest* verifier makes several rounds of queries to the proof. This undesirable property, which is inherent to their technique, translates into increased round complexity in cryptographic applications of ZK-PCPs. We survey two recent ZK-PCP constructions—due to Ishai, Yang and Weiss (TCC 2016-A), and Hazay, Venkitasubramaniam and Weiss (ITC 2021)—in which the honest verifier makes a *single* round of queries to the proof. Both constructions use entirely different techniques compared to previous ZK-PCP constructions, by showing connections to the seemingly-unrelated notion of *leakage resilience*. These constructions are incomparable to previous ZK-PCP constructions: while on the one hand the honest verifier only makes a single round of queries to the proof, these ZK-PCPs either obtain a smaller (polynomial) ratio between the query complexity of the honest and malicious verifiers or obtain a weaker ZK guarantee in which the ZK simulator is not necessarily efficient.

## 1. Introduction

Proofs are a cornerstone of cryptography. They are an essential component of many cryptographic systems, guaranteeing correct execution in the presence of mutually-distrusting parties. Their applications range from mundane tasks such as proving one’s identity when signing into an email account, to general tasks such as proving honest behaviour in distributed systems, i.e., attesting that one had followed its prescribed algorithm.

Due to their centrality, in the past decades a long line of works have studied the notion of proofs, extending it far beyond the traditional notion to also allow for interaction between a *prover* P and a *verifier*V, as well as randomization. Various variants have arisen, depending on different properties of the system, such as the amount and type of communication allowed between the prover and verifier, their computational powers, and the type of randomness (for example, whether the verifier’s random coin tosses are private or public). These advances have reshaped theoretical computer science.

Our focus is on *Probabilistically Checkable Proofs (PCPs)* [1,2] augmented with a cryptographic *Zero-Knowledge (ZK)* property that is very useful when such proofs are used in cryptographic applications. These zero-knowledge PCPs combine aspects of (interactive) zero-knowledge proofs and PCPs, concepts which we now discuss.

**Probabilistically Checkable Proofs (PCPs).** A *Probabilistically Checkable Proof (PCP)* for a language L allows a randomized verifier V to verify a statement of the form “x∈L”, while querying only few bits of an oracle proof π that was generated by a prover P. (We note that the prover entity is not traditionally included as part of a PCP system. However, explicitly introducing this entity will be useful when PCPs are used for cryptographic applications, which necessitate efficient proof generation.) More specifically, V is given *x* as input, and P—who is usually required to be efficient—may be given additional information needed to efficiently generate the proof, e.g., a witness in the case of an NP language. More generally, for an NP-language L the PCP will usually be designed for some specific NP-relation $R\left(x,w\right)$ associated with L. In standard PCPs the prover is deterministic, whereas the verifier is randomized. (This is necessary since the verifier only queries few proof bits.) The verifier accepts true claims with probability 1, whereas the *soundness error*—namely the probability that a false claim is accepted—is small, regardless of the purported proof $\pi^{*}$ given to the verifier. The celebrated PCP theorem [1,2,3] asserts that any NP language has a PCP system with soundness error 1/2 in which the verifier V reads only a *constant* number of proof bits. Moreover, V is *non-adaptive*, namely its queries are determined solely by its randomness.

**Interactive and Zero-Knowledge Proofs.***Interactive Proofs (IPs)* [4] are a different kind of proof system, in which the efficient probabilistic verifier V interacts with a prover P, the goal of which is to convince V that x∈L for a joint input *x*. Such proofs are extremely powerful, compared to classical proofs: any PSPACE language has an interactive proof with a polynomial-time verifier [5], whereas classic proofs with a polynomial-time verifier only exist for NP languages. *Zero-Knowledge (ZK) Proofs* [4] are an important and useful generalization of IPs for NP, enhancing them to also guarantee privacy of the NP witness. These proofs carry no extra knowledge other than being convincing, in the sense that any information V can infer from its interaction with P, it could have (efficiently) computed given only the input *x*. This holds even in the presence of a *malicious* verifier $V^{*}$, namely a verifier who arbitrarily deviates from the protocol. It is important to note that while proofs are prominently used to protect *the verifier* (guaranteeing it would reject false claims), ZK is designed to protect the *prover*, guaranteeing that its private information (the NP witness, in this case) remains entirely hidden.

**Zero-Knowledge PCPs.***Zero-Knowledge PCPs (ZK-PCPs)* [6] are proofs systems that combine the advantages of PCPs and ZK proofs. These are PCPs with the additional guarantee that the view—encompassing all the knowledge which the verifier posses of the interaction—of any (possibly malicious) verifier $V^{*}$ who queries an *a-priori bounded* number of proof bits can be efficiently simulated given only the input, up to a small statistical distance. 

ZK-PCPs differ from traditional PCPs in several respects. First, whereas the PCP prover is traditionally deterministic, in ZK-PCPs the proof is randomized, and this is inherent to obtaining ZK. Second, ZK-PCPs are used to protect the *prover’s* private information (e.g., an NP witness) against *malicious verifiers*. More specifically, the system is associated with an a-priori query bound $q^{*}$, where ZK holds only against verifiers $V^{*}$ who query at most $q^{*}$ proof bits. We stress that this is the *only* restriction on $V^{*}$, and no further assumptions or limitations are made on its computational power or the manner in which it operates. It is important to note that bounding the query complexity is inherent in systems with efficient provers—an essential requirement for such systems to be useful for cryptographic applications. Indeed, the proof has polynomial length len, so any (efficient) verifier running in time len could read the *entire* proof, thus necessarily learning some information about the witness. Finally, we note that ZK against query-bounded verifiers is a stronger guarantee than *Honest-Verifier ZK (HVZK)*, namely ZK only against the *honest* verifier. This is because the honest verifier is always query bounded.

The models also differ in the main parameters of interest. Specifically, for PCPs these consist of the randomness and query complexities of the verifier (i.e., the number of coins it tosses and the number of queries it makes to the proof), which also determine the proof length. Moreover, it is standard to consider a constant soundness error—with a verifier that queries a constant number of proof bits—since this setting has strong connections to proving hardness of approximation results. We currently have PCPs, the length of which is a quasi-linear length in the witness length, with a constant soundness error (which can be amplified through repetition), and a non-adaptive honest verifier that queries a constant number of proof bits [3,7].

On the other hand, for ZK-PCPs we desire the soundness error to be negligible (in the input length, or some security parameter) as is standard in cryptographic systems, and thus the query complexity is necessarily polylogarithmic. The query bound $q^{*}$ on malicious verifiers is another important parameter of the system, and would ideally be much larger than the query complexity of the honest verifier, e.g., polynomial in the input length. Thus, the *query gap* between the query complexity needed to verify the proof, and the number of queries a malicious verifier can make without violating ZK, would be exponential. Finally, we would like the *honest* verifier to be non-adaptive, namely to make a single round of queries to the proof. This should be contrasted with adaptive verifiers the queries of which might depend on the oracle answers to previous queries, and who therefore necessarily make several rounds of queries to the proof. As we will shortly explain, whereas the verifier in traditional PCPs is non-adaptive, the honest verifier in certain ZK-PCP constructions is (inherently) adaptive. We note that similar to traditional PCPs, the proof length is also of interest.

The focus on these parameters in ZK-PCP constructions stems from their effect on the properties and parameters of cryptographic systems using ZK-PCPs. Specifically, the query complexity and adaptivity of the honest verifier translates into communication and round complexities; the query-bound on malicious verifiers corresponds to the privacy guarantee of the resultant system; and in distributed proof systems (as in, e.g., [8]) the proof length and query bound translate into the total number of parties and the number of corrupted parties, respectively.

**ZK-PCP Constructions.** The first ZK-PCP for NP, due to Kilian, Petrank and Tardos [6], obtained a negligible soundness error with an honest verifier that queries $q=\mathrm{polylog}\left(\left|x\right|\right)$ proof bits, and ZK against verifiers making $q^{*}=p\left(\left|x\right|\right)$ queries to the proof for a *fixed* polynomial *p* that is much smaller than the proof length, but is much larger than *q*. (Earlier constructions, e.g., [9], obtained only limited ZK guarantees such as HVZK). Later works [10,11] simplified the system, making it more modular, and also generalized it to other proof models (specifically, PCPs of proximity with zero knowledge [11]). While obtaining a desirable exponential query gap, the honest verifier in all these constructions is *adaptive*, namely it makes several rounds of queries to the proof, a severe limitation when the system is used in cryptographic applications. Unfortunately, the honest verifier’s adaptivity is inherent to these constructions, as we now explain. (We note that another line of works obtain non-adaptive verification by “pushing” adaptivity to the prover side; see Section 1.3. Having adaptive proof generation has similar disadvantages to having adaptive verification.) 

These ZK-PCP constructions follow the blueprint of [6], who show a 2-step compiler from a standard non-ZK PCP into a ZK-PCP. In the first step, the PCP is transformed into a PCP with HVZK—a weak ZK guarantee that holds only for the *honest verifier*. In the second step, HVZK is “boosted” into full-fledged ZK, against any (possibly malicious) query-bounded verifier $V^{*}$. This is obtained by forcing—through modifications made to the proof—$V^{*}$’s queries to be distributed similarly to the queries of the honest verifier V. This restriction on the verifier’s queries is imposed by combining an information–theoretic analogue of a standard cryptographic commitment, called a “locking scheme” [6], with a modified version of the PCP obtained in the first step. The proof generated in this second step requires adaptive verification, due to the structure of the modified version of the proof, as well as the use of locking schemes. We note that this description of $V^{*}$’s queries as being distributed “similarly” to the queries of V is in fact a gross over-simplification—for example, $V^{*}$ can query many more proof bits compared to V. Somewhat more accurately, these works effectively force $V^{*}$’s queries to be distributed similarly to a “repeated” version of V (obtained by emulating V multiple times with independent random coins). We refer the interested reader to [11,12] for more details. We stress that these works do *not* make any *assumptions* on the query pattern of $V^{*}$, but rather by appropriately constructing the proof they guarantee that *any* query pattern will be “harmless” in the sense that it reveals no information about the NP witness.

The second step can be very roughly (and somewhat inaccurately) illustrated through the following example: Alice is trying to locate a particular CD cd in Bob’s CD collection, which Bob has mixed in the following way: (1) the CDs were taken out of their cases and randomly placed back into the cases (where Bob knows which case contains which CD), and then (2) each CD case was locked in a transparent box. To get cd, Alice must first ask Bob in which case he put cd. Once this is known, she can locate the box in which this CD case is, but still needs to ask Bob for the key which unlocks the box. Since Alice cannot predict in which case cd is, she must first wait for Bob’s answer to her first query, before making her second query. (The PCP-version of this example will have Bob somehow write down the list of pairs (CD, CD case), as well as the keys, in the oracle proof.) In the ZK-PCP, step (1) of randomly mixing the CDs corresponds to the modifications performed to the proof to guarantee that $V^{*}$’s queries are “harmless”, specifically that $V^{*}$ cannot “cherry pick” specific locations in the HVZK PCP generated in the first step of the compiler. Moreover, step (2) of locking CDs in boxes corresponds to locking proof symbols in locking schemes, and even the process of unlocking (given the key) in itself is adaptive.

The cost of ZK in these ZK-PCPs is high: it incurs adaptive verification, even if the underlying PCP can be verified non-adaptively, which is indeed the case for traditional (non-ZK) PCPs. This naturally gives rise to the following research goal:

$$\begin{array}{c} Design\;ZK-PCPs\;with\;a\;non-adaptive\;honest\;verifier,\;and\;ZK\;against\;malicious\\ query-bounded\;verifiers. \end{array}$$

Obtaining non-adaptive verification is motivated by the goal of matching the parameters of non-ZK PCPs, as well as by cryptographic applications of ZK-PCPs, in which adaptive verification translates into increased complexity of the resultant system.

The question of designing non-adaptive ZK-PCPs had remained open for nearly 20 years, until Ishai, Weiss and Yang [8] gave the first construction of a non-adaptive ZK-PCP, which was followed by the non-adaptive ZK-PCP of Hazay, Venkitasubramaniam and Weiss [13]. The focus of this survey is on describing and comparing these constructions.

### 1.1. Non-Adaptive ZK-PCPs

We survey two recent works [8,13] that construct ZK-PCPs for NP with a *non-adaptive honest verifier*, obtained through a novel connection to the seemingly unrelated field of *leakage-resilient cryptography*. These constructions differ drastically from the ZK-PCP constructions described above. This is not surprising, since adaptive verification is inherent to the latter, so obtaining non-adaptive verification necessitates an entirely new approach.

**Malicious Verifiers Through the Leakage-Resilience Lens.** Recall that the ZK-PCPs of [6,10,11] are obtained from a PCP with weak ZK guarantees (specifically, HVZK) by effectively *restricting the malicious verifier*, i.e., forcing its queries to be distributed similarly to the queries of (multiple independent copies of) the honest verifier. The ZK-PCPs of [8,13] take a different approach: instead of forcing a certain structure on $V^{*}$’s queries, they classify the *type of information* which an arbitrary query-bounded $V^{*}$ obtains by querying the proof, and modify the proof to guarantee this type of information reveals nothing about the underlying NP witness. Their insight is that the partial information obtained by deviating from the honest verifier’s query pattern constitutes *leakage* on the proof and consequently, on the underlying witness. (In a broader cryptographic context, *leakage* roughly refers to the information an adversary obtains by deviating from the assumed adversarial model for which the system was designed. For example, so-called “side channel” attacks—such as measuring the power consumption of an object—exploit adversarial capabilities which were not taken into account when designing the system (adversarial and attack models traditionally disregard such information, that is obtained from the *physical implementation*, and is not part of the more abstract model description). Similarly, a malicious verifier querying a PCP is capable of querying more—and different sets of—proof bits compared to the honest verifier for which the system was designed.) Accordingly, they employ tools from the leakage-resilience literature to protect the witness and proof.

The works of [8,13] differ in the method they use to protect against leakage. Hazay et al. [13] chose to protect *the proof* itself, whereas Ishai et al. [8] protect the *process of proof generation* from the witness. Put differently, the latter protect *computation* against leakage, for which they employ leakage-resilient *circuits*, whereas the former protect *information* (the proof, once it has been generated), by using an appropriate leakage-resilient *encoding*. Consequently, these works differ in their requirements from the underlying PCP, and in the parameters and properties of the resultant ZK-PCP. We now elaborate on these differences (see also Table 1).

#### 1.1.1. The ZK-PCPs of Hazay et al.

Hazay et al. [13] construct a non-adaptive ZK-PCP in which the ratio between the bound $q^{*}$ on the query complexity of a malicious verifier, and the query complexity *q* of the honest verifier, is polynomial.

The leakage-resilient primitive they employ is a *Leakage-Resilient Encoding (LRE)*. Roughly, an LRE consists of a randomized efficient encoding procedure Enc mapping a bit string *m* to an encoding *c*, and an efficient deterministic decoder algorithm that given an encoding *c* of *m*, outputs *m*. The leakage-resilience guarantee is that for any pair $m,m^{\prime }$, if $c\leftarrow \mathrm{Enc}\left(m\right),c^{\prime }\leftarrow \mathrm{Enc}\left(m^{\prime }\right)$, then any small subset of bits in $c,c^{\prime }$ are identically distributed. (They actually need a stronger leakage-resilience guarantee, see Section 3.1.) Non-explicit constructions of such encodings easily follow from the existence of linear error-correcting codes with sufficiently “good” parameters, such as random linear codes (see, e.g., [14]). In Section 3, we also describe an explicit construction of such encodings.

The leakage-resilience guarantee of an LRE is restricted to protecting the codeword *once it has been encoded*, and does not protect the encoding procedure itself (as opposed to leakage-resilient circuits, see below). Therefore to obtain PCPs with full-fledged ZK against arbitrary query-bounded verifiers, the underlying PCP should possess a zero-knowledge property, which is weaker than full-fledged ZK, but stronger than the HVZK property used in [6,10,11]. Specifically, they use a ZK-PCP variant *over a large alphabet*, which is much easier to obtain compared to full-fledged ZK for standard PCPs, in which the proof is binary.

A ZK-PCP variant over a large alphabet can be thought of as a (standard) PCP which is divided into “regions”, each corresponding to a single symbol in the large alphabet, where ZK is guaranteed only as long as the verifier queries “full” regions (i.e., all bits in the region). Thus, a malicious verifier does not have to follow the honest verifier’s query pattern, but ZK holds only against verifiers querying at most $q^{*}$ full regions, for some a-priori bound $q^{*}$. Such ZK-PCPs are constructed from general secure multi-party computation protocols in [15].

Given a ZK-PCP variant over a large alphabet, [13] design an alphabet reduction that preserves ZK. (Naïve alphabet reduction techniques do not preserve ZK; see Section 3.) This reduction uses the underlying PCP $\left(P^{\prime },V^{\prime }\right)$ as a black box, transforming it into a ZK-PCP $\left(P,V\right)$ in which the proof is over bits. The high-level idea is to interpret each symbol of a proof $\pi^{\prime }$ generated by $P^{\prime }$ as a bit string, and encode it using the LRE. V then emulates $V^{\prime }$, answering an oracle query *i* by reading the entire encoding of the *i*’th symbol from its proof, decoding it, and then providing $V^{\prime }$ with the resultant symbol as the oracle answer.

Hazay et al. [13] then apply their alphabet reduction to the ZK-PCP variant of [15] to obtain the following (see Theorem 3 in Section 3 for the formal statement).

**Informal Theorem** **1.** *There exists a constant ϵ∈(0,1) such that for any ZK parameter $q^{*}\in N$ and any NP-language L there exists a ZK-PCP for L with ZK against $q^{*}$-query bounded verifiers, and a negligible soundness error with a non-adaptive honest verifier that queries ${\left(q^{*}\right)}^{\epsilon }$ proof bits.*

We note that the ZK-PCP system described in Informal Theorem 1 requires a tighter analysis of the ZK-PCP variant of [15], which [13] provide. See Section 3 and [13] for further details.

#### 1.1.2. The ZK-PCPs of Ishai et al.

Ishai et al. [8] construct a non-adaptive ZK-PCP in which the ratio between the bound $q^{*}$ on the query complexity of a malicious verifier, and the query complexity *q* of the honest verifier, is *exponential*, but ZK holds with an inefficient simulator.

Their starting point is a *standard* PCP with no zero-knowledge guarantees. Consequently, to obtain full-fledged ZK, they rely on a stronger leakage-resilient tool. To describe their construction, it would be easier to consider the NP-relation $R=R\left(x,w\right)$ associated with the NP-language L, and its corresponding verification circuit *C*. We will assume without loss of generality that a witness *w* for *x* has canonical form, namely it consists of the entire wire values of *C* given input $x,w^{\prime }$, for some $w^{\prime }$ such that $C\left(x,w^{\prime }\right)=1$. (In particular, $w^{\prime }$ is the information that we wish to keep secret.) The PCP prover generates the proof from this canonical NP witness *w*. Therefore, each proof bit is an information bit on *w*, i.e., on the wire values of *C* when evaluated on $x,w^{\prime }$. This can be thought of as *leakage* on the *computation* in *C*, the purpose of which is to reveal information about the secret input $w^{\prime }$ of *C*. While in general, leakage on the wire values of *C* might reveal information on the secret input $w^{\prime }$, there are tools to compile *C* into a *leakage-resilient* circuit $\hat{C}$ that resists such leakage.

However, it is well known that one cannot protect circuits against *general* polynomial-time leakage [16]. Consequently, leakage-resilient circuits are associated with a restricted leakage class from which leakage functions can be chosen, and the circuit only resists leakage computed by a function from the class. The main observation of [8] is that every PCP system naturally has such a restricted class LEAK of leakage functions associated with it. Indeed, while $V^{*}$ can choose which proof bits to query, it has no control over the *type of functions* applied to *w* to generate the proof bits—these functions are determined solely by the prover algorithm. Specifically, for every subset I of at most $q^{*}$ indices in the proof, the corresponding leakage function $\ell_{I}\in LEAK$ applies the PCP prover function to *w*, then outputs the restriction of the proof to the indices in I.

The main building block is therefore a *Leakage-Resilient Circuit compiler (LRCC)*—a compiler that transforms a given circuit *C* into a leakage-resilient circuit $\hat{C}$—that resists leakage from the class LEAK associated with the underlying PCP system. Informally, an LRCC is associated with a function class LEAK (the *leakage class*) and a (randomized) input encoding scheme $E=\left(\mathrm{Enc},\mathrm{Dec}\right)$, and compiles a deterministic circuit *C* into a deterministic circuit $\hat{C}$ that emulates *C*’s operation over encoded inputs. $\hat{C}$ is guaranteed to emulate *C* on *properly encoded inputs*, and is leakage resilient in the sense that for any pair of inputs $z,z^{\prime }$ for *C* such that $C\left(z\right)=C\left(z^{\prime }\right)$, and any ℓ∈LEAK, the output of *ℓ* on the wire values of $\hat{C}$ when evaluated on a random encoding $\hat{z}\leftarrow \mathrm{Enc}\left(z\right)$ is statistically close to its output when $\hat{C}$ is evaluated on a random encoding $\hat{z^{\prime }}\leftarrow \mathrm{Enc}\left(z^{\prime }\right)$. (We stress that we will only need to consider *stateless* circuits *C*, in which we wish to hide the circuit’s input. We additionally do not allow the leakage-resilient circuit $\hat{C}$ to have *randomized, leak-free* components, and instead the needed randomness will be provided as part of its input encoding.) Notice that if the prover generated the proof from the wire values $\hat{w}$ of $\hat{C}$ (instead of the wire values *w* of *C*), then $V^{*}$’s queries to the proof—which constitute leakage from LEAK on $\hat{w}$—would reveal no information about $w^{\prime }$.

This observation gives a general blueprint for compiling traditional PCPs into ZK-PCPs: given a PCP system $\left(P^{\prime },V^{\prime }\right)$ with an associated leakage class LEAK as described above, and a compiler that transforms a given circuit into one that resists leakage from LEAK, the ZK-PCP system $\left(P,V\right)$ operates as follows. P on input x,w generates the leakage-resilient version $\hat{C_{x}}$ of the circuit $C_{x}=C\left(x,\cdot \right)$ (i.e., *C* with *x* hard-wired into it), uses *w* to generate the entire wire values $\hat{w}$ of $\hat{C_{x}}$, then emulates $P^{\prime }$ on $\hat{w}$ to generate a PCP π. V given input *x* and oracle access to π generates $\hat{C_{x}}$ similarly to P, and emulates $V^{\prime }$ on π to check that $\hat{C_{x}}$ is satisfiable. In particular, whereas the claim which V set out to verify can be phrased as “$C_{x}$ is satisfiable”, the underlying PCP system $\left(P^{\prime },V^{\prime }\right)$ is used to verify a different claim, namely that *the leakage-resilient circuit $\hat{C_{x}}$* is satisfiable.

Unfortunately, this blueprint does not actually work. The reason is that the internal system $\left(P^{\prime },V^{\prime }\right)$ is used to prove a *different* statement, namely that $\hat{C_{x}}$ is satisfiable. For $\left(P,V\right)$ to be sound, it should be the case that $\hat{C_{x}}$ is satisfiable only if $C_{x}$ is. This, however, is not generally guaranteed by LRCCs, as we now explain. Recall that the LRCC is correct for properly encoded inputs, in the sense that on such inputs, $\hat{C}$ emulates *C*. However, LRCCs in general have no guarantee for inputs which are *not* properly encoded. This is not just an artifact of the definition, but is rather essential for leakage resilience to hold in existing constructions. A main technical contribution of [8] is in defining and constructing an LRCC that also guarantees soundness, in the sense that the leakage-resilient circuit $\hat{C}$ is satisfiable (even by using invalid encodings as inputs) only if the original circuit *C* is satisfiable.

To turn this into an actual construction, one needs to design a sound LRCC for a leakage class LEAK associated with some standard PCP system. The most common leakage classes considered in the literature are either the “Only Computation Leaks” (OCL) model that assumes leakage is “local” in the sense that different “regions” of the circuit leak independently [17], or classes of functions that are “computationally simple” [18], i.e., from a low complexity class such as $AC^{0}$. It is known that the leakage classes associated with a PCP system cannot be of the former type [19], so Ishai et al. [8] focus on the latter. Specifically, they show that the PCP system of Arora and Safra [2] has the property that “small” subsets of proof bits can be generated using the class LEAK of $AC^{0}$ circuits (i.e., constant-depth polynomial-sized circuits with ∧,∨,¬ gates of unbounded fan-in and fan-out) augmented with a small number of ⊕ gates. (See Section 4.3 for a formal definition of this leakage class.) Then, they use correlation bounds of Lovett and Srivinasan [20] to show that their sound LRCC resists leakage from LEAK. This yields the following result, where a witness-indistinguishable PCP is a ZK-PCP in which the ZK property holds with an *inefficient* simulator (see Theorem 7 in Section 4 for the formal statement).

**Informal Theorem** **2.** *For any ZK parameter $q^{*}\in N$ and any NP-language L there exists a witness-indistinguishable PCP for L with witness-indistinguishability against $q^{*}$-query bounded verifiers, and a negligible soundness error with a non-adaptive honest verifier that queries $\mathrm{polylog}\left(q^{*}\right)$ proof bits.*

Assuming the existence of one-way functions (a minimal assumption in cryptography), as well as a Common Random String (CRS) that is available to both parties, and using a standard cryptographic technique—the so-called “FLS technique” [21]—the witness-indistinguishable PCP of Informal Theorem 2 can be transformed into a *ZK*-PCP system where ZK holds against *computationally-bounded* query-bounded verifiers in the CRS model. (We refer the interested reader to [8] for further details, including a formal definition of the model).

### 1.2. Comparison between Different ZK-PCP Constructions

The ZK-PCPs of [13] (Informal Theorem 1) obtain ZK with efficient simulation as in the ZK-PCPs of [6,11], with a query gap of $q^{*}/{\left(q^{*}\right)}^{\epsilon }$. The query gap is inherited directly from the underlying ZK-PCP variant of [15], which requires the honest verifier to query $\Omega \left({\left(q^{*}\right)}^{\epsilon }\right)$ proof symbols to obtain a negligible soundness error. Therefore, the query gap could potentially be improved by replacing the underlying building block with a ZK-PCP variant with a larger query gap.

On the other hand, the witness-indistinguishable PCPs of [8] (Informal Theorem 2) obtain an exponential query gap, similar to the ZK-PCPs of [6,11]. This is possible because the underlying (non-ZK) PCP has a negligible soundness error with an honest verifier that queries a poly-logarithmic number of proof bits. However, the construction is only witness-indistinguishable, which is weaker than ZK (see Section 2.1.1). Ref. [8] show that unless NP=BPP, obtaining *ZK*-PCPs (i.e., with efficient simulation) using their technique would require a new and entirely different approach towards designing and analyzing security of leakage-resilient circuits. We further note that while the techniques of [6,13] extend also to PCPs *of Proximity* (see Section 2.1.1 for a description of this model, and [11,13] for the constructions), the technique of [8] does not seem to readily lend itself to designing zero-knowledge (or even witness-indistinguishable) PCPs of proximity.

Finally, all the aforementioned ZK-PCP constructions [6,8,11,13] have polynomial-length proofs (whereas traditional PCPs can have quasi-linear length). However, while a polynomial blowup in proof length is inherent to the constructions of [6,11] due to their use of locking schemes, this is not the case for the leakage resilience based constructions [8,13], which could potentially have shorter proofs. This is an additional advantage of taking the leakage resilience based approach. The discussion is summarized in Table 1.

### 1.3. Related Notations, Extensions, and Cryptographic Applications

Many different variants of proof systems and ZK proof systems have been considered in the literature (see, e.g., Thaler’s survey [22], and references therein). We briefly mention two notable notions that are closely related to ZK-PCPs; see also Table 2. The first is *Interactive Oracle Proofs (IOPs)* [23,24] (a special case of IOPs appeared earlier in [25]) which combine aspects of IPs and PCPs. Specifically, in an IOP the verifier and prover interact as in an IP, but the verifier has oracle access to prover messages as in PCPs. These proof systems have received increasing attention, partly due to their uses in blockchain applications. Unlike ZK-PCPs, which can be obtained from standard PCPs through generic compilers, existing ZK-IOPs (see, e.g., [25,26] and references therein) are constructed in an ad-hoc manner, in which ZK is “tailored” to a specific non-ZK IOP. (It is of course preferable to have a generic compiler, since it can be used to enhance any new IOP construction to also guarantee ZK.) The second notion is PCPs *of Proximity (PCPPs)* [3,7,27]—a generalization of PCPs in which the verifier does not read its entire input. Instead, V has oracle access to x,π, and wishes to check whether *x* is close to L in relative Hamming distance. *Zero-Knowledge* PCPPs (ZK-PCPPs) [11] extend ZK-PCPs to the PCPP realm. They guarantee that the view of any verifier $V^{*}$ making $q^{*}$ queries *to the input and the proof* can be efficiently simulated, up to a small statistical distance, *by making only $q^{*}$ queries to the input*. Ishai and Weiss [11] construct ZK-PCPPs for NP with comparable parameters to the ZK-PCPs of [6,10], where soundness holds for inputs which are δ-far from the language, for δ which is constant or inverse polylogarithmic. The honest verifier in their construction is adaptive. Hazay et al. [13] show how to extend their techniques to the setting of PCPPs, constructing ZK-PCPPs for NP with a polynomial query gap with a *non-adaptive* honest verifier.

ZK-PCPs (and ZK-PCPPs) are motivated not only from a purely theoretical perspective as a natural model of a proof system, but also from their usefulness for cryptographic applications. Specifically, they enable modular design of cryptographic proofs systems, by separating a “clean” information–theoretic proof system component, from the cryptographic assumptions (such as hardness assumptions or an augmented model of computation) which can then be used to transform—i.e., “compile” through a *cryptographic compiler*—the information–theoretic system into a computationally-secure system than can be implemented. Thus, one can design, analyze and optimize the information–theoretic proof system, then apply different cryptographic compilers to obtain different properties of the resultant system. This paradigm has been extremely successful, and is widely used. For example, ZK-PCPs are the underlying combinatorial building blocks in constructions of succinct zero-knowledge arguments [29].

ZK-PCPs and ZK-PCPPs also have more direct cryptographic applications both to two-party and multiparty scenarios which require highly efficient verification methods on secret data. In the two-party setting, these include constructions of constant-round (or non-interactive in the random-oracle model) black box arguments for NP with statistical ZK [10] (whereas constructions based on *non*-ZK PCPs are not black box [30,31]). ZK-PCPPs can additionally be used to design two-party and multiparty black box commit-and-prove protocols for NP [11]. While these applications only require a weaker ZK guarantee (specifically, ZK against the *honest* verifier), applications in multiparty settings require full-fledged ZK against *malicious* verifiers. These include constructions of certifiable versions of verifiable secret sharing from ZK-PCPPs, as well as sublinear ZK proofs in a distributed setting from ZK-PCPs. (Various other notions of ZK proofs in a distributed setting have been considered recently.) In the latter application, the prover and verifier are aided by multiple (potentially corrupted) servers. The motivation for this distributed setting is to minimize the round complexity, and underlying assumptions, of sublinear ZK proofs. Specifically, using PCPs with ZK against *malicious* verifiers, [8] construct distributed 3-round witness-indistinguishable proofs (respectively, ZK proofs in the computational setting with a common random string) for NP, which are unconditionally secure (respectively, based on the existence of one-way functions) in which the total communication involving the verifier is sublinear in the input length. This should be contrasted with standard sublinear ZK arguments, that require at least four rounds of interaction, and require the existence of collision resistant hash functions [10,30].

## 2. Preliminaries

**Basic notations.** We denote the security parameter by κ. A function μ:N→N is *negligible* if for every positive polynomial $p\left(\cdot \right)$ and all sufficiently large κ’s it holds that $\mu \left(\kappa \right)<\frac{1}{p(\kappa )}$, and $\mathrm{negl}\left(\kappa \right)$ denotes the set of all negligible functions. We use the asymptotic notations $O\left(\cdot \right)$ and $\Omega \left(\cdot \right)$, where $\tilde{O}\left(n\right)$ and $\tilde{\Omega }\left(n\right)$ denote $n\cdot \mathrm{poly}\left(\log\right)n$ and $n/\mathrm{poly}\left(\log\right)n$, respectively. We use the abbreviation PPT to denote Probabilistic Polynomial-Time, and denote by $\left[n\right]$ the set of elements $\left\{1,\ldots ,n\right\}$. For a string *s* of length *n*, and a subset $I\subseteq \left[n\right]$, we denote by ${s|}_{I}$ the restriction of *s* to the coordinates in *I*.

We usually denote vectors using boldface letters (e.g., a), or as $\vec{a}$. For a pair x,y of vectors, 〈x,y〉 denotes their inner product, and $\mathrm{Ham}\left(x,y\right)$ to denote their Hamming distance, i.e., $\mathrm{Ham}\left(x,y\right)=\left|\left\{i\;:x_{i}\neq y_{i}\right\}\right|$. For functions f,g, we denote their composition as $\left(f\circ g\right)\left(x\right):=f\left(g\left(x\right)\right)$. The composition of families F,G of functions is defined as $F\circ G:=\left\{f\circ g:f\in F,g\in G\right\}$.

For a distribution D, sampling according to D is denote by X←D, or $X\in_{R}D$. For a pair of random variables X,Y, we use X≡Y to denote that X,Y are identically distributed. For random variables $X_{\kappa }$ and $Y_{\kappa }$ over a finite domain Ω, the *statistical distance* between them is defined as

$$\mathrm{SD}\left(X_{\kappa },Y_{\kappa }\right)=\frac{1}{2}\sum_{w\in \Omega }|\mathrm{Pr}\left[X_{\kappa }=w\right]-\mathrm{Pr}\left[Y_{\kappa }=w\right]|.$$

$X_{\kappa }$ and $Y_{\kappa }$ are *ϵ-statistically close* if their statistical distance is at most ϵ(κ). Ensembles ${\left\{X_{\kappa }\right\}}_{\kappa },{\left\{Y_{\kappa }\right\}}_{\kappa }$ are *statistically close*, denoted $X_{\kappa }\approx Y_{\kappa }$, if there exists an $\epsilon \left(\kappa \right)=\mathrm{negl}\left(\kappa \right)$ such that $X_{\kappa },Y_{\kappa }$ are $\epsilon \left(\kappa \right)$-close for every κ. We say that ${\left\{X_{\kappa }\right\}}_{\kappa },{\left\{Y_{\kappa }\right\}}_{\kappa }$ are *computationally indistinguishable* if they have a $\mathrm{negl}\left(\kappa \right)$*computational distance*, i.e., for every PPT distinguisher D there exists an $\epsilon \left(\kappa \right)=\mathrm{negl}\left(\kappa \right)$ such that for every κ:

$$\left|\mathrm{Pr}\left[D\left(X_{\kappa }\right)=1\right]-\mathrm{Pr}\left[D\left(Y_{\kappa }\right)=1\right]\right|\leq \epsilon \left(\kappa \right).$$

**Languages and Relations.** We will consider NP-relations $R=R\left(x,w\right)$, and the corresponding NP-languages $L=\left\{x\;:\;\exists w\;s.t.\;\left(x,w\right)\in R\right\}$. We sometimes write $R_{L}$ to refer to the NP-relation, the corresponding language of which is L.

**Encoding Schemes and Leakage-Resilient Encoding Schemes.** Our constructions will rely on encodings schemes with leakage-resilience properties. We now provide a simple definition of an encoding scheme, and refer the reader to Section 3.1 for a more detailed discussion of the notion.

**Definition** **1.** *Let k,n∈N. An* Encoding Scheme *over an alphabet Σ is a pair $\left(\mathrm{Enc},\mathrm{Dec}\right)$ where Enc is a PPT algorithm, and Dec is a (deterministic) polynomial-time algorithm, that satisfy the following.*

- **Syntax.** *Enc on input a secret $x\in \Sigma^{k}$ outputs a codeword $c\in \Sigma^{n}$. Dec on input $c\in \Sigma^{n}$ outputs $x\in \Sigma^{k}$ or a special error symbol* ⊥.
- **Correctness.** *There exists a t≥0 such that the following holds for every $x\in \Sigma^{k}$, and every $c\in \Sigma^{n}$: if there exists $c_{x}\in \mathrm{Supp}\left(\mathrm{Enc}\left(x\right)\right)$ such that $\mathrm{Ham}\left(c,c_{x}\right)\leq t$ then $\mathrm{Dec}\left(c\right)=x$, otherwise Dec outputs* ⊥.

**A note on terminology.** In this section and in Section 3, we use k,n respectively to denote the input and output lengths of Enc, as is customary in the context of error-correcting codes. In Section 4, the input and output lengths are denoted by $n,\hat{n}$, respectively, which is sometimes used in the context of leakage resilience.

We now define two useful properties of encoding schemes. The first is that the scheme is *linear*. The second is that the scheme is *onto*, meaning any $c\in \Sigma^{n}$ can be interpreted as the encodings of *some* $x\in \Sigma^{k}$, in the sense that *c* would be decoded to *x*.

**Definition** **2.** *An encoding scheme $\left(\mathrm{Enc},\mathrm{Dec}\right)$ over a field F is* linear *if for every k: (1) k divides n; and (2) there exists a decoding vector d such that for every $x\in F^{k}$: (1) every $x\in \mathrm{Supp}\left(\mathrm{Enc}\left(x\right)\right)$ can be partitioned into k sub-vectors $x=\left(x^{1},\ldots ,x^{k}\right)$, such that $\mathrm{Dec}\left(x\right)=\left(\langle d,x^{1}\rangle ,\ldots ,\langle d,x^{k}\rangle \right)$.*

*$\left(\mathrm{Enc},\mathrm{Dec}\right)$ is* onto *, if Dec is defined (i.e., does not output* ⊥*) for every $c\in \Sigma^{n}$.*

Section 4 will use a *parameterized* notion of encoding schemes. In such encoding schemes, the encoding and decoding algorithms are given an additional input $1^{\sigma }$, which is used as a security parameter. The encoding length can then depend also on σ (and not only on *k*), and we require that for every σ the resultant scheme is an encoding scheme. A parameterized encoding scheme is onto (linear, respectively) if it is onto (linear, respectively) for every σ.

We now define leakage resilience of distributions and encodings.

**Definition** **3** (Leakage Resilience—Distributions and Encoding Schemes). *Let LEAK be a family of functions, and ϵ>0. For a finite set D, a pair of distributions X,Y over D are*$\left(LEAK,\epsilon \right)$-leakage resilient *if for any function ℓ∈LEAK with domain D it holds that $\mathrm{SD}\left(\ell \left(X\right),\ell \left(Y\right)\right)\leq \epsilon$.*

*A randomized function $f:\Sigma^{n}\to \Sigma^{m}$ is*$\left(LEAK,\epsilon \right)$-leakage resilient *if for every $x,y\in \Sigma^{n}$, the distributions $f\left(x\right),f\left(y\right)$ are $\left(LEAK,\epsilon \right)$-leakage resilient.*

*An encoding scheme $\left(\mathrm{Enc},\mathrm{Dec}\right)$ is*$\left(LEAK,\epsilon \right)$-leakage resilient *if for every large enough σ∈N, $\mathrm{Enc}\left(\cdot ,1^{\sigma }\right)$ is $\left(LEAK,\epsilon \right)$-leakage resilient.*

For the construction of Section 3 we will be particularly interested in *probing-resilient* encoding schemes, namely ones that are leakage resilient against leakage functions that probe bits of the codeword.

For a function family LEAK, we sometimes use the term “leakage family LEAK”, or “leakage class LEAK”, and refer to functions in LEAK as “leakage functions”.

### 2.1. PCPs and ZK-PCPs

The probabilistic proof system we focus on in this work is *Probabilistically Checkable Proofs (PCPs)* with zero-knowledge guarantees. We first describe the (standard, non-zero-knowledge) notion of PCPs.

At a high level, PCPs allow a randomized verifier to probabilistically verify the validity of some input statement by querying few bits of a purported proof to which it has oracle access. PCPs can be defined (and have been studied) in relation to various complexity classes (e.g., $DTIME\left(n\right)$). In this work, we focus on PCPs for NP since cryptographic applications usually require proof systems for NP (and this also simplified the presentation). In the context of complexity theory, the system consists solely of the verifier and the proof oracle, where the proof generation process is implicit. However, cryptographic applications necessitate that proof generation be efficient, given the NP-witness. Thus, we define PCPs as a system consisting of efficient prover and verifier. (This is by now standard in the literature of PCPs for cryptographic applications, e.g., [6], as well as other proof systems such as interactive oracle proofs [23,24].)

More specifically, a PCP system for a language L∈NP consists of a polynomial-time prover P that given x∈L and a corresponding witness generates a proof π for *x*, and a PPT verifier V having direct access (“oracle access”) to individual symbols of π. V will read only part of its proof string π (called oracle), where the queries to π are determined by V’s input and coin tosses. Formally,

**Definition** **4** (PCP). *A* Probabilistically Checkable Proof (PCP) *for a language L∈NP consists of a polynomial-time prover P and a*PPT*verifier V such that there exists a negligible function $\mathrm{ngl}=\mathrm{negl}\left(\kappa \right)$ for which the following holds:*

- **Syntax:** *The prover P has input $1^{\kappa },x,w$ (κ is the security parameter), and outputs a proof $\pi \in {\left\{0,1\right\}}^{*}$ for x. The verifier V has input $1^{\kappa },x$, and oracle access to π. It makes q queries to π, and outputs either 0 or 1 (representing reject or accept, respectively). q is called the* query complexity *of the system, and the system is called aq*-query PCP.
- **Semantics:***The system satisfies the following semantic properties:*
  - **Completeness:** *For every $\left(x,w\right)\in R_{L}$, and every proof $\pi \in P\left(1^{\kappa },x,w\right)$,*
    $$\mathrm{Pr}[V^{\pi }\left(1^{\kappa },x\right)=1]=1,$$
    *where the probability is over the randomness of V.*
  - **Soundness:** *For every x∉L and every oracle $\pi^{*}$,*
    $$\mathrm{Pr}\left[V^{\pi^{*}}\left(1^{\kappa },x\right)=1\right]\leq \mathrm{negl}\left(\kappa \right),$$
    *where the probability is over the coin tosses of the verifier. $\mathrm{negl}\left(\kappa \right)$ is called the* soundness error *of the system.*

**Zero-Knowledge PCPs (ZK-PCPs).** Intuitively, ZK-PCPs are PCPs in which the witness remains entirely hidden throughout the verification procedure, in the sense that even a *malicious* verifier which deviates from the specified verification procedure learns nothing but the validity of the claim. Achieving this property requires some modifications to the standard notion of a PCP. First, the prover must now be *probabilistic*. Indeed, completeness of the PCP requires (intuitively, at least) that the proof encode the witness. That is, certain proof bits will carry *some* information about the witness, and by querying them a malicious verifier $V^{*}$ may learn information about the witness. Allowing randomized proof generation is akin to having a probabilistic prover in zero-knowledge *interactive* proofs (whereas in standard interactive proofs the prover may be deterministic).

Second, while we do not impose any restrictions on the query *pattern* (i.e., the strategy) of a malicious verifier $V^{*}$, we impose a bound on its query *complexity*, namely the number of queries it makes to the proof. To see why imposing *some* restriction is needed, recall that cryptographic applications necessitate an efficient honest prover, meaning the proof will have polynomial length. Therefore, a polynomial-time verifier $V^{*}$ that is unrestricted in its access to the proof could potentially read the entire proof and thus necessarily learn information about the witness (since, as noted above, the proof carries information about the witness). There are several possible methods of restricting $V^{*}$ to reading only *part* of the proof (e.g., requiring its runtime to be smaller than the proof length), where the one used in the literature is to restrict its *query complexity* to some a-priori bound $q^{*}$ which is known prior to proof generation (in particular, the proof length may depend on $q^{*}$). One advantage of restricting the query complexity is that it allows us to obtain *information theoretic* ZK, namely that ZK holds against malicious verifiers with *unbounded computational power*, so long as the verifier makes at most $q^{*}$ queries to the proof. It is also more inline with the main efficiency measures of standard PCPs, which focus on the query (and randomness) complexity.

To define ZK-PCPs, we first formalize the restriction on the query complexity of the verifier.

**Definition** **5** (Query-bounded verifier). *We say that a (possibly malicious) verifier $V^{*}$ with oracle access to a proof π is*$q^{*}$-query-bounded *if it makes at most $q^{*}$ queries to π.*

As noted above, we will allow a malicious verifier to be computationally unbounded. Moreover, we will allow its query bound $q^{*}$ to be much larger than that of the honest verifier. Ideally, the honest verifier will only make $\mathrm{polylog}\left(q^{*}\right)$ queries, and the proof will have length $\mathrm{poly}\left(q^{*}\right)$. We note that traditional PCPs can be verified with a constant number of queries, achieving a constant soundness error. However, since cryptographic applications usually necessitate a *negligible* soundness error, the query complexity of the honest verifier is necessarily polylogarithmic in the security parameter κ, or in $q^{*}$.

Another aspect in which we allow a malicious verifier to be more powerful than the honest verifier is *adaptivity*: whereas we would like the honest verifier to be non-adaptive—namely, make a single round of queries to the proof (as in standard PCPs), we allow a malicious verifier to be adaptive—i.e., make several rounds of queries to the proof. This is formalized in the following definition.

**Definition** **6** (Adaptive and-non adaptive verifiers). *We say that a (possibly malicious) verifier $V^{*}$ is* non adaptive *if its queries are determined solely by its input x and randomness (in particular, $V^{*}$ can make all its queries to the proof in a single round). Otherwise, we say that $V^{*}$ is* adaptive *(in particular, the queries of an adaptive verifier may depend on the answers to previous queries).*

We are now ready to define ZK-PCPs. Similar to zero-knowledge *interactive* proofs (and, more generally, cryptographic protocols), we formalize zero knowledge by requiring the existence of an efficient *simulator* algorithm that, given the input $1^{\kappa },x$, can simulate the *view* of the verifier, namely the verifier’s input, random coins, and the oracle answers (the queries of the verifier can be computed from these values). Intuitively, this guarantees zero knowledge because the view of the verifier captures the entire “knowledge” it obtained through verification, and in particular by querying the proof oracle. Since the view can be simulated from the input alone, without any access to the NP-witness, this implies that the view contains no information about the witness. We now formalize this intuition.

**Notation** **1.** *For a PCP system $\left(P,V\right)$ and a (possibly malicious) verifier $V^{*}$, we use ${\mathrm{View}}_{V^{*},P}\left(\kappa ,x,w\right)$ to denote the view of $V^{*}$ when it has input $1^{\kappa },x$ and oracle access to a proof that was randomly generated by P on input $\left(1^{\kappa },x,w\right)$.*

**Definition** **7** (ZK-PCP). *We say that a PCP system $\left(P,V\right)$ for L is a*$\left(q^{*},\varepsilon \right)$-Zero-Knowledge PCP (ZK-PCP)*if for every (possibly malicious and adaptive) $q^{*}$-query-bounded verifier $V^{*}$ there exists a*PPT*simulator Sim, such that for every $\left(x,w\right)\in R$, $\mathrm{Sim}(1^{\kappa },x)$ is distributed ε-statistically close to ${\mathrm{View}}_{V^{*},P}\left(x,w\right)$.*

One prevalent approach for designing simulators (which we will use) is to have the simulator emulate the verifier $V^{*}$, simulating the answers to $V^{*}$’s oracle queries.

#### 2.1.1. Restrictions, Extensions and Generalizations

Definition 7 can be restricted, or alternatively, generalized, in several ways, as we now discuss.

**A taxonomy based on ZK quality.** While we define a statistical notion of ZK as the default for ZK-PCPs, one can also consider stronger or weaker forms of ZK. Specifically, we can require a stronger *perfect* ZK guarantee in which the simulated view is distributed identically to the real view of the verifier.

**Notation** **2.** *We say that a ZK-PCP has*$q^{*}$-ZK *if it has perfect ZK against $q^{*}$-query bounded verifiers.*

Alternatively, we can settle for a weaker *computational* ZK guarantee which only holds against *computationally-bounded* verifiers $V^{*}$, where the simulated view is computationally indistinguishable from the real view. One particular relaxation of ZK which we consider in this work is *Witness-Indistinguishability* which does not require that the ZK simulator be efficient. This can be obtained by removing the requirement that Sim be PPT in Definition 7, but the following alternative formulation would be more useful.

**Definition** **8** (WI-PCP). *We say that a PCP system $\left(P,V\right)$ for L is a*$\left(q^{*},\varepsilon \right)$-Witness- Indistinguishable PCP (WI-PCP)*if for every (possibly malicious and adaptive) $q^{*}$-query-bounded verifier $V^{*}$, for any x∈L, and any pair of corresponding witnesses $w_{1},w_{2}$ such that $\left(x,w_{1}\right),\left(x,w_{2}\right)\in R_{L}$:*

$$\mathrm{SD}\left({\mathrm{View}}_{V^{*},P}\left(x,w_{1}\right),{\mathrm{View}}_{V^{*},P}\left(x,w_{2}\right)\right)\leq \varepsilon .$$

There are also various flavors of ZK based on different qualities of the ZK simulator. These include, for example, whether the simulator is straight line—i.e., it emulates the verifier without having to rewind it, and whether the simulator interacts with the verifier as a black box. The ZK-PCP constructions described in this work have straight line, black box simulators.

**Honest-Verifier ZK.** Another natural restriction of the definition is by considering zero knowledge only against the *honest verifier* V. Such systems are called *Honest-Verifier ZK (HVZK)*. As we explain below, it is fairly simple to obtain an HVZK PCP system from a standard PCP system (e.g., such a system was presented already in the paper of Kilian, Petrank and Tardos on ZK-PCPs [6], and a weaker construction with a large soundness error was presented in [9]). However, such a restriction is generally too weak to be used in cryptographic applications, for example, to prove honest behaviour in cryptographic protocols, in which case the verifying party might be maliciously corrupted. To see why settling for HVZK simplifies the problem considerably, notice that HVZK is preserved under standard soundness amplification techniques. More specifically, assume we have an HVZK PCP system with a large soundness error (obtaining such systems is relatively easy given known techniques such as “MPC-in-the-head” [15]). Soundness can then be amplified by having the prover generate many fresh, independent copies of the proof, and having the honest verifier repeat the verification procedure several times, each time using a fresh proof copy. HVZK of this verification procedure easily reduces to the HVZK of the original system. However, this amplification does not preserve *full-fledged* ZK (i.e., against malicious verifiers) *even if* the original system is ZK against malicious verifiers. Indeed, the reason is that the query complexity—even of the honest verifier—increases through this transformation, and in particular would exceed the ZK query bound of the original system. Thus, a malicious verifier that “concentrates” all its queries to a *single* proof copy might be able to violate the ZK of the underlying system.

**Verifier Adaptivity.** In *non-ZK* PCP constructions, the honest verifier is non-adaptive. In contrast, the classic ZK-PCP of [6], and all consequent ZK-PCPs—*except the ones based on leakage resilience* [8,13]—have an *adaptive* honest verifier. As discussed in Section 1, this is because, very roughly, they enhance an HVZK PCP to have full-fledged ZK, by modifying the proof such that even honest verification requires multiple rounds of queries to the proof. Moreover, this is inherent to their technique of using “locking schemes” [6]. Having a non-adaptive honest verifier is a major advantage of leakage resilience based ZK-PCPs (see Table 1 for a comparison of existing ZK-PCP systems), since having non-adaptive *honest* verification is a desirable feature of the system. Indeed, an adaptive honest verifier translates into multiple interaction rounds in cryptographic applications of ZK-PCPs.

**Notation** **3.** *We say that a ZK-PCP system $\left(P,V\right)$ is a* non-adaptive ZK-PCP *if the honest verifier V is non-adaptive.*

We note that an orthogonal measure of adaptivity is whether a *malicious* verifier is restricted to being non-adaptive. Unlike having a non-adaptive honest verifier, guaranteeing ZK only against non-adaptive malicious verifiers is an *undesirable restriction* of the system, since it means there are no guarantees against adaptive verifiers.

**Universal vs. Non-Universal Simulation.** Definition 7 requires ZK to hold with a non-universal simulator, requiring, for every malicious verifier $V^{*}$, the existence of a simulator ${\mathrm{Sim}}_{V^{*}}$. A stronger possible definition would require the existence of a *universal* simulator Sim that can simulate the view of *any* query-bounded verifier $V^{*}$. We note that all the ZK-PCPs described in this work (in fact, to the best of our knowledge, all existing ZK-PCP constructions) satisfy this stronger definition.

**The Alphabet.** Similar to PCPs, ZK-PCPs are defined as bit-strings. One could also consider a relaxed notion in which the proof is over some larger alphabet Σ (and this indeed has been done in the PCP context). We note, however, that while for standard PCPs the choice of alphabet affects only the parameters of the scheme (but not its its semantic properties), this is not the case for *zero-knowledge* PCPs. Indeed, any PCP over Σ can be transformed to a PCP over {0,1} by replacing each symbol with a bit-string representation of it, without violating completeness or soundness. (We note that while more elaborate alphabet reduction techniques have been employed in the context of traditional PCP, e.g., in [3], their goal was to improve the system’s parameters.) However, as we explain in Section 3, doing so for a *ZK*-PCP does not preserve ZK against *malicious* verifiers. ZK-PCPs over a large alphabet Σ can still be useful as a building block for obtaining ZK-PCPs (with proofs over {0,1}), see Section 3.

**PCPs of Proximity.** A useful generalization of PCPs are PCPs of *Proximity* (PCPPs), that allow verification of an input claim while reading only a small portion of it. This is formalized by giving the verifier oracle access to the input, similar to how it accesses the proof. Of course, in this case the verifier cannot be expected to distinguish a true claim from a claim that is false, but very close to being true (e.g., a 3-CNF for which there exists an assignment that satisfies all but a tiny fraction of the clauses). Instead, soundness is defined similarly to correctness of promise problems: any input which is sufficiently far from the corresponding NP-language will be rejected with high probability. PCPPs are an important building block in PCP constructions, and a useful notion in its own right. There are PCPP constructions matching the properties of the best-known standard PCPs [32,33].

*Zero-Knowledge PCPPs (ZK-PCPPs)* [11] have a stronger ZK guarantee than ZK-PCPs: while ZK-PCPs guarantee that the *witness* remains entirely hidden through verification, ZK-PCPPs additionally guarantee that the *input* itself remains mostly hidden, in the sense that the verifier (even a malicious one) learns only few *physical* input bits. This is formalized using the simulation paradigm as in ZK-PCPs, where instead of giving the entire input to the simulator, it has oracle access to it, and is restricted to making $q^{*}$ queries (where $q^{*}$ is the query complexity of the verifier).

Certain techniques for constructing ZK-PCPs extend also to PCPPs, while others do not (or, at least, it is not clear how to extend them). In particular, the original ZK-PCP construction of [6] can be extended to also apply to ZK-PCPPs [11], and the ZK-PCPs based on leakage-resilient encodings described in Section 3 also applies to PCPPs (see [13] for a full description of the construction). On the other hand, the construction of ZK-PCPs from LR circuits (Section 4) does not seem to easily extend to the PCPP realm.

## 3. The ZK-PCPs of Hazay et al.: ZK from LR Encodings

The main result of this section is a construction of ZK-PCPs for NP with a non-adaptive honest verifier and a polynomial query gap (between the query complexity of the honest and malicious verifiers) due to [13]:

**Theorem** **3** (ZK-PCPs for NP, Formal statement of Informal Theorem 1). *There exists a constant ϵ∈(0,1) such that for any ZK parameter $q^{*}\in N$ there exists a non-adaptive ${\left(q^{*}\right)}^{\epsilon }$-query $\Omega \left(q^{*}\right)$-ZK-PCP for NP.*

We describe a simplified version of the construction of [13] which nonetheless suffices for designing ZK-PCPs. The interested reader is referred to [13] for a description of the more general paradigm which employs an equivocal notion of secret sharing instead of the weaker leakage-resilient encodings used here.

The construction employs Leakage-Resilient (LR) Encodings. The starting point is a ZK-PCP variant $\left(P^{\prime },V^{\prime }\right)$ over a *large alphabet Σ*, namely where the proof $\pi^{\prime }$ is over Σ. To obtain a standard ZK-PCP $\left(P,V\right)$—i.e., one in which the proof π is over *bits*—we need an *alphabet reduction*. That is, we are looking for a transformation that replaces each symbol $\pi_{i}^{\prime }\in \Sigma$ with a bit-string “segment” ${\mathrm{segm}}_{i}\in {\left\{0,1\right\}}^{*}$. Then, given a proof $\pi^{\prime }=\left(\pi_{1}^{\prime },\ldots ,\pi_{N}^{\prime }\right)$, the resultant proof would be $\pi =\left({\mathrm{segm}}_{1},\ldots ,{\mathrm{segm}}_{N}\right)$.

As mentioned in Section 2.1.1, the naive alphabet reduction which replaces each symbol of Σ with a bit string representing it does not preserve ZK. Indeed, this alphabet reduction necessarily increases the query complexity of the honest verifier V, who will need to query $q\cdot \left|\mathrm{segm}\right|$ proof bits (where *q* is the query complexity of $V^{\prime }$). Thus, a malicious verifier—the query complexity of which is at least as that of the honest verifier—with oracle access to the resultant proof π may query subsets of bits in *many* segments ${\mathrm{segm}}_{i}$, effectively learning partial information about many proof symbols (in particular, more than the ZK guarantee of $\pi^{\prime }$), and violating ZK. Therefore, we need an alphabet reduction which *preserves ZK.*

Viewed through the leakage-resilience lens, the information which a malicious verifier obtains on a symbol $\pi_{i}^{\prime }\in \Sigma$ by querying bits of ${\mathrm{segm}}_{i}$ constitutes *probing leakage* on ${\mathrm{segm}}_{i}$, and consequently also on $\pi_{i}^{\prime }$. Thus, intuitively, ZK can be guaranteed by protecting the segments ${\mathrm{segm}}_{i}$ from probing leakage. This gives a general blueprint for a ZK alphabet reduction: replace each symbol σ of Σ with its binary representation $s_{\sigma }$, then encode $s_{\sigma }$ using a probing-resilient encoding. While this roughly describes the alphabet reduction of [13], there are a few subtleties, as we now describe.

**Simulation Strategy for Malicious Verifiers.** A probing-resilient encoding can only protect against probing of a sufficiently small subset of bits of the encoding, namely against probing of some a-priori fixed fraction τ of bits. (Indeed, since the message can be decoded from the encoding, an adversary that probes the entire encoding necessarily learns the underlying message.) However, a malicious verifier $V^{*}$ may query an entire segment ${\mathrm{segm}}_{i}$. To see why, notice that the query bound $q^{*}$ imposed on the malicious verifier is expected to be much larger than the length of the encoding. Indeed, $q^{*}$ should be at least as large as the query complexity of the *honest* verifier, which would need to read at least a few symbols of the original PCP, i.e., a few *full* encodings of symbols of the original PCP. More generally, $V^{*}$ may read more than a τ-fraction of a segment, in which case the probing-resilience of the encoding cannot be used. We solve this issue in the simulation by dividing the segments ${\mathrm{segm}}_{i}$ into two types: “heavy” and “light” segments. Intuitively, heavy segments are ones from which $V^{*}$ queried many bits, in particular, more than a τ-fraction. Light segments are segments that are not heavy. We use the probing-resilience of the underlying encoding to claim that $V^{*}$ learns no information about the symbols encoded in the light segments, and use the ZK guarantee of the underlying ZK-PCP system $\left(P^{\prime },V^{\prime }\right)$ to simulate the symbols encoded in heavy segments. This gives us a simulation strategy for $\left(P,V\right)$: simulate heavy symbols using the simulator of the underlying system $\left(P^{\prime },V^{\prime }\right)$, and simulate light symbols using random and independent encoding of an arbitrary value (e.g., the all-zeros string).

**Simulating Partially-Leaked Symbols.** The simulation strategy defined in the previous paragraph necessitates that the simulator Sim knows in advance which segments are heavy and which are light, since this determines how to generate the answer to a query. Whether or not a segment is heavy is a function of *the entire query pattern* of $V^{*}$. Thus, this proof strategy only works against *non-adaptive* malicious verifiers, namely ones which make a single round of queries to the proof. (Indeed, in this case Sim learns all of $V^{*}$’s queries before it needs to simulate the oracle answers.) ZK against *adaptive* malicious verifiers, namely ones which make several rounds of queries to the proof, where each query may depend on the answers to the previous queries, requires a somewhat different simulation strategy, and a stronger LR guarantee from the probing-resilient encoding, as we now explain.

The high-level idea is as follows. At the onset of the simulation, Sim treats all segments as light, answering queries using random and independent encodings of $\vec{0}$. At certain points in the simulation, a certain segment *i* may become heavy—namely, the number of queries $V^{*}$ made to it exceeds the probing threshold τ. At this point, Sim uses the simulator ${\mathrm{Sim}}^{\prime }$ of the underlying ZK-PCP system $\left(P^{\prime },V^{\prime }\right)$ to simulate the symbol $\pi_{i}^{\prime }$. To continue with the simulation, Sim must now generate an encoding of $\pi_{i}^{\prime }$ which is (1) consistent with the bits already probed from the *i*’th segment; and (2) is distributed as a random encoding of $\pi_{i}^{\prime }$ subject to (1). For this, we need the underlying probing-resilient encoding to be *equivocal*—allowing one to efficiently sample from this distribution. Such encodings are called *Reconstructable Probabilistic Encodings (RPEs)* [34,35].

**Figure 1:**
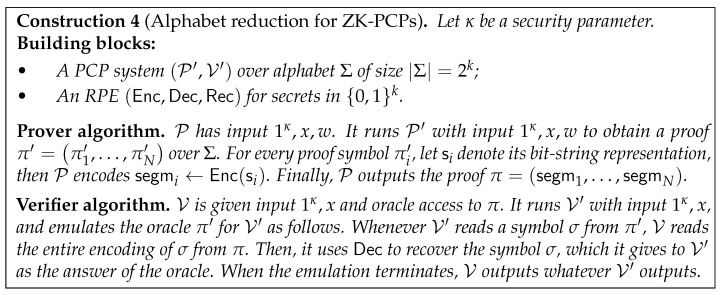
Alphabet Reduction For ZK-PCPs [13].

**Putting It Together.** We are now ready to describe the full alphabet reduction (see Section 3.2, and Figure 1 in particular) that transforms a ZK-PCP variant $\left(P^{\prime },V^{\prime }\right)$ over alphabet Σ into a ZK-PCP $\left(P,V\right)$ (over bits). The reduction employs an RPE (see Section 3.1). For proof generation, the prover P first runs $P^{\prime }$ to generate a proof $\pi^{\prime }=\left(\pi_{1}^{\prime },\ldots ,\pi_{N}^{\prime }\right)$ over Σ. Then, it replaces each proof symbol $\pi_{i}^{\prime }$ with its binary representation $s_{i}$, and uses the RPE to encode $s_{i}$ into a segment ${\mathrm{segm}}_{i}$. P outputs the proof $\pi =\left({\mathrm{segm}}_{1},\ldots ,{\mathrm{segm}}_{N}\right)$. The verifier V, given oracle access to π, verifies the proof by emulating $V^{\prime }$. Whenever $V^{\prime }$ queries a symbol $\pi_{q}^{\prime }$ of $\pi^{\prime }$, V queries ${\mathrm{segm}}_{q}$ from π, RPE-decodes it to obtain the binary representation $s_{q}$ of a symbol $\sigma_{q}\in \Sigma$, and provides $\sigma_{q}$ to $V^{\prime }$ as the answer of the oracle. When the emulation ends, V outputs whatever $V^{\prime }$ outputs. In the following sections, we describe the RPE building block (Section 3.1), and analyze the resultant ZK-PCP scheme (Section 3.2).

### 3.1. Main Building Block: Reconstructable Probabilistic Encdoings (RPEs)

The main building block of the ZK-preserving alphabet reduction is an encoding scheme with equivocation properties called *Reconstructable Probabilistic Encoding (RPE)* [34,35,36,37]. In this section, we formally define these objects.

Codes, or encoding schemes, are extensively used in computer science, the most notable example being Error-Correcting Codes (ECCs), which are used to guarantee that the data can still be recovered even if faults occur (i.e., some of the symbols of the data are erased or corrupted). A code consists of an encoding procedure Enc which maps a message to a codeword, and a decoding procedure Dec which decodes the message from a (possibly corrupted) codeword. In the context of error-correction, Enc,Dec are usually deterministic. Most ECCs are linear codes, where the code is defined by a *generator matrix*, and encoding simply multiplies the generator matrix with the message. The main parameters of interest for such codes are: (1) the *rate* of the code—the ratio between the length of the encoding (also known as a *codeword*) and the length of the original message; (2) its distance—namely the minimal distance between a pair of codewords, which is the number of coordinates in which they differ; and (3) the alphabet size (where most ECCs are binary). There are numerous extensions and generalizations of ECCs that guarantee additional properties beyond error correction.

We will be interested in a generalized notion of an ECC which also guarantees *probing-resilience* in the sense that few codeword symbols reveal no information (in an information–theoretic sense) on the encoded message. Of course, such a guarantee cannot be satisfied if encoding is deterministic. Probing-resilient encodings therefore allow for a *randomized* encoding procedure, where each message has a subset of codewords to which it can be mapped, and encoding chooses one of them at random. It is fairly simple to obtain such a leakage-resilient encoding from a linear code, as long as its generator matrix has a “good” structure. (See, e.g., [38] for a description of the needed properties and how encoding works.).

As explained above, probing-resilience alone is insufficient to guarantee ZK against *adaptive* malicious verifiers. Instead, we rely on a stronger *equivocation* property which guarantees that as long as the probing threshold τ had not been violated, the probed bits can be efficiently “explained” as the bits in an encoding of *any* arbitrary message msg. Intuitively, an RPE is an encoding scheme (se defined in Section 2) which is probing-resilient, and is additionally associated with a *resampling/reconstruction* algorithm Rec that can “explain” the probed bits. Formally:

**Definition** **9** (Reconstructable Probabilistic Encoding (RPE)). *Let k,n,ℓ∈N. A*$\left(k,n,\ell \right)$-Reconstructable Probabilistic Encoding (RPE)*is a triple $\left(\mathrm{Enc},\mathrm{Dec},\mathrm{Rec}\right)$ where Enc,Rec are PPT algorithms, and Dec is a (deterministic) polynomial-time algorithm, that satisfy the following.*

- **Syntax.***Enc on input a secret $x\in {\left\{0,1\right\}}^{k}$ outputs a codeword $c\in {\left\{0,1\right\}}^{n}$. Dec on input $c\in {\left\{0,1\right\}}^{n}$ outputs $x\in {\left\{0,1\right\}}^{k}$ or a special error symbol* ⊥*. Rec on input a secret x, a set $I\subset \left[n\right]$ of size $\left|I\right|\leq \ell$, and ℓ bits ${\left(c_{i}\right)}_{i\in I}$, outputs $c^{\prime }\in {\left\{0,1\right\}}^{n}$;*
- **Correctness.***There exists a t≥0 such that the following holds for every $x\in {\left\{0,1\right\}}^{k}$, and every $c\in {\left\{0,1\right\}}^{n}$: if there exists $c_{x}\in \mathrm{Supp}\left(\mathrm{Enc}\left(x\right)\right)$ such that $\mathrm{Ham}\left(c,c_{x}\right)\leq t$ then $\mathrm{Dec}\left(c\right)=x$, otherwise Dec outputs* ⊥;
- ***ℓ*-Secrecy (of partial views).***For every pair of secrets $x,x^{\prime }$, and any subset $I\subseteq \left[n\right]$ such that $\left|I\right|\leq \ell$, $\mathrm{Enc}\left(x\right){{|}_{I}\equiv \mathrm{Enc}\left(x^{\prime }\right)|}_{I}$;*
- ***ℓ*-Reconstruction (from partial views).***For any secret x, any subset $I\subseteq \left[n\right]$ of size $\left|I\right|\leq \ell$, and any set ${\left(c_{i}^{\prime }\right)}_{i\in I}$ of bits, $\mathrm{Rec}\left(x,I,{\left(c_{i}^{\prime }\right)}_{i\in I}\right)$ is distributed identically to an encoding $c\in \mathrm{Supp}\left(\mathrm{Enc}\left(x\right)\right)$ that is random subject to being consistent with ${\left(c_{i}^{\prime }\right)}_{i\in I}$.*

A few remarks are in order. First, our constructions can make do with a relaxed RPE notion in which the secrecy and reconstruction properties hold statistically with statistical distance ϵ. (In this case, the resultant ZK-PCP will have statistical ZK with a statistical error of roughly N·ϵ, where *N* denotes the proof length, see [13] for details.) Second, while we define RPEs for a single message length *k*, the notion naturally generalizes to *families* of codes such that for every k∈N there exists a code the codewords of which have length $n=n\left(k\right)$; and there exists a uniform algorithm that given $1^{k}$ as input, generates the encoding, decoding and resampling procedures for message length *k*. We will only consider (efficiently encodable and decodable) *families* of codes in this work. For simplicity and clarity of the definitions, we do not explicitly refer to a family of codes, but *k* should be understood as a general input length parameter. (This is standard in the literature.) Finally, we note that non-explicit constructions of RPEs follow easily from the existence of linear error-correcting codes with sufficiently “good” parameters, which are satisfied by random linear codes (see, e.g., [14]). Indeed, such codes posses the secrecy property of RPEs, which guarantees that for every subset of *ℓ* codeword symbols, the resultant system of linear equations has a solution for *any* possible secret. This gives an efficient reconstructor Rec. We note that the final ZK-PCP construction will use an *explicit* RPE construction due to [37,39].

### 3.2. The ZK-PCP Construction

We now describe the alphabet reduction for ZK-PCPs, which uses RPEs to transform a ZK-PCP variant over a large alphabet to a ZK-PCP (over bits).

Construction 4 transforms a PCP system over a large alphabet into a PCP (over bits). The following theorem of ([40], Theorem 9) states that if the underlying PCP system is ZK, and the RPE is secure, then the resultant scheme is also ZK.

**Theorem** **5** (ZK-PCPs from LR encodings [13]). *Assume Construction 4 is instantiated with:*

- *A $\left(q^{*},\epsilon \right)$-ZK-PCP $\left(P^{\prime },V^{\prime }\right)$ over alphabet Σ for a language L;*
- *A $\left(k,n,\ell \right)$-RPE $\left(\mathrm{Enc},\mathrm{Dec},\mathrm{Rec}\right)$.*

*Then, Construction 4 is a $\left(\left(q^{*}+1\right)\cdot \left(\ell +1\right)-1,\epsilon \right)$-ZK-PCP for L.*

*Moreover, the transformation preserves the soundness and completeness of $\left(P^{\prime },V^{\prime }\right)$. Furthermore, if $\left(P^{\prime },V^{\prime }\right)$ has proofs of length N that can be verified non-adaptively with $q^{\prime }$ queries, then Construction 4 has proofs of length N·n that can be verified non-adaptively with $q=q^{\prime }\cdot n$ queries.*

**Proof.**  **Completeness** follows directly from a combination of the completeness of $\left(P^{\prime },V^{\prime }\right)$ and the correctness of the RPE (which guarantees that V perfectly emulates the proof oracle for $V^{\prime }$). The claim regarding *q* follows directly from the construction.

**Soundness.** Let $x^{*}\notin L$, and let $\pi^{*}$ be a purported proof oracle for V. We show that V rejects $x^{*}$ with the same probability as $V^{\prime }$. We partition $\pi^{*}$ into *N* length-*n* segments $\pi^{*,1}\cdots \pi^{*,N}$, where the *i*’th segment $\pi^{*,i}$ contains the bits in locations $\left(i-1\right)n+1,\ldots ,i\cdot n$. (Notice that the *i*’th segment in an *honestly-generated proof* would contain the RPE-encoding of the *i*’th symbol in a proof over Σ.) Then the correctness of the RPE implies that for every 1≤i≤N there exists a $\sigma_{i}\in \Sigma$ such that $\mathrm{Dec}\left(\pi^{*,i}\right)=\sigma_{i}$ (indeed, if $\mathrm{Dec}\left(\pi^{*,i}\right)=⊥$ then we set $\sigma_{i}$ to some arbitrary symbol in Σ). Let $\pi^{*\prime }=\left(\sigma_{1},\ldots ,\sigma_{N}\right)\in \Sigma^{N}$, and notice that when V has oracle access to $\pi^{*}$, it emulates $V^{\prime }$ with oracle access to $\pi^{*\prime }$. The soundness of $\left(P^{\prime },V^{\prime }\right)$ therefore guarantees that $V^{\prime }$ (and consequently also V) accepts with probability ϵ.

**Zero Knowledge.** Let $q^{**}=\left(q^{*}+1\right)\cdot \left(\ell +1\right)-1$, and let $V^{*}$ be a (possibly malicious and adaptive) $q^{**}$-query bounded verifier. For simplicity of the description, we assume $V^{*}$ makes its queries one at a time, and never repeats queries (this is without loss of generality). We describe a simulator Sim for $V^{*}$, which relies on the simulator ${\mathrm{Sim}}^{\prime }$ of the underlying ZK-PCP system $\left(P^{\prime },V^{\prime }\right)$, and emulates a proof oracle $\pi^{*}$ for $V^{*}$ as follows:

1. $\pi^{*}\in {\left\{0,1\right\}}^{N\cdot n}$ is the concatenation of *N* segments ${\mathrm{segm}}_{1},\ldots ,{\mathrm{segm}}_{N}\in {\left\{0,1\right\}}^{n}$, which Sim initializes as random and independent RPE-encoding of $0^{k}$, by computing ${\mathrm{segm}}_{i}\leftarrow \mathrm{Enc}\left(0^{k}\right)$.
  Sim additionally maintains *N* counters ${\mathrm{Cnt}}_{1},\ldots ,{\mathrm{Cnt}}_{N}$, initialize to 0, and *N* sets $I_{1},\ldots ,I_{N}$, initialized to *∅*;
2. Sim answers each oracle query *Q* of $V^{*}$ as follows. Assume that *Q* is a query to the *j*’th bit of the *i*’th segment (meaning *Q* queries the $\left(i-1\right)n+j$’th bit of the proof);
  (a) If ${\mathrm{Cnt}}_{i}<\ell$, or ${\mathrm{Cnt}}_{i}\geq \ell +1$, then Sim answers with the *j*’th bit of ${\mathrm{segm}}_{i}$, increases ${\mathrm{Cnt}}_{i}$ by 1, and adds *j* to $I_{i}$;
  (b) Otherwise, ${\mathrm{Cnt}}_{i}=\ell$, meaning $V^{*}$ has already queried *ℓ* bits from the encoding in the *i*’th segment. In this case, Sim uses ${\mathrm{Sim}}^{\prime }$ to simulate the *i*’th symbol $\sigma_{i}$ of a proof of the underlying ZK-PCP system. Then, Sim resamples an encoding of $\sigma_{i}$ by computing ${\mathrm{segm}}_{i}^{\prime }\leftarrow \mathrm{Rec}\left(\sigma_{i},I_{i},\left({\mathrm{segm}}_{i}\right){|}_{I_{i}}\right)$ (this resamples a fresh encoding of $\sigma_{i}$ consistently with the oracle answers already simulated), sets ${\mathrm{segm}}_{i}:={\mathrm{segm}}_{i}^{\prime }$, and provides the *j*’th bit of ${\mathrm{segm}}_{i}$ as the answer of the oracle. Finally, it increases ${\mathrm{Cnt}}_{i}$ by 1.

We now prove that the simulated and real views of $V^{*}$ are ϵ-statistically close, using a hybrid argument.

$H_{0}$: This is the view of $V^{*}$ in the simulation described above;
$H_{1}^{0}$: $H_{1}^{0}$ is obtained from $H_{0}$ by replacing the simulated answers of ${\mathrm{Sim}}^{\prime }$ with the actual proof symbols of a proof $\pi^{\prime }$ honestly generated by $P^{\prime }$.
  *Then $H_{0}$ and $H_{1}^{0}$ are ϵ-statistically close by the $\left(q^{*},\epsilon \right)$-ZK of $\left(P^{\prime },V^{\prime }\right)$.*
  Indeed, since ${\mathrm{Sim}}^{\prime }$ is used to simulate the *i*’th proof symbol only when ${\mathrm{Cnt}}_{i}=\ell$, i.e., only on the ℓ+1 query to the *i*’th segment, and since $q^{**}=\left(q^{*}+1\right)\left(\ell +1\right)-1$, ${\mathrm{Sim}}^{\prime }$ is only used to simulate at most $q^{*}$ symbols, and so the (adaptive) ZK of $\left(P,V\right)$ implies that the simulated answers are ϵ-statistically close to the corresponding symbols in a real proof $\pi^{\prime }$.
$H_{1}^{i},1\leq i\leq N$: $H_{1}^{i}$ is obtained from $H_{1}^{i-1}$ by replacing the simulated answers of Sim with the actual bits in the *i*’th section of the proof π. (In particular, these are bits in random RPE-encodings of $\pi_{1}^{\prime },\ldots ,\pi_{N}^{\prime }$.)
  *Then $H_{1}^{i-1}$ and $H_{1}^{i}$ are identically distributed by the ℓ-secrecy or ℓ-reconstruction of the RPE.*
  To see why this holds, we consider two cases depending on whether or not $V^{*}$ made more than *ℓ* queries to the *i*’th segment. If $V^{*}$ made at most *ℓ* queries to the *i*’th segment, then all queries in $H_{1}^{i-1}$ were answered according to an RPE-encoding of $0^{k}$, whereas all queries in $H_{1}^{i}$ were answered according to an RPE-encoding of (the binary representation of) $\pi_{i}^{\prime }$. The bits queried in the *i*’th segment (and consequently, also the entire hybrids) are therefore identically distributed by the *ℓ*-secrecy of the RPE.
  If, on the other hand, $V^{*}$ made *more thanℓ* queries to the *i*’th segment, then the first *ℓ* queries in $H_{1}^{i-1}$ were answered according to an RPE-encoding of $0^{k}$, and the remaining queries were answered using a resampled encoding of (the binary representation of) $\pi_{i}^{\prime }$, resampled consistently with the answers to the first *ℓ* queries; whereas in $H_{1}^{i}$ all queries to the *i*’th segment were answered according to a random encoding of (the binary representation of) $\pi_{i}^{\prime }$. In this case, the distributions are identically distributed by the *ℓ*-reconstruction of the RPE.

We conclude the proof by noting that $H_{1}^{N}$ is distributed identically to the real view of $V^{*}$. □

**Remark** **5** (ZK-PCPs from weaker primitives). *We note that if one only requires that ZK hold against malicious* non-adaptive *verifiers, then it suffices for the underlying ZK-PCP system over Σ to have ZK against non-adaptive verifiers. Additionally, the reduction can be instantiated with an RPE in which secrecy and reconstruction hold* statistically *with some error $\epsilon^{\prime }$, in which case the overall simulation error will be $\epsilon +\epsilon^{\prime }\left(N-q^{*}\right)$. We refer the interested reader to [40] for a proof of these claims.*

### 3.3. ZK-PCPs with Square-Root Gap

In this section we describe the $\sqrt{q^{*}}$-query $q^{*}$-ZK-PCPs of [13] (i.e., the system has $q^{*}$-ZK with an honest verifier that makes $\sqrt{q^{*}}$ queries), which are obtained by appropriately instantiating the building blocks of Construction 4. These are the only known PCPs to date that have full-fledged ZK with a non-adaptive honest verifier. We first describe how we instantiate the building blocks.

**The Building Blocks.** Hazay et al. [13] instantiate Construction 4 with a ZK-PCP of [15] (using an improved soundness analysis given in [13]), and an RPE based on linear codes.

More specifically, the ZK-PCP over Σ is obtained using the “MPC-in-the-head” technique. It has perfect ZK against malicious verifiers querying at most a constant fraction of proof symbols (for an a-priori bounded constant), where the honest verifier obtains a negligible soundness error by non-adaptively querying only a square-root of the proof symbols. We note that the original soundness analysis of [15] required the honest verifier to make as many queries as a malicious verifier, but this analysis was recently improved by [13].

**Theorem** **6** (Non-adaptive ZK-PCPs over large alphabets with $\sqrt{Q}$-gap, implicit in [15]). *For any L∈NP, any Q≥3, and any input length n, there exists an alphabet Σ of size $\left|\Sigma \right|=2^{\mathrm{poly}\left(n,\log Q\right)}$ for which there exists a ZK-PCP for L over Σ, with $\mathrm{negl}\left(Q\right)$ soundness error with a non-adaptive honest verifier that makes $\log Q\cdot \sqrt{Q}$ queries, proofs of length Q, and perfect $\Omega \left(Q\right)$-ZK.*

The RPE that [13] uses is obtained by applying a general observation of [37,39]—that the existence of linear codes implies the existence of RPEs—to the linear codes of Decatur [41]. Specifically, Ball et al. ([37], Lemma 2) prove the following, where a code $C\subseteq {\left\{0,1\right\}}^{n}$ is linear if its encoding procedure simply multiplies the input with a public generator matrix, and the distance of the code is $\min_{c\in C}\mathrm{Ham}\left(c,0^{n}\right)$ (i.e., the minimal weight of a non-zero codeword):

**Lemma** **1** (RPEs from linear error-correcting codes [37]). *If there exists a linear error-correcting code $C\subseteq {\left\{0,1\right\}}^{n}$ with messages in ${\left\{0,1\right\}}^{k}$ and distance d, then there exists a $\left(k,n,d-1\right)$-RPE.*

To obtain an RPE with good parameters, we apply Lemma 1 to any explicit family of linear codes with constant rate and constant relative distance. For example, we can use the codes of ([41], Theorem 2.1), which already posses secrecy from partial views. (We note that the construction of [41] relies on Toeplitz matrices of logarithmic size which generate codes with good parameters. Such a matrix can be efficiently found by traversing all these matrices by some pre-defined order, and using the first matrix satisfying the desired properties.) In particular, we have

**Corollary** **1** (RPEs). *For every message length k∈N, there exists a $\left(k,O\left(k\right),\Omega \left(k\right)\right)$-RPE.*

**A ZK-PCP with Square-Root Query Gap.** With these building blocks in place, we are ready to prove Theorem 3.

**Proof**  (Proof of Theorem 3). We instantiate Theorem 5 with the ZK-PCP system of Theorem 6 and the RPE of Corollary 1. We assume without loss of generality that $q^{*}\geq n$ (where *n* is the input length). Since we set *Q* below to be polynomially-related to $q^{*}$ (and consequently also to *n*), there exists a constant *c* such that the PCP of Theorem 6 is over an alphabet of size $2^{Q^{c}}$. Let $\alpha =1/\left(c+1\right)$, then we instantiate Theorem 6 with $Q:={\left(q^{*}\right)}^{\alpha }$, and set $k=Q^{c}$ in Corollary 1. Then Theorem 5 guarantees that the resultant ZK-PCP has proofs of length $Q\cdot O\left(Q^{c}\right)=O\left(Q^{c+1}\right)={\left(q^{*}\right)}^{\alpha \cdot \left(c+1\right)}=O\left(q^{*}\right)$ with perfect ZK against (possibly malicious and adaptive) verifiers making $\Omega \left(Q\right)\cdot \Omega \left(Q^{c}\right)=\Omega \left(q^{*}\right)$ queries, and a $\mathrm{negl}\left(Q\right)=\mathrm{negl}\left(q^{*}\right)$ soundness error with a non-adaptive honest verifier the query complexity of which is $\log Q\cdot \sqrt{Q}\cdot O\left(Q^{c}\right)=\tilde{O}\left(Q^{c+1/2}\right)=\tilde{O}\left({\left(q^{*}\right)}^{\left(c+1/2\right)/\left(c+1\right)}\right)$. The theorem now follows for any ϵ which is larger than $\left(c+1/2\right)/\left(c+1\right)$ (and for a sufficiently large $q^{*}$). □

Theorem 3 allows one to choose the ZK query bound $q^{*}$. Hazay et al. [13] also give an alternative formulation of Theorem 3, in which the square-root query gap obtained by the ZK-PCP system is more clearly manifested.

**Corollary** **2** (ZK-PCP with $\sqrt{n}$ query gap, Corollary 10 of [40]). *There exists a constant c>0 such that there exists ZK-PCP with perfect $\Omega \left(n^{c+1}\right)$-ZK, and $\mathrm{negl}\left(n\right)$ soundness error with an honest verifier that non-adaptively queries $\tilde{O}\left(n^{c+1/2}\right)$ proof bits, where n denotes the input length.*

**Proof.**  We instantiate Theorem 5 with the ZK-PCP system of Theorem 6 and the RPE of Corollary 1. Setting Q=n in Theorem 6, let *c* be a constant such that the PCP of theorem 5 is over an alphabet of size $n^{c}$. We set $k=n^{c}$ in Corollary 1. Then Theorem 5 guarantees that the resultant ZK-PCP has proofs of length $n\cdot O\left(n^{c}\right)=O\left(n^{c+1}\right)$ with perfect ZK against (possibly malicious and adaptive) verifiers making $\Omega \left(n\right)\cdot \Omega \left(n^{c}\right)=\Omega \left(n^{c+1}\right)$ queries, and a $\mathrm{negl}\left(n\right)$ soundness error with a non-adaptive honest verifier the query complexity of which is $\log n\cdot \sqrt{n}\cdot O\left(n^{c}\right)=\tilde{O}\left(n^{c+1/2}\right)$. □

## 4. The ZK-PCPs of Ishai et al.: ZK from LR Circuits

The main result of this section is a construction of witness-indistinguishable PCPs for NP with a non-adaptive honest verifier and an exponential query gap (between the query complexity of the honest and malicious verifiers), which was given in [8]:

**Theorem** **7** (WI-PCPs for NP, formal statement of Informal Theorem 2). *Let n∈N be an input length parameter. For any query bound $q^{*}=\mathrm{poly}\left(n\right)$ there exists a non-adaptive $\mathrm{poly}\left(\log q^{*},\kappa \right)$-query $\left(q^{*},\mathrm{negl}\left(q^{*}\right)\right)$-WI-PCP for NP with $\mathrm{negl}\left(\kappa \right)$ soundness error, where κ is a statistical security parameter.*

The WI-PCP system is constructed from leakage-resilient *circuits*. Historically, this construction was presented before the ZK-PCPs of Section 3, and was the first to show a connection between ZK-PCPs and leakage resilience. Moreover, it was the first construction of PCPs with (relaxed) ZK against *malicious* verifiers with *non-adaptive honest* verification. Compared to the ZK-PCPs of Section 3, the scheme we describe in this section has the advantage of obtaining an *exponential* query gap: the scheme is ZK against $q^{*}$-query bounded verifiers, but the honest verifier only needs to query $\mathrm{polylog}\left(q^{*}\right)$ proof bits to verify the proof (setting $\kappa =\mathrm{polylog}\left(q^{*}\right)$). However, the PCP system described in this section obtains a weaker form of ZK called *Witness Indistinguishability (WI)* in which the ZK simulator is not guaranteed to be efficient. The construction of Section 3 has the added feature of being simpler.

The main building-block in the transformation of [8] are leakage-resilient *circuits*—a stronger primitive than the leakage-resilient *encodings* used by [13]. Indeed, leakage-resilient encodings only protect *information*, whereas leakage-resilient *circuits* protect *computations*. Thus, while Hazay et al. [13] could only use leakage-resilient encodings to protect the proof *once it was already generated*, Ishai et al. [8] employ leakage-resilient circuits to protect *proof generation itself*. When used in the context of proof generation, these leakage-resilient circuits in effect amplify leakage resilience: from a relatively low leakage bound on the witness, to a much larger leakage bound on the entire computation. This amplification results in an exponential query gap, which eluded the ZK-PCPs of Section 3.

**High-Level Idea: Leakage-Resilient Proof Generation.** The goal of the prover P is to convince the verifier V that x∈L, where P has a corresponding witness *w*. Recall from Section 1.1.2 that one way of doing so is to emulate the verification circuit *C* of the corresponding NP-relation $R_{L}$ on $\left(x,w\right)$, where V then checks that this computation was performed correctly. Indeed, without loss of generality we can assume that $R_{L}$ has a canonical form in which the witness consists of the entire wire values of *C*. In particular, in a PCP system the prover would generate the PCP from the entire wire values of *C*, which we denote by $\left[C,x,w\right]$. While this might blatantly violate ZK (e.g., *w* itself is part of $\left[C,x,w\right]$, and the PCP might explicitly contain these wire values), the main observation of [8] is that by querying few proof bits, V is leaking on a *computation*—namely, the evaluation of *C* on $\left(x,w\right)$. Therefore, one can use leakage-resilient circuits to guarantee that this leakage gives V no information on the witness *w* (i.e., *C*’s input).

Concretely, one can first replace *C* with a *leakage-resilient version*$\hat{C}$, then have V check the computation performed in $\hat{C}$. If V were reading directly from the wire values of $\hat{C}$, then we would need $\hat{C}$ to resist probing leakage (similar to Section 3). However, V is actually probing bits in the *PCP π*, which was generated by applying the prover algorithm to the wire values of $\hat{C}$. Therefore, V obtains more evolved forms of leakage on these wire values, and we thus need leakage resilience against a wider class of potential leakage functions. Still, V cannot obtain any leakage that it wants on the wire values of $\hat{C}$, but rather it is restricted to whatever the prover computes on these wire values during proof generation. In particular, by restricting the types of functions the prover applies to the wire values of $\hat{C}$, we can “control” the type of information which *even a malicious* verifier $V^{*}$ obtains on the witness. (Indeed, $V^{*}$ can deviate from an honest verification strategy by choosing to read different—and a larger number of—proof bits than the honest verifier, but cannot affect the proofs generation itself.) This gives a general blueprint for transforming standard PCPs into *ZK*-PCPs: the prover and verifier both hard-wire *x* into the verification circuit *C*, to obtain a circuit $C_{x}$. Then, they generate the LR-version of it $\hat{C_{x}}$, which operates on encoded inputs (see Section 4.1 below). Then, P randomly encodes the witness *w* as $\hat{w}$, and generates the PCP from the wire values $\left[\hat{C},\hat{w}\right]$. Finally, the verifier V runs the PCP verifier to verify the proof.

**Achieving Soundness.** While (for an appropriate choice of the family of leakage functions) the blueprint described above would indeed yield ZK proofs, they would not be sound. The reason is that soundness of the original PCP relied on the fact that $C\left(x,\cdot \right)$ is satisfiable only if x∈L, i.e., there exists a corresponding witness *w* such that $\left(x,w\right)\in R_{L}$. This, however, is not preserved in the *leakage-resilient* version $\hat{C_{x}}$ of $C\left(x,\cdot \right)$. In fact, in many (if not all) existing constructions, $\hat{C_{x}}$ is *necessarily* satisfiable—leakage resilience relies on this fact! To understand why this is the case, and how we can still achieve soundness, we need to first take a closer look at how these LR constructions work.

### 4.1. Leakage-Resilient Circuit Compilers (LRCCs)

In this section we describe the main building block used in the ZK-PCPs of this section: Leakage-Resilient Circuits Compilers (LRCCs). Compared to the LR encodings used in Section 3, LRCCs offer a stronger LR guarantee: they protect *computation* rather than *information*. More specifically, LRCCs are compilers which transform a given circuit *C* into a functionally-equivalent circuit $\hat{C}$, which is leakage resilient in the sense that leakage on the wire values of $\hat{C}$ reveals no information on its input. Of course, this cannot be obtained if the input of *C* is given to $\hat{C}$ in the clear, since then leakage on the wire values reveals information about the input. Instead, the LRCC is associated with a (LR) encoding scheme, which is used to encode the inputs to (and intermediate values in the computation of) the LR circuit $\hat{C}$.

We first define the circuit model which we use, then define circuit compilers, and finally define leakage-resilient circuit compilers.

**Circuit Model.** An (arithmetic) circuit *C* over the field F and the set $X=\left\{x_{1},\ldots ,x_{n}\right\}$ of variables is a directed acyclic graph the vertices of which are called *gates* and the edges of which are called *wires*, and are labeled with functions over *X*. Every gate in *C* of in-degree 0 has out-degree 1 and is either an *input* gate labeled by a variable from *X*; or a *constant* gate ${\mathrm{const}}_{\alpha }$ labeled by a constant α∈F. Gates of in-degree 2 and out-degree 1 are labeled by one of the operations +,−,×, i.e., addition, subtraction, and multiplication over F. (Jumping ahead, we will use an LRCC of [42], which employs additional gates, e.g., to duplicate values in the circuit. Since we do not explicitly use these additional gates, we omit their description to simplify the presentation.) The *size* $\left|C\right|$ of a circuit *C* is the sum of the number of wires, input gates, and output gates, in *C*. The *depth* of *C* is the number of gates on the longest path from inputs to outputs. We also consider *Boolean* circuits, with ∧,∨ gates (replacing the +,−,× gates of arithmetic circuits), ${\mathrm{const}}_{0}$ and ${\mathrm{const}}_{1}$ gates, and ¬ gates with in- and out-degree 1.

We define several circuit complexity classes, which restrict the size and depth of Boolean and arithmetic circuits. Specifically,

**Notation** **4.** *$SHALLOW\left(d,s\right)$ denotes the class of all depth-d, size-s arithmetic circuits over F. Similarly, $BOOL\left(d,s\right)$ denotes the class of all depth-d, size-s Boolean circuits. Somewhat abusing notation, we use the same notations to denote the* families of functions *computable by circuits in the respective class of circuits. $AC^{0}$ denotes all constant-depth and polynomial-sized Boolean circuits over* unbounded fan-in and fan-out *(i.e., in-degree and out-degree) $\wedge ,\vee ,\neg ,{\mathrm{const}}_{0}$ and ${\mathrm{const}}_{1}$ gates.*

**Definition** **10** (Satisfiable Circuits). *A circuit $C:F^{n}\to F$ is* satisfiable *if there exists an $x\in F^{n}$ such that*

$$C\left(x\right)=\left\{\begin{array}{cc} 1 & C\;is\;Boolean\\ 0 & C\;is\;arithmetic \end{array}\right.$$

**Circuit Compilers.** We define the notion of a circuit compiler. Informally, it consists of an encoding scheme and a compiler algorithm, that compiles a given circuit *C* into a circuit $\hat{C}$ that emulates the operation of *C* over encoded inputs. Formally,

**Definition** **11** (Circuit compiler over F). *A circuit compiler over F is a pair $\left(\mathrm{Comp},\left(\mathrm{Enc},\mathrm{Dec}\right)\right)$ such that the following holds:*

- **Syntax:**
  - *$\left(\mathrm{Enc},\mathrm{Dec}\right)$ is an encoding scheme, where Enc is a PPT algorithm that on input a vector $x\in F^{n}$, and an additional length parameter $1^{\mathrm{len}}$ (which is used to determine the amount of random masks needed to protect the computation from leakage; see Definition 13), outputs a vector $\hat{x}$, and Dec is a polynomial-time algorithm; We assume that $\hat{x}\in F^{\hat{n}}$ for some $\hat{n}=\hat{n}\left(n,\mathrm{len}\right)$.*
  - *Comp is a polynomial-time algorithm that on input an arithmetic circuit C over F outputs an arithmetic circuit $\hat{C}$;*
- **correctness:** *For any arithmetic circuit C, and any input x for C, we have $\mathrm{Pr}\left[\hat{C}\left(\hat{x}\right)=C\left(x\right)\right]=1$, where $\hat{x}\leftarrow \mathrm{Enc}\left(x,1^{\left|C\right|}\right)$.*

As discussed above, we will need circuit compilers that are also “sound” in the sense that the compiled circuit $\hat{C}$ is satisfiable only if the original circuit *C* is satisfiable. We stress that this property should hold even when the inputs of $\hat{C}$ are *not* valid encodings according to Enc.

**Definition** **12** (SAT-respecting circuit compiler). *A circuit compiler $\left(\mathrm{Comp},\left(\mathrm{Enc},\mathrm{Dec}\right)\right)$ is* SAT-respecting *if for every circuit $C:F^{n}\to F$, if $\hat{C}=\mathrm{Comp}\left(C\right)$ is satisfiable then C is satisfiable. That is,*

- *For arithmetic C: if there exists an ${\hat{x}}^{*}\in F^{\hat{n}}$ such that $\hat{C}\left({\hat{x}}^{*}\right)=0$, then there exists an $x\in F^{n}$ such that $C\left(x\right)=0$;*
- *For Boolean C: if there exists an ${\hat{x}}^{*}\in {\left\{0,1\right\}}^{\hat{n}}$ such that $\hat{C}\left({\hat{x}}^{*}\right)=1$, then there exists an $x\in {\left\{0,1\right\}}^{n}$ such that $C\left(x\right)=1$.*

**Leakage-Resilient Circuit Compilers.** We now define *Leakage-Resilient* circuit compilers. An LRCC is associated with a class LEAK of leakage functions, and guarantees that when the input to $\hat{C}$ is properly encoded, then leakage from LEAK on the wire values of $\hat{C}$ reveals no information about the input and internal computations, except for the output of $\hat{C}$. This is formalized by requiring that the wire values are distributed statistically close for every pair of inputs $x,x^{\prime }$ such that $C\left(x\right)=C\left(x^{\prime }\right)$. We first set some notation.

**Notation** **5.** *For a Circuit C, a leakage function $\ell :F^{\left|C\right|}\to F^{m}$ for some m∈N, and an input x for C, $\left[C,x\right]$ denotes the wire values of C when evaluated on x, and $\ell \left[C,x\right]$ denotes the output of ℓ on $\left[C,x\right]$.*

**Definition** **13** (LRCC). *Let F be a finite field, LEAK be a function class, $S\left(n\right):N\to N$ be a size function, and $\epsilon \left(n\right):N\to R^{+}$. A circuit compiler $\left(\mathrm{Comp},\left(\mathrm{Enc},\mathrm{Dec}\right)\right)$ is*$\left(LEAK,\epsilon \left(n\right),S\left(n\right)\right)$-leakage resilient *if for all sufficiently large n’s, every arithmetic circuit $C:F^{n}\to F^{m}$ (for some m) of size $\left|C\right|\leq S\left(n\right)$, every ℓ∈LEAK of input length $\left|\hat{C}\right|$, and every $x,x^{\prime }\in F^{n}$ such that $C\left(x\right)=C\left(x^{\prime }\right)$, we have*

$$\mathrm{SD}\left(\ell \left[\hat{C},\hat{x}\right],\ell \left[\hat{C},\hat{x^{\prime }}\right]\right)\leq \epsilon \left(n\right)$$

*where $\hat{x}\leftarrow \mathrm{Enc}\left(x,1^{\left|C\right|}\right)$ and $\hat{x^{\prime }}\leftarrow \mathrm{Enc}\left(x^{\prime },1^{\left|C\right|}\right)$.*

*LRCCs for Boolean circuits are defined similarly.*

A few remarks are in order. First, we note that the LR guarantee of Definition 13 is a relaxed version of the standard notion. Specifically, while Definition 13 only guarantees *indistinguishability*—namely, leakage functions cannot distinguish between the wire values of $\hat{C}$ when evaluated on two different inputs (so long as *C* has the same output on both), the standard definition (e.g., in [18,42,43,44]) is *simulation-based*. That is, the standard definition requires the existence of a *PPT* simulator which, for every circuit *C*, every input *x* for *C*, and any leakage function ℓ∈LEAK, can simulate the leakage on the wire values of $\hat{C}$ given only the description of *C* and its output $C\left(x\right)$. To see why this guarantee is stronger, notice that if for some input *x* there exists *no $x^{\prime }$* such that $C\left(x\right)=C\left(x^{\prime }\right)$ then Definition 13 provides no secrecy guarantee for *x*. Moreover, Definition 13 is equivalent to a slightly modified version of the standard definition—specifically, in which there exists a simulator as specified above, but it is *not* guaranteed to be efficient. Indeed, given $C,C\left(x\right)$ the simulator could find on its own an $x^{\prime }$ such that $C\left(x\right)=C\left(x^{\prime }\right)$, then simulate the leakage by computing $\ell \left[\hat{C},\hat{x^{\prime }}\right]$. We focus on the relaxed version because it captures the security guarantee which we achieve (in fact, Ishai et al. [8] give strong indications that SAT-respecting LRCCs for “useful” leakage, with the stronger LR guarantee with *efficient* simulation, do not exist—see Section 1.2).

Second, we note that for simplicity, the error in Definitions 12 and 13 depends (only) on the input length *n*. This can be naturally extended such that the error depends also on a security parameter κ, which is given as input to Comp.

**Gadget-Based LRCCs.** A leading technique in constructing LRCCs — which is the one employed in all LRCCs described in this work — is *gadget-based*. Such constructions employ a double-layer of encoding, where the LRCC $\left(\mathrm{Comp},E\right)$ is associated with an internal encoding scheme $\left({\mathrm{Enc}}^{\mathrm{in}},{\mathrm{Dec}}^{\mathrm{in}}\right)$, and Comp outputs a circuit $\hat{C}$ in which the gates and wires of the circuit *C* over F are replaced with *gadgets* and *bundles*. A *bundle*b is an encoding (according to ${\mathrm{Enc}}^{\mathrm{in}}$) of some b∈F, representing the value of a wire in *C*; and a *gadget*$G_{g}$ is a (Boolean or arithmetic) circuit over F which operates over bundles and emulates the operation of the corresponding gate *g*. More specifically, in addition to taking as input bundle-encodings of *g*’s inputs, $G_{g}$ has additional *masking inputs*. These masking inputs are encodings (according to ${\mathrm{Enc}}^{\mathrm{in}}$) of some *masking values* in $F^{*}$ with a *specific, pre-determined* structure (for example, in [42] a masking value is the all-0 string), which are used to obtain LR. We associate with $G_{g}$ a set $WF_{g}\subset F^{*}$ (for *Well-Formed*) which consists of all encodings of masking values with the “correct” structure. We say that the masking inputs of $G_{g}$ are *well formed* if they are in $WF_{g}$ (otherwise we say they are *ill-formed*). A gadget $G_{g}$ is guaranteed to emulate *g* when its inputs are valid encodings of inputs for *g*, and its masking inputs are well-formed. For example, if g=×, then for every $x_{1},x_{2}\in F$, for every bundle encodings $x_{i}\leftarrow {\mathrm{Enc}}^{\mathrm{in}}\left(x_{i}\right),i=1,2$, and for every well-formed masking input bundles m, $G_{g}\left(x_{1},x_{2},m\right)$ encodes $x_{1}\times x_{2}$.

**Remark** **8.** *We note that the double layer of encoding described above is implicit in most works on leakage resilience. The reason is that, as described in Section 1.1.2, standard LRCCs in the literature are described as* randomized *circuits that generate the needed randomness internally (sometimes, using “opaque” gates which are assumed to be leak-free), whereas we describe the LR circuit as deterministic, and provide the needed randomness as part of the input to the circuit. For this reason, we need to explicitly use a double layer of encoding.*

Leakage resilience of $\hat{C}$ will follow from a combination of the leakage resilience of the internal encoding scheme ${\mathrm{Enc}}^{\mathrm{in}}$, and the following leakage-resilience guarantee of the gadgets. If the masking inputs are uniformly distributed over ${\mathrm{Enc}}^{\mathrm{in}}\left(WF_{g}\right)$, then given any valid encodings as standard inputs to the gadget: (1) the outputs are random subject to encoding the correct output (as determined by the standard inputs and the gate operation); and (2) the internal wire values of G can be reconstructed (i.e., regenerated) in a low complexity class, where the reconstructed and actual wire values are statistically close.

The masking inputs for all gadgets of $\hat{C}$ are provided as part of the inputs to $\hat{C}$, using the double-layered encoding, as we now explain. To simplify the description, we focus on describing how this is performed for the LRCC of [42] (see Remark 9 below on how the double-layered encoding extends to other LRCCs as well). In the LRCC of [42], the masking values for *all gates g* are simply the all-0 string, and different gates only differ in the *length* of the string (i.e., the number of 0’s). Let M denote the upper bound on the number of masking values used by any gate *g*. Then $E=\left(\mathrm{Enc},\mathrm{Dec}\right)$ where Enc takes as input an $x\in F^{*}$, and a size bound S, and outputs an encoding $\left(x,m\right)={\mathrm{Enc}}^{\mathrm{in}}\left(x,0^{M\cdot S}\right)$. For a given a circuit *C* of size $\left|C\right|\leq S$, its leakage-resilient version $\hat{C}=\mathrm{Comp}\left(C\right)$ takes as input encodings of the form $\left(x,m\right)$, where x are the input bundles of $\hat{C}$, and m are used as the masking inputs of the gadgets of $\hat{C}$. Since each gadget uses at most M encodings from m, and $\hat{C}$ has at most S gadgets, m contains sufficiently-many encodings (according to ${\mathrm{Enc}}^{\mathrm{in}}$) of 0 to enable the evaluation of all gadgets of $\hat{C}$.

The computation in $\hat{C}$ is performed over encodings, so the outcome of this computation — at least as it was described above — results in an *encoding* of the output of *C*. Since $\hat{C}$ should have the same output as *C*, the final step in $\hat{C}$ consists of a decoder which decodes the output.

**Remark** **9.** *We note that the double-layer encoding idea described above naturally extends to other LRCCs as well. Indeed, all that is needed is that all gadgets use the same masking values (differing only in the number of masking values they use). In fact, this idea can also be used if different gadgets use different masking values, in which case m can include S sets of masking inputs, each set containing masking inputs for every possible gate g, from which $\hat{C}$ can choose the appropriate masking inputs for each gate. This will result in a correct and leakage resilient (albeit less efficient) construction.*

### 4.2. An SAT-Respecting LRCC for Arithmetic Circuits

In this section we describe a *SAT-respecting* LRCC of [8] for arithmetic circuits, which is based on the LRCC of [42]. We first explain why the LRCC of [42] (and similar LRCCs) are *not* SAT-respecting.

**Why are standard LRCCs not SAT-Respecting?** Recall that an LRCC is SAT-respecting if for every circuit *C*, the following holds: if its leakage-resilient variant $\hat{C}$ is satisfiable, then so is *C*. This is not the case for many LRCCs, and specifically for gadget-based ones such as [42,43,44]. Indeed, in such LRCCs, the correctness of the computation relies on the assumption that the masking inputs of the gadgets are well-formed. However, $\hat{C}$ does not necessarily emulate *C* if its masking inputs are *ill-formed*. In fact, in these LRCCs one can force the output of $\hat{C}$ to be any desired value by appropriately choosing the masking values. This property is *crucial* for leakage resilience, since the security proof uses such ill-formed masking inputs to switch the input from *x* to a different $x^{\prime }$. Therefore, the main challenge in obtaining the SAT-respecting property is to guarantee that the masking inputs are well-formed while still allowing the security reduction to use ill-formed masking inputs. Before describing how this is done, we make two comments. First, recall that (as noted above) in the leakage-resilience literature the masking inputs are usually assumed to be generated by leak-free components, or opaque gates. In particular, the outputs of these components are guaranteed to be distributed according to the correct distribution (i.e., be well-formed), and their internal wires are unavailable to the leakage function. To eliminate this trust assumption, we instead have these encodings provided as part of the input, and check their validity to achieve the SAT-respecting property. Second, we note that the LRCC of [18] *does* posses the property that $\hat{C}$ is satisfiable only if *C* is, but this LRCC only resists $AC^{0}$ leakage [45], a leakage class which does not seem sufficiently strong to contain the function one needs to apply to an NP-witness to obtain a PCP.

**Checking Validity of Encodings While Preserving Leakage Resilience.** The main technical ingredient in the SAT-respecting LRCC of [8] is their component which checks well-formedness of encodings in a leakage-resilient manner. The technique is reminiscent of the “2-key trick” of [46] (used to convert a CPA-secure encryption scheme into a CCA-secure one) where they hold two copies of *C*, and in the security reduction one of the copies is used to achieve the SAT-respecting property, whereas the other is used to obtain leakage resilience. This component is tailored to the LRCC of [42], exploiting the fact that well-formed masking inputs of [42] are simply encodings of the all-0 string.

More specifically, the SAT-respecting LRCC of [8] consists of 3 parts. The first is two copies ${\hat{C}}_{1},{\hat{C}}_{2}$ of the circuit $\hat{C}$, obtained using the LRCC of [42]. The second is a mask-checking component $C_{0-\mathrm{check}}$, which checks that at least one of the copies ${\hat{C}}_{1},{\hat{C}}_{2}$ uses well-formed masking inputs. The important point is that, for the security proof to go through, $C_{0-\mathrm{check}}$ must hide *which* of the copies uses well-formed masking inputs. (This is because in the security proof first ${\hat{C}}_{1}$ and then ${\hat{C}}_{2}$ use ill-formed masking inputs; for the hybrids to be indistinguishable it must be the case that one cannot distinguish between the two cases.) As it turns out, this can be achieved by replacing $C_{0-\mathrm{check}}$ with its leakage-resilient version ${\hat{C}}_{0-\mathrm{check}}$ (obtained using the LRCC of [42]). This now introduces a further complication since ${\hat{C}}_{0-\mathrm{check}}$ has masking inputs of its own, the well-formedness of which must be verified. Indeed, if ${\hat{C}}_{0-\mathrm{check}}$ is allowed to use any masking inputs, then it is no longer guaranteed to emulate $C_{0-\mathrm{check}}$. In particular, by using ill-formed masking inputs in ${\hat{C}}_{0-\mathrm{check}}$ one can flip its output, thus causing it to accept even when the masking inputs of *both* ${\hat{C}}_{1},{\hat{C}}_{2}$ are ill-formed, rendering the ${\hat{C}}_{0-\mathrm{check}}$ component completely useless. The third component—a mask decoder $C_{\mathrm{dec}}$—is used to guarantee that ${\hat{C}}_{0-\mathrm{check}}$ uses well-formed masking inputs. Using a double-layer of mask checking (i.e., checking the masks of ${\hat{C}}_{1},{\hat{C}}_{2}$, and then checking the masks of the mask-checker) is helpful because the computations in ${\hat{C}}_{0-\mathrm{check}}$ are *not directly related* to the inputs of ${\hat{C}}_{1},{\hat{C}}_{2}$. As such, the masking inputs of ${\hat{C}}_{0-\mathrm{check}}$ can be *checked directly* by simply decoding them (and checking that all decoded values are 0) without violating LR. We now formally describe each of the components, and the resultant SAT-respecting LRCC.

**The Mask Checker ${\hat{C}}_{0-\mathrm{check}}$.** The mask checker verifies that at least one of the copies ${\hat{C}}_{1},{\hat{C}}_{2}$ uses well-formed masking inputs, while hiding which one. Recall that in the LRCC of [42] well-formed masking inputs are encodings of the all-0 string. Thus, if ${\vec{m}}^{1},{\vec{m}}^{2}$ denote the masking values whose encodings are used in ${\hat{C}}_{1},{\hat{C}}_{2}$, it suffices to check that $m_{j}^{1}\times m_{l}^{2}=0$ for every j,l. (We note that this check assumes that any masking input is a valid encoding — according to ${\mathrm{Enc}}^{\mathrm{in}}$ — of *some* masking value, namely that the internal encoding scheme is onto. This is indeed the case for the internal encoding scheme used in [42].) We construct $C_{0-\mathrm{check}}$ in two stages. We first describe a “binarization” component T which checks that a given field element is 0, then use it to construct $C_{0-\mathrm{check}}$.

**Notation** **6** (“Binarization” component T). *T:F→F is defined as $T\left(z\right)=-\prod_{0\neq a\in F}\left(z-a\right)$, computed using $O\left(\left|F\right|\right)$ constant and × gates arranged in $O\left(\log\left|F\right|\right)$ layers. Notice that*

$$T\left(z\right)=\left\{\begin{array}{cc} 1 & if\;z=0\\ 0 & if\;z\neq 0. \end{array}\right.$$

**Notation** **7** (Mask Checker $C_{0-\mathrm{check}}$). *Let M∈N. The mask checker $C_{0-\mathrm{check}}:F^{M}\times F^{M}\to F$ is defined as follows. $C_{0-\mathrm{check}}\left(y,z\right)=\prod_{i,j\in \left[M\right]}T\left(y_{i}\times z_{j}\right)$, computed using a multiplication tree of size $O\left(M^{2}\right)$ and depth $O\left(\log M\right)$ (on top of the multiplication trees used to compute T). Notice that*

$$C_{0-\mathrm{check}}\left(y,z\right)=1\Leftrightarrow T\left(y_{i},z_{j}\right)=1\;\forall i,j\in \left[M\right]\Leftrightarrow y=0^{M}\vee z=0^{M}.$$

**The mask decoder $C_{\mathrm{dec}}$.** As noted above, the mask decoder simply decodes the masking inputs used in ${\hat{C}}_{0-\mathrm{check}}$, and checks that all decoded values are 0. Decoding is performed using the decoder ${\mathrm{Dec}}^{\mathrm{in}}$ of the internal encoding procedure of [42]. We note that this encoding procedure is linear, and so decoding is performed simply by computing the inner product of the input encoding with some fixed vector (e.g., the all-1 vector). In particular, for each encoding length $\hat{n}$, the corresponding decoding circuit ${\mathrm{Dec}}^{\mathrm{in}}$ can be implemented using $O\left(\hat{n}\right)$ gates arranged in $O\left(\log\hat{n}\right)$ layers.

**Notation** **8** (Mask Decoder $C_{\mathrm{dec}}$). *Let $M_{0}\in N$, and let $\hat{n}$ denote the encoding length of $\left({\mathrm{Enc}}^{\mathrm{in}},{\mathrm{Dec}}^{\mathrm{in}}\right)$. The mask decoder $C_{\mathrm{dec}}:{\left(F^{\hat{n}}\right)}^{M_{0}}\to F$, on input $r=\left(r_{1},\ldots ,r_{M_{0}}\right)$ (where $r_{i}\in F^{\hat{n}}$ for every $1\leq i\leq M_{0}$) outputs $\prod_{i\in \left[M_{0}\right]}T\left({\mathrm{Dec}}^{\mathrm{in}}\left(r_{i}\right)\right)$. Notice that $C_{\mathrm{dec}}$ outputs 1 if and only if all $r_{i}$’s are well-formed, otherwise it outputs 0. $C_{\mathrm{dec}}$ is computed using $O\left(M_{0}\right)$-many × gates, arranged in a tree of depth $O\left(\log M_{0}\right)$ (on top of the sub-circuits $T\circ {\mathrm{Dec}}^{\mathrm{in}}$).*

**Figure 2:**
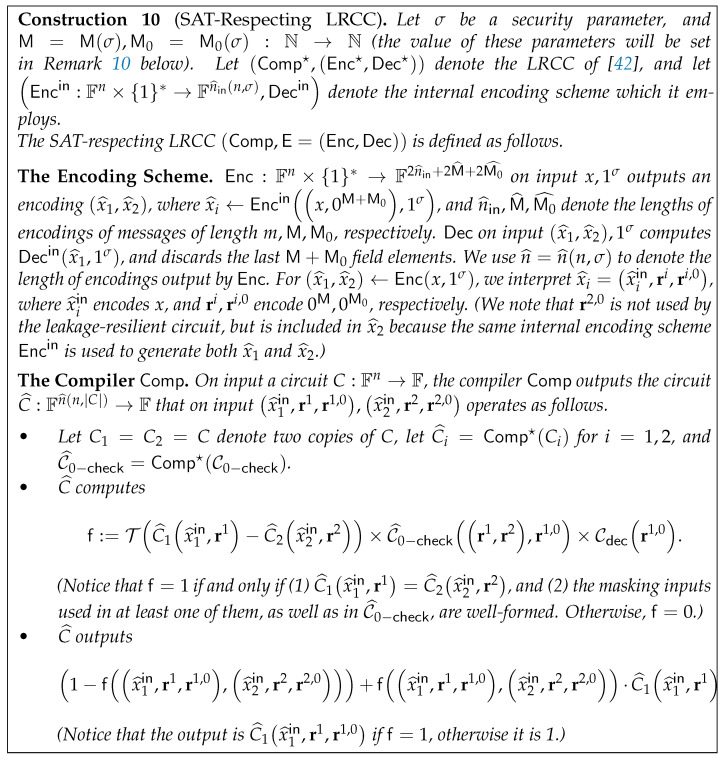
SAT-Respecting LRCC [8].

**The SAT-Respecting LRCC.** Having defined the three components of the SAT-respecting LRCC, we are now ready to describe the compiler itself, which is given in Figure 2.

**Remark** **10** (Setting the parameters). *Let $M^{☆}=M^{☆}\left(\sigma \right)$ denote the maximal number of masking inputs used in a gadget of ${\mathrm{Comp}}^{☆}$, and $S_{0}\left(M^{\prime }\right)$ denote the size of $C_{0-\mathrm{check}}$ on inputs of length $M^{\prime }$. Then $M\left(\sigma \right)=\sigma \cdot M^{☆}$ and $M_{0}\left(\sigma \right)=M^{☆}\cdot S_{0}\left(M\right)=M^{☆}\cdot S_{0}\left(\sigma \cdot M^{☆}\right)$.*

The following claim summarizes the properties of Construction 10. Roughly, it states that if the internal encoding scheme used in Construction 10 is leakage resilient against a leakage family $LEAK_{E}$, then Construction 10 is an SAT-respecting LRCC against a slightly weaker leakage family LEAK. Formally,

**Claim** **11** (SAT-respecting LRCC over F). *Let $LEAK,LEAK_{E}$ be families of functions, $S\left(n\right):N\to N$ be a size function, and $\epsilon \left(n\right):N\to R^{+}$. Let $E^{\mathrm{in}}=\left({\mathrm{Enc}}^{\mathrm{in}},{\mathrm{Dec}}^{\mathrm{in}}\right)$ be a linear, onto, $\left(LEAK_{E},\epsilon \left(n\right)\right)$-leakage-resilient encoding scheme with parameters n, σ and $\hat{n}=\hat{n}\left(n,\sigma \right)$, such that $LEAK_{E}=LEAK\circ SHALLOW\left(7,O\left({\hat{n}}^{4}\left(1,S\left(n\right)\right)\cdot S\left(n\right)\right)\right)$. Then, there exists an SAT-respecting, $\left(LEAK,4\epsilon \left(n\right)\cdot \left(\hat{n}\left(1,S\left(n\right)\right)+1\right)\cdot S\left(n\right),S\left(n\right)\right)$-LRCC over F. Moreover, for every $C:F^{n}\to F$, the compiled circuit $\hat{C}$ has size $\left|\hat{C}\right|=O\left({\left|F\right|}^{2}\cdot {\hat{n}}^{4}\left(1,S\left(n\right)\right)\cdot {\left|C\right|}^{2}\right)$.*

**Proof.**  The SAT-respecting property follows from Lemma 2. The leakage resilience property follows from Lemma 3. As for the size $\left|\hat{C}\right|$ of the leakage-resilient version $\hat{C}$ of *C*, in each of the copies ${\hat{C}}_{1},{\hat{C}}_{2}$ each gate is replaced with a size-$O\left({\hat{n}}^{2}\left(1,S\left(n\right)\right)\right)$ gadget (this is the size of gadgets generated by ${\mathrm{Comp}}^{☆}$, see Fact 12 below), So $\left|{\hat{C}}_{1}\right|,\left|{\hat{C}}_{2}\right|\leq O\left({\hat{n}}^{2}\left(1,S\left(n\right)\right)\cdot \left|C\right|\right)$. Since each gadget uses at most $O\left(\hat{n}\left(1,S\left(n\right)\right)\right)$ masking inputs (see Fact 12 below), then $C_{0-\mathrm{check}}$ contains $O\left({\hat{n}}^{2}\left(1,S\left(n\right)\right)\cdot {\left|C\right|}^{2}\right)$ “binarization” components T, each of size at most $O\left(\left|F\right|\right)$, arranged in a tree with $O\left({\hat{n}}^{2}\left(1,S\left(n\right)\right)\cdot {\left|C\right|}^{2}\right)$ multiplications, so $\left|C_{0-\mathrm{check}}\right|\leq O\left(\left|F\right|\cdot {\hat{n}}^{2}\left(1,S\left(n\right)\right)\cdot {\left|C\right|}^{2}\right)$, and consequently $\left|{\hat{C}}_{0-\mathrm{check}}\right|\leq O\left(\left|F\right|\cdot {\hat{n}}^{4}\left(1,S\left(n\right)\right)\cdot {\left|C\right|}^{2}\right)$. Finally, $C_{\mathrm{dec}}$ contains a decoding sub-circuit for each of the $O\left(\hat{n}\left(1,S\left(n\right)\right)\right)$ masking inputs used in the $O\left(\left|F\right|\cdot {\hat{n}}^{2}\left(1,S\left(n\right)\right)\cdot {\left|C\right|}^{2}\right)$ gadgets of ${\hat{C}}_{0-\mathrm{check}}$. Because E is linear, each of these decoding sub-circuits consists of $\hat{n}\left(1,S\left(n\right)\right)$× gates followed by $\hat{n}\left(1,S\left(n\right)\right)$+ gates. In addition, $C_{\mathrm{dec}}$ contains a “binarization” component T of size $O\left(\left|F\right|\right)$ for each decoding sub-circuit, followed by $O\left(\left|F\right|\cdot {\hat{n}}^{3}\left(1,S\left(n\right)\right)\cdot {\left|C\right|}^{2}\right)$× gates, so overall $\left|C_{\mathrm{dec}}\right|\leq O\left({\left|F\right|}^{2}\cdot {\hat{n}}^{3}\left(1,S\left(n\right)\right)\cdot {\left|C\right|}^{2}\right)$. □

The proof of Claim 11 used the following fact regarding the LRCC of [42].

**Fact** **12.** *Each gadget generated by the LRCC $\left({\mathrm{Comp}}^{☆},E^{☆}\right)$ of [42] has size at most $O\left({\hat{n}}^{2}\left(1,S\left(n\right)\right)\right)$, and uses at most $O\left(\hat{n}\left(1,S\left(n\right)\right)\right)$ masking inputs.*

**Lemma** **2.** *If $E^{\mathrm{in}}$ is linear and onto, then Construction 10 is SAT-respecting.*

**Proof.**  Assume that $\hat{C}\left(\hat{x}\right)=0$ for some $\hat{x}\in F^{\hat{n}}$, and denote $\hat{x}=\left(\left({\hat{x}}_{1}^{*},r^{1},r^{1,0}\right),\left({\hat{x}}_{2}^{*},r^{2},r^{2,0}\right)\right)$. We show that there exists an $x\in F^{n}$ such that $C\left(x\right)=0$. Since $\hat{C}\left(\hat{x}\right)=0$ then by the definition of $\hat{C}$, we have (1) f=1, and (2) ${\hat{C}}_{1}\left({\hat{x}}_{1}^{*},r^{1}\right)=0$. By (1), ${\hat{C}}_{1}\left({\hat{x}}_{1}^{*},r^{1}\right)={\hat{C}}_{2}\left({\hat{x}}_{2}^{*},r^{2}\right)$, and $C_{\mathrm{dec}},C_{0-\mathrm{check}}$ output 1. Notation 8 then guarantees that $r^{1,0}$ is well-formed which—by the correctness of ${\mathrm{Comp}}^{☆}$—guarantees that ${\hat{C}}_{0-\mathrm{check}}$ emulates $C_{0-\mathrm{check}}$. (Here, we also use the fact that $C_{\mathrm{dec}}$ is independent of all other components of, and inputs to, $\hat{C}$.) Moreover, since the encoding scheme is onto then $r^{1},r^{2}$ define inputs to $C_{0-\mathrm{check}}$ which cause it to output 1(because ${\hat{C}}_{0-\mathrm{check}}$ outputs 1, and its masking inputs are well-formed). Notation 7 then guarantees that at least one of $r^{1},r^{2}$ is well-formed. Assuming (without loss of generality) that $r^{1}$ is well-formed, then the correctness of ${\mathrm{Comp}}^{☆}$ guarantees that ${\hat{C}}_{1}$ emulates *C*, so $0={\hat{C}}_{1}\left({\hat{x}}_{1}^{*},r^{1}\right)=C\left(x\right)$, where $x\in F^{n}$ is obtained by computing $x={\mathrm{Dec}}^{\mathrm{in}}\left({\hat{x}}_{1}^{*}\right)$ (*x* is well-defined because $E^{\mathrm{in}}$ is onto). □

**Lemma** **3.** *If $E^{\mathrm{in}}$ is $\left(LEAK_{E},\epsilon \left(n\right)\right)$-leakage resilient, then for every function class LEAK such that $LEAK\circ SHALLOW\left(7,O\left({\hat{n}}^{5}\left(S\left(n\right)\right)\cdot S\left(n\right)\right)\right)\subseteq LEAK_{E}$, every circuit $C:F^{n}\to F$ of size $\left|C\right|\leq S\left(n\right)$, and every $x,y\in F^{n}$ such that $C\left(x\right)=C\left(y\right)$, it holds that $\left[\hat{C},\hat{x}\right]$ and $\left[\hat{C},\hat{y}\right]$ are $\left(LEAK,4\epsilon \left(n\right)\cdot \left(\hat{n}\left(1,S\left(n\right)\right)+1\right)\cdot S\left(n\right)\right)$-leakage resilient, where $\hat{x}\leftarrow \mathrm{Enc}\left(x,1^{\left|C\right|}\right)$ and $\hat{y}\leftarrow \mathrm{Enc}\left(y,1^{\left|C\right|}\right)$.*

#### Proof of Lemma 3

In this section we prove the leakage-resilience property of Construction 10. The analysis follows a (by now standard) proof paradigm for gadget-based LRCCs, but is more complex (compared to, e.g., the analysis in [42]) because of the mask-checker and mask-decoder components.

**High-Level Description of the Leakage-Resilience Argument.** Recall that the goal is to prove that leakage functions in some leakage class LEAK cannot distinguish between the wire values of $\hat{C}$ when evaluated on (encodings of) two different inputs x,y. This is achieved by reduction to the leakage resilience of the underlying encoding scheme E against leakage classes in a somewhat larger leakage class $LEAK_{E}$. The wire bundles of $\hat{C}$ are divided into two sets: *internal* bundles that are part of the internal computations in a gadget, and *external* bundles that connect two gadgets. The proof uses a hybrid argument in which we first replace all the internal bundles from carrying the real encodings to “simulated” encodings, and then replace all external bundles from encodings of the real values to encodings of random values. We then prove leakage resilience by showing that each pair of adjacent hybrids are computationally indistinguishable.

More specifically, consider two hybrid distributions $H,H^{\prime }$ in which we replace some external bundle *i*. Proving that $H\approx H^{\prime }$ is by reduction to the leakage resilience of the underlying encoding scheme, where we show that if H and $H^{\prime }$ are distinguishable by a function ℓ∈LEAK, then there exists a function $\ell_{E}\in LEAK_{E}$ that can distinguish between encodings of two different values $v,v^{\prime }$. The idea is that given an encoding e of either *v* or $v^{\prime }$, $\ell_{E}$ would use e to generate *the entire hybrid distribution* (which would be either H or $H^{\prime }$, depending on whether e encodes *v* or $v^{\prime }$, respectively), then evaluate *ℓ*. It turns out that the only wires of $H,H^{\prime }$ that depend on e are the internal wire bundles of the (at most two) gadgets that “touch” bundle *i*, in the sense that bundle *i* is either an input or output bundle of the gadget. Thus, while all other wire bundles can be hard-wired into $\ell_{E}$, it would still need to generate the internal wire bundles of the gadget(s) that touch bundle *i*. In particular, the computational complexity of $\ell_{E}$ would be higher than that of *ℓ*, which causes a loss in leakage resilience. It is therefore imperative that the internal wire values of the gadgets could be generated from their inputs and outputs by a function in a low complexity class. These functions are called *local reconstructors*. The crucial point here is that the local reconstructor is given not only the gadget’s inputs, but also its *outputs* (otherwise, it would be impossible to generate the internal wires in a low complexity class, because some of the gadgets perform complex computations). Before delving deeper into the details of the analysis, we first set the needed terminology regarding gadgets and their local reconstructors.

**Properties of Gadgets.** The analysis will use two properties of gadgets. The first is *local reconstructibility*: for every pair of “legal” input and output encodings, the internal wires of the gadget (as determined by the input-output pair, and its masking inputs), can be simulated in a low complexity class.

**Definition** **14** (Local reconstructibility). *Let G be a gadget and WF denote its set of well-formed masking inputs. A pair $\left(x,y\right)$ of encodings is* plausible *for G if $G\left(x,m\right)=y$ for some m∈WF. For ϵ>0, and families $LEAK,F_{G}$ of functions, G is $\left(LEAK,\epsilon \right)$*-reconstructible by *$F_{G}$ if the following holds. There exists a distribution REC over functions rec such that:*

- *$\mathrm{Supp}\left(\mathrm{REC}\right)\subseteq F_{G}$.*
- *rec takes as input G’s inputs and output, and simulates the masking inputs, and internal wires, of G.*
- *The following distributions are $\left(LEAK,\epsilon \right)$-leakage resilient for any plausible pair $\left(x,y\right)$ for G: (1) $\mathrm{rec}\left(x,y\right)$ for rec←REC; and (2) the actual distribution of the wires of G (as determined by the distribution of the masking inputs), conditioned on x,y.*

We will need the following result of [42] which shows that all gadgets in their LRCC $\left({\mathrm{Comp}}^{☆},E^{☆}\right)$ are locally reconstructible in a low complexity class. (We note that Faust et al. [42] actually prove a stronger result, where some of the gadgets have local reconstructors in lower complexity classes than the one stated here, and indistinguishability holds regardless of the leakage resilience of E. For clarity reasons, we chose to give a simplified and weaker version of their results which nonetheless suffices for our needs.)

**Lemma** **4** (Gadgets are locally reconstructible [42]). *Let σ∈N be a security parameter, let n:N×N→N be a length parameter, let $\epsilon \left(n\right):N\to R^{+}$, and let $LEAK,LEAK_{E}$ be families of functions such that $LEAK_{E}=LEAK\circ SHALLOW\left(3,O\left(\hat{n}\left(1,\sigma \right)\right)\right)$. Let $\left({\mathrm{Comp}}^{☆},E^{☆}\right)$ denote the LRCC of [42], and let $E^{\mathrm{in}}$ denote the internal encoding scheme which it uses. If $E^{\mathrm{in}}$ is $\left(LEAK_{E},\epsilon \left(n\right)\right)$-leakage resilient, then all gadgets used in $\left({\mathrm{Comp}}^{☆},E^{☆}\right)$ are $\left(LEAK,\hat{n}\left(1,\sigma \right)\cdot \epsilon \left(n\right)\right)$-reconstructible by $SHALLOW\left(2,O\left({\hat{n}}^{2}\left(1,\sigma \right)\right)\right)$.*

The second property is that gadgets are *re-randomizing* in the sense that the encodings at the output of each gadget are uniform subject to encoding the “correct” value. Formally,

**Definition** **15** (Gadget Re-Randomization). *A gadget G with set WF of well-formed masking inputs is* re-randomizing *if for every standard input $x=\mathrm{Enc}\left(x\right)$, when the masking input is sampled m←WF then $G\left(x,m\right)$ is random subject to encoding the correct output (as determined by x, and the gate which G emulates).*

**The Hybrid Argument.** Let $C:F^{n}\to F$ be an arithmetic circuit of size $\left|C\right|\leq S\left(n\right)$, and let $x,y\in F^{n}$. We show that for every ℓ∈LEAK,

$$\mathrm{SD}\left(\ell \left[\hat{C},\hat{x}\right],\ell \left[\hat{C},\hat{y}\right]\right)\leq \epsilon^{\prime }\left(n\right)$$

where $\epsilon^{\prime }\left(n\right):=4\epsilon \left(n\right)\cdot \left(\hat{n}\left(1,S\left(n\right)\right)+1\right)\cdot S\left(n\right)$. We bound the statistical distance using a hybrid argument. We define:

$$H^{x}:=\left(\left[\hat{C_{1}},{\hat{x}}_{1},r^{1}\right],\left[\hat{C_{2}},{\hat{x}}_{2},r^{2}\right],\left[{\hat{C}}_{0-\mathrm{check}},r^{1},r^{2},r^{1,0}\right],\left[C_{\mathrm{dec}},r^{1,0}\right]\right)$$

$$H^{y}:=\left(\left[\hat{C_{1}},{\hat{y}}_{1},r^{1}\right],\left[\hat{C_{2}},{\hat{y}}_{2},r^{2}\right],\left[{\hat{C}}_{0-\mathrm{check}},r^{1},r^{2},r^{1,0}\right],\left[C_{\mathrm{dec}},r^{1,0}\right]\right)$$

$$H^{y,x}:=\left(\left[\hat{C_{1}},{\hat{y}}_{1},r^{1}\right],\left[\hat{C_{2}},{\hat{x}}_{2},r^{2}\right],\left[{\hat{C}}_{0-\mathrm{check}},r^{1},r^{2},r^{1,0}\right],\left[C_{\mathrm{dec}},r^{1,0}\right]\right)$$

and notice that $H^{x}$ are the wire values of $\hat{C}$ on input an encoding of *x*, $H^{y}$ are the wire values of $\hat{C}$ on input an encoding of *y*, and $H^{y,x}$ is a hybrid distribution, consisting of the wire values of $\hat{C}$ when the first copy ${\hat{C}}_{1}$ has input (an encoding according to ${\mathrm{Enc}}^{\mathrm{in}}$) of *x*, whereas the second copy ${\hat{C}}_{2}$ has input (an encoding according to ${\mathrm{Enc}}^{\mathrm{in}}$) of *y*. We note that $H^{y,x}$ is only used in the proof—it is never obtained in an actual evaluation of $\hat{C}$. The proof proceeds by showing that for every ℓ∈LEAK, $\mathrm{SD}\left(\ell \left(H^{x}\right),\ell \left(H^{y,x}\right)\right)$ and $\mathrm{SD}\left(\ell \left(H^{y,x}\right),\ell \left(H^{y}\right)\right)$ are upper bounded by $2\epsilon \left(n\right)\cdot \left(\hat{n}\left(1,S\left(n\right)\right)+1\right)\cdot S\left(n\right)$, through a sequence of hybrids.

**Bounding $\mathrm{SD}\left(\ell \left(H^{x}\right),\ell \left(H^{y,x}\right)\right)$ for a leakage function *ℓ*.** The difference between these distributions is that in $H^{x}$ both copies ${\hat{C}}_{1},{\hat{C}}_{2}$ of (the leakage-resilient version of) *C* are evaluated on input *x*, whereas in $H^{y,x}$ the first is evaluated on *y* (and the second on *x*). We bound the statistical distance through a sequence of hybrids, defined as follows.

$H_{\mathrm{in}}^{x}$: this hybrid distribution replaces the internal wires of gadgets, and is obtained by: (1) evaluating $\hat{C}$ honestly on $\hat{x}\leftarrow \mathrm{Enc}\left(x,1^{\left|C\right|}\right)$; (2) picking local reconstructors for all gadgets of ${\hat{C}}_{1}$ (such reconstructors exist by Lemma 4), and re-computing their internal wires using these reconstructors; and (3) re-evaluating ${\hat{C}}_{0-\mathrm{check}}$ on the new masking inputs generated for the gadgets of ${\hat{C}}_{1}$, by re-computing the internal wires of all gadgets $G^{\prime }$ of ${\hat{C}}_{0-\mathrm{check}}$, the inputs of which are masking inputs used in gadgets of ${\hat{C}}_{1}$—but without changing the masking inputs these $G^{\prime }$ gadgets use, or their outputs. Lemma 5 below shows that this is indeed possible. (Crucially, since re-evaluating ${\hat{C}}_{0-\mathrm{check}}$ does not influence its masking inputs, this does not influence the computation in $C_{\mathrm{dec}}$, so there is no need to re-evaluate it.)
$H_{\mathrm{ext}}^{x}$: this distribution replaces the external wires (i.e., wires connecting gadgets), and is obtained as follows:
  - *Generating the wires of ${\hat{C}}_{2}$:* encode $\hat{x}=\left(\left({\hat{x}}_{1},r^{1},r^{1,0}\right),\left({\hat{x}}_{2},r^{2},r^{2,0}\right)\right)\leftarrow \mathrm{Enc}\left(x,1^{\left|C\right|}\right)$, and honestly evaluate ${\hat{C}}_{2}$ on ${\hat{x}}_{2}$ with masking inputs $r^{2}$.
  - *Generating the wires of ${\hat{C}}_{1}$:* pick a random encoding $\mathrm{out}\leftarrow {\mathrm{Enc}}^{\mathrm{in}}\left(1,1^{\left|C\right|}\right)$ for the output of ${\hat{C}}_{1}$, and honestly compute the wires of the output decoder of ${\hat{C}}_{1}$. (As discussed in Section 4.1, ${\hat{C}}_{1}$ contains an output decoder, which is needed because the computations in ${\hat{C}}_{1}$ are performed over encodings.) Then, pick a random input $z\in_{R}F^{n}$ for ${\hat{C}}_{1}$ and encode ${\hat{z}}_{1}\leftarrow {\mathrm{Enc}}^{\mathrm{in}}\left(z,1^{\left|C\right|}\right)$. Next, pick random encodings (according to ${\mathrm{Enc}}^{\mathrm{in}}$) for the outputs of all gadgets (except the gadgets the outputs of which are the inputs of the output decoder, since the outputs of these gadgets have already been fixed). This effectively determines the standard inputs, and outputs, of all gadgets of ${\hat{C}}_{1}$. Next, pick local reconstructors for all gadgets of ${\hat{C}}_{1}$, and use them to compute the internal wires of the gadgets. The reconstructors determine the (possibly ill-formed) masking inputs of the gadgets, which we denote by $r^{1\prime }$. $r^{1\prime }$ together with $r^{2}$ form the standard inputs of ${\hat{C}}_{0-\mathrm{check}}$.
  - *Generating the wires of ${\hat{C}}_{0-\mathrm{check}}$:* Evaluates ${\hat{C}}_{0-\mathrm{check}}$ on $r^{1\prime },r^{2}$, with masking inputs $r^{1,0}$.
  - *Generating the wires of $C_{\mathrm{dec}}$:* Evaluate $C_{\mathrm{dec}}$ on $r^{1,0}$.
  - Use the outputs of ${\hat{C}}_{1},{\hat{C}}_{2},{\hat{C}}_{0-\mathrm{check}},C_{\mathrm{dec}}$ to generate the flag f, and the output of $\hat{C}$.
  - $H_{\mathrm{ext}}^{x}$ consists of the concatenation of all these wire values.
$H_{\mathrm{ext}}^{y,x}$: this hybrid is generated similarly to $H_{\mathrm{ext}}^{x}$, except that instead of evaluating $\hat{C}$ on an encoding of *x*, we use the internal encoding scheme to generate encodings of $\left({\hat{y}}_{1},r^{1},r^{1,0}\right)$ and $\left({\hat{x}}_{2},r^{2},r^{2,0}\right)$ (where ${\hat{y}}_{1},{\hat{x}}_{2}$ encode y,x, respectively), and use them as inputs to ${\hat{C}}_{1},{\hat{C}}_{2}$, respectively.
$H_{\mathrm{in}}^{y,x}$: this hybrid is generated similarly to $H_{\mathrm{in}}^{x}$, except that instead of evaluating $\hat{C}$ on an encoding of *x*, we use $\left({\hat{y}}_{1},r^{1},r^{1,0}\right)$ and $\left({\hat{x}}_{2},r^{2},r^{2,0}\right)$ as inputs to ${\hat{C}}_{1},{\hat{C}}_{2}$, respectively.

We now bound the statistical distance between the outputs of leakage functions on each pair of adjacent hybrids. The following notation will be useful.

**Notation** **9.** *We say that a gadget $G^{\prime }$ of ${\hat{C}}_{0-\mathrm{check}}$ is* connected *to a gadget $G_{1}$${\hat{C}}_{1}$ (alternatively, a gadget $G_{2}$ of ${\hat{C}}_{2}$) if an input of $G^{\prime }$ is a masking input of $G_{1}$ (alternatively, $G_{2}$).*

**Bounding $\mathrm{SD}\left(\ell \left(H^{x}\right),\ell \left(H_{\mathrm{in}}^{x}\right)\right)$:** we show that $\mathrm{SD}\left(\ell \left(H^{x}\right),\ell \left(H_{\mathrm{in}}^{x}\right)\right)\leq \epsilon \left(n\right)\cdot \hat{n}\left(1,S\left(n\right)\right)\cdot S\left(n\right)$ for all $\ell \in LEAK^{\prime }$ such that $LEAK^{\prime }\circ SHALLOW\left(5,O\left({\hat{n}}^{5}\left(1,S\left(n\right)\right)\cdot S\left(n\right)\right)\right)\subseteq LEAK_{E}$. We use a hybrid argument, replacing the internal wires of the $M\leq S\left(n\right)$ gadgets of ${\hat{C}}_{1}$ one at a time. We define hybrids $H_{0},\ldots ,H_{M}$ where $H_{i}$ is obtained by evaluating $\hat{C}$ on (an encoding of) *x*, then recomputing the internal wires of *the first i gadgets of ${\hat{C}}_{1}$* using their local reconstructors. Additionally, we recompute the internal wires of gadgets $G^{\prime }$ of ${\hat{C}}_{0-\mathrm{check}}$ connected to one of these *i* gadgets, but *without changing the masking inputs of $G^{\prime }$* (this is possible by Lemma 5 below). Then $H_{0}=H^{x},H_{M}=H_{\mathrm{in}}^{x}$. To show that $\mathrm{SD}\left(\ell \left(H^{x}\right),\ell \left(H_{\mathrm{in}}^{x}\right)\right)\leq \epsilon \left(n\right)\cdot \hat{n}\left(1,S\left(n\right)\right)\cdot S\left(n\right)$ for all $\ell \in LEAK^{\prime }$, we show that for every $m\in \left[M\right]$, and any $\ell \in LEAK^{\prime }$, it holds that $\mathrm{SD}\left(\ell \left(H_{m}\right),\ell \left(H_{m-1}\right)\right)\leq \epsilon \left(n\right)\cdot \hat{n}\left(1,S\left(n\right)\right)$. Denote the *m*’th gadget by G. We use Lemma 4 to show that this follows from the leakage resilience of the internal encoding scheme $E_{\mathrm{in}}$. For this, we will need to generate—in a low complexity class—the entire hybrid distributions given only the (either real or reconstructed) internal wires of G. The problem is that since the internal wires of G contain also its masking inputs, changing them affects also computations in ${\hat{C}}_{0-\mathrm{check}}$. The following lemma from [8] states that the influence of modifying the masking inputs of G can be blocked, specifically: (1) that it only affects the internal wires of gadgets $G^{\prime }$ of ${\hat{C}}_{0-\mathrm{check}}$ connected to G, and more importantly (2) these internal wire values can be reconstructed *without* changing the masking inputs used in $G^{\prime }$.

**Lemma** **5** (Restating of Lemma 3.13 of [47]). *Let $G^{\prime }$ be a gadget of ${\hat{C}}_{0-\mathrm{check}}$ connected to gadgets of ${\hat{C}}_{1},{\hat{C}}_{2}$. Then for any fixed well-formed inputs $r^{1},r^{2}$ from gadgets of ${\hat{C}}_{1},{\hat{C}}_{2}$ (respectively), and any fixed well-formed masking inputs m for $G^{\prime }$, the following holds. For any $r^{1\prime }$, the internal wires of $G^{\prime }$ on input $r^{1\prime },r^{2},m$ can be computed in $SHALLOW\left(2,O\left({\hat{n}}^{2}\left(1,S\left(n\right)\right)\right)\right)$ given $r^{1\prime }$ and the output out of $G^{\prime }$ when evaluated on input $r^{1},r^{2}$ and masking inputs m.*

Using an averaging argument, we can fix all wires of ${\hat{C}}_{2}$, and all wires of ${\hat{C}}_{1}$ except the internal wires of G (its input and output wires *can* be fixed). Lemma 5 (which can be used because ${\hat{C}}_{2}$ and ${\hat{C}}_{0-\mathrm{check}}$ use well-formed masking inputs) shows that we can further fix all wires of ${\hat{C}}_{0-\mathrm{check}}$ and $C_{\mathrm{dec}}$, except for the internal wires of all gadgets $G^{\prime }$ of ${\hat{C}}_{0-\mathrm{check}}$ connected to G (crucially, the masking inputs of these gadgets *can* be fixed).

Consequently, given the wire values of G (either the real values $W_{R}$, or the reconstructed wires $W_{S}$), we can generate the entire hybrid distribution (either $H_{m-1}$ or $H_{m}$, respectively) in $SHALLOW\left(2,O\left({\hat{n}}^{5}\left(1,S\left(n\right)\right)\cdot S\left(n\right)\right)\right)$ by recomputing the internal wires of each of the (at most) $O\left({\hat{n}}^{3}\left(1,S\left(n\right)\right)\cdot S\left(n\right)\right)$ gadgets of ${\hat{C}}_{0-\mathrm{check}}$ connected to G. (Here, we use Fact 12—G uses at most $\hat{n}\left(1,S\left(n\right)\right)$ masking inputs; ${\hat{C}}_{2}$ has at most $O\left(\hat{n}\left(1,S\left(n\right)\right)\cdot S\left(n\right)\right)$ gadgets, each using at most $\hat{n}\left(1,S\left(n\right)\right)$ masking inputs; and each masking input of G is connected to every masking input used in ${\hat{C}}_{2}$.) Using Lemma 5, the internal wires of each $G^{\prime }$ can be computed in $SHALLOW\left(2,O\left({\hat{n}}^{2}\left(1,S\left(n\right)\right)\right)\right)$, and all these computations can be performed in parallel. Therefore, the hybrid distributions can be generated in $SHALLOW\left(2,O\left({\hat{n}}^{5}\left(1,S\left(n\right)\right)\right)\cdot S\left(n\right)\right)$. By the assumption of Claim 11, $E^{\mathrm{in}}$ is $\left(LEAK_{E},\epsilon \left(n\right)\right)$-leakage resilient, which by Lemma 4 implies that $W_{R},W_{S}$ are $\left(LEAK^{\prime \prime },\epsilon \left(n\right)\cdot \hat{n}\left(1,S\left(n\right)\right)\right)$-leakage resilient for any class $LEAK^{\prime \prime }$ of leakage functions such that $LEAK^{\prime \prime }\circ SHALLOW\left(3,O\left(\hat{n}\left(1,S\left(n\right)\right)\right)\right)\subseteq LEAK_{E}$. We now claim that $H_{m-1},H_{m}$ are $\left(LEAK^{\prime },\epsilon \left(n\right)\cdot \hat{n}\left(1,S\left(n\right)\right)\right)$-leakage resilient for every family $LEAK^{\prime }$ of leakage functions such that $LEAK^{\prime }\circ SHALLOW\left(2,O\left({\hat{n}}^{5}\left(1,S\left(n\right)\right)\cdot S\left(n\right)\right)\right)\subseteq LEAK^{\prime \prime }$, i.e., for any $LEAK^{\prime }$ such that $LEAK^{\prime }\circ SHALLOW\left(5,O\left({\hat{n}}^{5}\left(1,S\left(n\right)\right)\cdot S\left(n\right)\right)\right)\subseteq LEAK_{E}$. Indeed, this follows from the following lemma of [42] (by choosing F to be a singleton containing the function described above which generates $H_{m-1},H_{m}$ from $W_{R},W_{S}$).

**Lemma** **6** ([42]). *Let n∈N, let $W_{R},W_{S}$ be distributions over $F^{n}$, let LEAK,F be families of functions, and let ϵ>0. Let D be a distribution over functions in F of input length n. For f←D, let $W_{R}^{\prime }:=f\left(W_{R}\right),W_{S}^{\prime }:=f\left(W_{S}\right)$. If $W_{R},W_{S}$ are $\left(LEAK,\epsilon \right)$-leakage resilient, then $W_{R}^{\prime },W_{S}^{\prime }$ are $\left(LEAK^{\prime },\epsilon \right)$-leakage resilient for any family $LEAK^{\prime }$ of leakage functions such that $LEAK^{\prime }\circ F\subseteq LEAK$.*

Using Lemma 6, for any $\ell \in LEAK^{\prime }$ we have

$$\mathrm{SD}\left(\ell \left(H_{m}\right),\ell \left(H_{m-1}\right)\right)=\mathrm{SD}\left(\ell \left(W_{S}\right),\ell \left(W_{R}\right)\right)\leq \epsilon \left(n\right)\cdot \hat{n}\left(1,S\left(n\right)\right).$$

**Bounding $\mathrm{SD}\left(\ell \left(H_{\mathrm{ext}}^{x}\right),\ell \left(H_{\mathrm{in}}^{x}\right)\right)$:** We show that $\mathrm{SD}\left(\ell \left(H_{\mathrm{ext}}^{x}\right),\ell \left(H_{\mathrm{in}}^{x}\right)\right)\leq \epsilon \left(n\right)\cdot S\left(n\right)$ for all $\ell \in LEAK^{\prime }$ such that $LEAK^{\prime }\circ SHALLOW\left(4,O\left({\hat{n}}^{4}\left(1,S\left(n\right)\right)\cdot S\left(n\right)\right)\right)\subseteq LEAK_{E}$. We again use a hybrid argument, this time replacing the $M\leq S\left(n\right)$ inputs bundles of ${\hat{C}}_{1}$ (i.e., bundles corresponding to input wires of *C*), and bundles at the output of gadgets of ${\hat{C}}_{1}$ (except for bundles that are used as input of the output decoder of ${\hat{C}}_{1}$). We define hybrids $H_{0},\ldots ,H_{M}$, where $H_{i}$ is generated as follows. We evaluate $\hat{C}$ on a random encoding of *x*. Then, we replace the first *i* bundles with random encodings of random values, except if one of these bundles corresponds to the output wire of *C*, in which case we replace it with a random encoding of 1. Finally, we recompute the internal wires of the gadgets of ${\hat{C}}_{1}$ using their gadget reconstructors (Lemma 4), and recompute (using Lemma 5) the internal wires (except the masking inputs and outputs) of all gadgets $G^{\prime }$ of ${\hat{C}}_{0-\mathrm{check}}$ connected to one of these gadgets. In particular, since the inputs of ${\hat{C}}_{0-\mathrm{check}}$ contain also masking inputs used in gadgets of ${\hat{C}}_{1}$, this re-computation of wires of ${\hat{C}}_{0-\mathrm{check}}$ uses the masking inputs generated by the reconstructors (for any gadget of ${\hat{C}}_{1}$ whose internal wires were re-constructed). Then $H_{0}=H_{\mathrm{in}}^{x}$ and $H_{M}=H_{\mathrm{ext}}^{x}$. Therefore, to prove that $\mathrm{SD}\left(\ell \left(H_{\mathrm{ext}}^{x}\right),\ell \left(H_{\mathrm{in}}^{x}\right)\right)\leq \epsilon \left(n\right)\cdot S\left(n\right)$ for all $\ell \in LEAK^{\prime }$, it suffices to prove that $\mathrm{SD}\left(\ell \left(H_{m}\right),\ell \left(H_{m-1}\right)\right)\leq \epsilon \left(n\right)$ for all $m\in \left[M\right]$. Let $G_{o}$ ($G_{i}$) denote the gadget the output (input) of which is the *m*’th bundle. (If the *m*’th bundle is an input bundle, then we consider only the gadget $G_{i}$.) Using an averaging argument, we can fix all wires in $H_{m},H_{m-1}$ except for: the *m*’th bundle; the masking inputs, outputs, and internal wires of $G_{o}$; the masking inputs, and internal wires, of $G_{i}$, as well as its input wire corresponding to the *m*’th bundle; and the internal wires of all gadgets $G^{\prime }$ of ${\hat{C}}_{0-\mathrm{check}}$ connected to $G_{o}$ or $G_{i}$ (except for the masking inputs and the output of $G^{\prime }$, which *can* be fixed, see Lemma 5).

Let *b* denote the value encoded by the *m*’th bundle in $H_{\mathrm{in}}^{x}$. Let $W_{R}\leftarrow {\mathrm{Enc}}^{\mathrm{in}}\left(b,1^{S\left(n\right)}\right)$, and $W_{S}\leftarrow {\mathrm{Enc}}^{\mathrm{in}}\left(r,1^{S\left(n\right)}\right)$ for a random *r* (except when the *m*’th bundle corresponds to the output wire of *C*, in which case we set $W_{S}\leftarrow {\mathrm{Enc}}^{\mathrm{in}}\left(1,1^{S\left(n\right)}\right)$). Then $W_{R},W_{S}$ are distributed identically to the *m*’th bundle in $H_{m-1},H_{m}$, respectively. We define a distribution F over $SHALLOW\left(4,O\left({\hat{n}}^{4}\left(1,S\left(n\right)\right)\cdot S\left(n\right)\right)\right)$ as follows. Sampling a function f←F is performed by sampling ${\mathrm{rec}}_{o},{\mathrm{rec}}_{i}$ from the distribution over reconstructors for $G_{o},G_{i}$, respectively (see Definition 14). The function *f* has all the hard-wired values of $H_{m-1}$ hard-wired into it. On input $e\in F^{\hat{n}\left(1,S\left(n\right)\right)}$, *f* performs the following: (1) evaluates ${\mathrm{rec}}_{o}$ on the (hard-wired) inputs of $G_{o}$, and the output e (this reconstructs the masking inputs, and internal wires, of $G_{o}$); (2) evaluates ${\mathrm{rec}}_{i}$ on e as one of the inputs, and the other (hard-wired) input and output of $G_{i}$; and finally (3) for every gadget $G^{\prime }$ of ${\hat{C}}_{0-\mathrm{check}}$ connected to $G_{o}$ or $G_{i}$, generates its internal wires using Lemma 5 (without changing the output or masking inputs of $G^{\prime }$). Then $f\in SHALLOW\left(4,O\left({\hat{n}}^{4}\left(1,S\left(n\right)\right)\cdot S\left(n\right)\right)\right)$. Indeed, by Lemma 4, ${\mathrm{rec}}_{o},{\mathrm{rec}}_{i}\in SHALLOW\left(2,O\left({\hat{n}}^{2}\left(1,S\left(n\right)\right)\right)\right)$ (and given e, they can be evaluated in parallel). Moreover, given the internal wires of $G_{o},G_{i}$, the wires that need to be computed in a gadget $G^{\prime }$ connected to them are computable in $SHALLOW\left(2,O\left({\hat{n}}^{2}\left(1,S\left(n\right)\right)\right)\right)$ (by Lemma 5, and since the masking inputs of ${\hat{C}}_{2}$ and ${\hat{C}}_{0-\mathrm{check}}$ are well-formed). We conclude by noting that there are at most $O\left({\hat{n}}^{2}\left(1,S\left(n\right)\right)\right)\cdot S\left(n\right)$ such gadgets, and they can be evaluated in parallel.

Denote $W_{R}^{\prime }:=f\left(W_{R}\right),W_{S}^{\prime }:=f\left(W_{S})\right)$ for f←F, then $W_{R}^{\prime }\equiv H_{m-1}$ and $W_{S}^{\prime }\equiv H_{m}$ because the gadgets are re-randomizing (which, in particular, guarantees that these equivalences hold *despite* the fact some of the values in the computation were fixed in advance). By the assumption of Lemma 3, $W_{R},W_{S}$—which are encodings according to $E^{\mathrm{in}}$—are $\left(LEAK_{E},\epsilon \left(n\right)\right)$-leakage resilient. Therefore, by Lemma 6, $W_{R}^{\prime },W_{S}^{\prime }$ (and consequently also $H_{m-1},H_{m}$) are $\left(LEAK^{\prime },\epsilon \left(n\right)\right)$-leakage resilient for any $LEAK^{\prime }$ such that $LEAK^{\prime }\circ SHALLOW\left(4,O\left({\hat{n}}^{4}\left(1,S\left(n\right)\right)\cdot S\left(n\right)\right)\right)\subseteq LEAK_{E}$.

**Bounding $\mathrm{SD}\left(\ell \left(H_{\mathrm{ext}}^{x}\right),\ell \left(H_{\mathrm{ext}}^{y,x}\right)\right)$:** We show that $\mathrm{SD}\left(\ell \left(H_{\mathrm{ext}}^{x}\right),\ell \left(H_{\mathrm{ext}}^{y,x}\right)\right)=0$ for every *ℓ*. Indeed, $H_{\mathrm{ext}}^{x}\equiv H_{\mathrm{ext}}^{y,x}$ because the hybrids are independent of the input for ${\hat{C}}_{1}$ (since the input is re-sampled as a fresh $z\in_{R}F^{n}$ in both hybrids).

**Bounding $\mathrm{SD}\left(\ell \left(H_{\mathrm{ext}}^{y,x}\right),\ell \left(H_{\mathrm{in}}^{y,x}\right)\right)$:** We show that $\mathrm{SD}\left(\ell \left(H_{\mathrm{ext}}^{y,x}\right),\ell \left(H_{\mathrm{in}}^{y,x}\right)\right)\leq \epsilon \left(n\right)\cdot S\left(n\right)$ for all $\ell \in LEAK^{\prime }$ where $LEAK^{\prime }\circ SHALLOW\left(4,O\left({\hat{n}}^{4}\left(1,S\left(n\right)\right)\cdot S\left(n\right)\right)\right)\subseteq LEAK_{E}$. The proof is similar to the proof that $\mathrm{SD}\left(\ell \left(H_{\mathrm{ext}}^{x}\right),\ell \left(H_{\mathrm{in}}^{x}\right)\right)\leq \epsilon \left(n\right)\cdot S\left(n\right)$, because the argument was independent of the actual inputs used in ${\hat{C}}_{1},{\hat{C}}_{2}$ (as long as both hybrids use the same input in each copy).

**Bounding $\mathrm{SD}\left(\ell \left(H_{\mathrm{in}}^{y,x}\right),\ell \left(H^{y,x}\right)\right)$:** We show that $\mathrm{SD}\left(\ell \left(H_{\mathrm{in}}^{y,x}\right),\ell \left(H^{y,x}\right)\right)\leq \epsilon \left(n\right)\cdot \hat{n}\left(1,S\left(n\right)\right)\cdot S\left(n\right)$ for all $\ell \in LEAK^{\prime }$, where $LEAK^{\prime }\circ SHALLOW\left(5,O\left({\hat{n}}^{5}\left(1,S\left(n\right)\right)\cdot S\left(n\right)\right)\right)\subseteq LEAK_{E}$. The proof is similar to the proof that $\mathrm{SD}\left(\ell \left(H^{x}\right),\ell \left(H_{\mathrm{in}}^{x}\right)\right)\leq \epsilon \left(n\right)\cdot \hat{n}\left(1,S\left(n\right)\right)\cdot S\left(n\right)$, because the argument was independent of the actual inputs used in ${\hat{C}}_{1},{\hat{C}}_{2}$.

**Bounding $\mathrm{SD}\left(\ell \left(H^{y}\right),\ell \left(H^{y,x}\right)\right)$ for a leakage function *ℓ*.** The argument here follows the same blueprint as the one used to bound $\mathrm{SD}\left(\ell \left(H^{x}\right),\ell \left(H^{y,x}\right)\right)$, but is more involved because we now need to switch the input of the *second copy ${\hat{C}}_{2}$*. In particular, the wire values in this hybrid argument will no longer correspond to the values in an *actual* evaluation of ${\hat{C}}_{2}$. While the computations in ${\hat{C}}_{1}$ will still be performed honestly, we will no longer be able to claim that reconstructing the internal wires of gadgets of ${\hat{C}}_{2}$ (or the external wires connecting gadgets of ${\hat{C}}_{2}$) does not affect the computations in ${\hat{C}}_{0-\mathrm{check}}$ and $C_{\mathrm{dec}}$. This is because the manner in which ${\hat{C}}_{0-\mathrm{check}}$ uses the masking inputs from ${\hat{C}}_{1},{\hat{C}}_{2}$ is *not symmetric*, and in particular, resampling the masking inputs used in ${\hat{C}}_{2}$ (as is performed by the local reconstructors of gadgets of ${\hat{C}}_{2}$) *will* affect the computations in ${\hat{C}}_{0-\mathrm{check}}$, and will necessitate reevaluating it. This, in turn, will affect the masking inputs used in ${\hat{C}}_{0-\mathrm{check}}$, which will affect the computations in $C_{\mathrm{dec}}$. However, we cannot simply re-evaluate ${\hat{C}}_{0-\mathrm{check}}$ and $C_{\mathrm{dec}}$ in the hybrid argument. Indeed, this would require evaluating circuits of large depth, and the leakage resilience guarantee will therefore deteriorate significantly. Instead, Ishai et al. [8] use an alternative method of locally reconstructing the needed wire values of ${\hat{C}}_{0-\mathrm{check}},C_{\mathrm{dec}}$. Specifically, they show a low-depth reconstructor for the gadgets $G^{\prime }$ of ${\hat{C}}_{0-\mathrm{check}}$ connected to gadgets of ${\hat{C}}_{2}$, which generates the “correct” distribution if the input of $G^{\prime }$ from ${\hat{C}}_{1}$, and $G^{\prime }$’s output, are well formed. In particular, this implies that the internal wires of $G^{\prime }$ can be reconstructed without modifying its output. They also show a low-depth reconstructor for each decoding circuit of $C_{\mathrm{dec}}$, that generates the “correct” distribution if its inputs are well formed (again, without changing the output of these decoding circuits). Specifically, we will use the following results from [8].

**Lemma** **7** (Local reconstructors for ${\hat{C}}_{0-\mathrm{check}},C_{\mathrm{dec}}$, restatement of Lemmas 3.17 and 3.18 of [47]). *There exists a distribution REC over $SHALLOW\left(2,O\left({\hat{n}}^{2}\left(1,S\left(n\right)\right)\right)\right)$ such that the following holds for any gadget $G^{\prime }$ of ${\hat{C}}_{0-\mathrm{check}}$ connected to a gadget of ${\hat{C}}_{1}$ or ${\hat{C}}_{2}$. For every plausible pair $\left(\left(r^{1,0},r^{2,0}\right),c\right)$ for $G^{\prime }$ such that $r^{1,0}$ is well formed, when rec←REC then $\mathrm{rec}\left(r^{1,0},r^{2,0},c\right)$ is distributed identically to the wire values of $G^{\prime }$ in a real execution, conditioned on the input-output pair $\left(\left(r^{1,0},r^{2,0}\right),c\right)$. In particular, c and the masking inputs computed by rec are well-formed.*

*Moreover, for a fixed $\mathrm{rec}\in \mathrm{Supp}\left(\mathrm{REC}\right)$, and any fixed well-formed $r^{1,0},c$, there exists a function ${\mathrm{rec}}_{\mathrm{Dec}}^{r^{1,0},c,\mathrm{rec}}\in SHALLOW\left(2,O\left(\hat{n}\left(1,S\left(n\right)\right)\right)\right)$ such that the following holds for any input $r^{2,0}$ of $G^{\prime }$. If $\left(\left(r^{1,0},r^{2,0}\right),c\right)$ is a plausible pair for $G^{\prime }$, then ${\mathrm{rec}}_{\mathrm{Dec}}^{r^{1,0},c,\mathrm{rec}}\left(r^{2,0}\right)$ generates the wire values of the decoding sub-circuits of $C_{\mathrm{dec}}$, the inputs of which are the masking inputs of $G^{\prime }$ which rec generated. In particular, because the masking inputs generated by rec are well-formed, the outputs of these decoding sub-circuits are 0.*

The proof now follows using a hybrid argument. The hybrids are similar to the ones used to bound $\mathrm{SD}\left(\ell \left(H^{x}\right),\ell \left(H^{y,x}\right)\right)$, except that we will need to regenerate the internal wires of ${\hat{C}}_{0-\mathrm{check}},C_{\mathrm{dec}}$ using the reconstructors of Lemma 7. We now define the hybrids.

$H_{\mathrm{in}}^{y}$: this hybrid distribution replaces the internal wires of gadgets (similar to $H_{\mathrm{in}}^{x}$), and is obtained by: (1) evaluating $\hat{C}$ honestly on $\hat{y}\leftarrow \mathrm{Enc}\left(y,1^{\left|C\right|}\right)$; (2) picking local reconstructors for all gadgets of ${\hat{C}}_{2}$, and re-computing their internal wires using these reconstructors; (3) re-computing the internal wires of ${\hat{C}}_{0-\mathrm{check}}$ using rec←REC, where REC is the distribution of Lemma 7; and (4) re-computing the internal wires of $C_{\mathrm{dec}}$ using the functions ${\mathrm{rec}}_{\mathrm{Dec}}^{r^{1,0},c,\mathrm{rec}}$ defined in Lemma 7 (here, $r^{1,0},c$ are determined by the re-computed wires values of ${\hat{C}}_{0-\mathrm{check}}$);
$H_{\mathrm{ext}}^{y}$: this distribution replaces the external wires (similar to $H_{\mathrm{ext}}^{x}$), and is obtained as follows:
  - *Generating the wires of ${\hat{C}}_{1}$:* encode $\hat{y}=\left(\left({\hat{y}}_{1},r^{1},r^{1,0}\right),\left({\hat{y}}_{2},r^{2},r^{2,0}\right)\right)\leftarrow \mathrm{Enc}\left(y,1^{\left|C\right|}\right)$, and honestly evaluate ${\hat{C}}_{1}$ on ${\hat{y}}_{1}$ with masking inputs $r^{1}$;
  - *Generating the wires of ${\hat{C}}_{2}$:* pick a random input $z\in_{R}F^{n}$ for ${\hat{C}}_{2}$, and generate random encodings $\mathrm{out}\leftarrow {\mathrm{Enc}}^{\mathrm{in}}\left(1,1^{\left|C\right|}\right)$, ${\hat{z}}_{1}\leftarrow {\mathrm{Enc}}^{\mathrm{in}}\left(z,1^{\left|C\right|}\right)$ for the output and input of ${\hat{C}}_{2}$. Next, pick random encodings (according to ${\mathrm{Enc}}^{\mathrm{in}}$) for the outputs of all gadgets (except the gadgets whose outputs are the inputs of the output decoder, since the outputs of these gadgets have already been fixed). Then, pick local reconstructors for all gadgets of ${\hat{C}}_{2}$, and use them to compute the internal wires of the gadgets. The reconstructors determine the (possibly ill-formed) masking inputs $r^{2\prime }$ of the gadgets, which (together with $r^{1}$) form the standard inputs of ${\hat{C}}_{0-\mathrm{check}}$;
  - *Generating the wires of ${\hat{C}}_{0-\mathrm{check}}$:* for every gadget $G^{\prime }$ of ${\hat{C}}_{0-\mathrm{check}}$ connected to a gadget of ${\hat{C}}_{2}$, pick a reconstructor for it according to the distribution REC (Lemma 7) and use it to compute the internal wires of $G^{\prime }$. These reconstructors determine the inputs to the decoding sub-circuits of $C_{\mathrm{dec}}$;
  - *Generating the wires of $C_{\mathrm{dec}}$:* Use the functions of Lemma 7 to compute the internal wires of the decoding sub-circuits of the $C_{\mathrm{dec}}$;
  - Use the outputs of ${\hat{C}}_{1},{\hat{C}}_{2},{\hat{C}}_{0-\mathrm{check}},C_{\mathrm{dec}}$ to generate the flag f, and the output of $\hat{C}$;
  - $H_{\mathrm{ext}}^{y}$ consists of the concatenation of all these wire values.
$H_{\mathrm{ext},2}^{y,x}$: this hybrid is generated similarly to $H_{\mathrm{ext}}^{y}$, except that instead of evaluating $\hat{C}$ on an encoding of *y*, we use the internal encoding scheme to generate encodings of $\left({\hat{y}}_{1},r^{1},r^{1,0}\right)$ and $\left({\hat{x}}_{2},r^{2},r^{2,0}\right)$ (where ${\hat{y}}_{1},{\hat{x}}_{2}$ encode y,x, respectively), and use them as inputs to ${\hat{C}}_{1},{\hat{C}}_{2}$, respectively;
$H_{\mathrm{in},2}^{y,x}$: this hybrid is generated similarly to $H_{\mathrm{in}}^{y}$, except that instead of evaluating $\hat{C}$ on an encoding of *y*, we use $\left({\hat{y}}_{1},r^{1},r^{1,0}\right)$ and $\left({\hat{x}}_{2},r^{2},r^{2,0}\right)$ as inputs to ${\hat{C}}_{1},{\hat{C}}_{2}$, respectively.

The indistinguishability of the hybrids now follows similarly to the proof bounding $\mathrm{SD}\left(H^{x},H^{y,x}\right)$, and we only sketch the difference.

**Bounding $\mathrm{SD}\left(\ell \left(H^{y}\right),\ell \left(H_{\mathrm{in}}^{y}\right)\right)$:** We show that $\mathrm{SD}\left(\ell \left(H^{y}\right),\ell \left(H_{\mathrm{in}}^{y}\right)\right)\leq \epsilon \left(n\right)\cdot \hat{n}\left(1,S\left(n\right)\right)\cdot S\left(n\right)$ for all $\ell \in LEAK^{\prime }$ such that $LEAK^{\prime }\circ SHALLOW\left(7,O\left({\hat{n}}^{4}\left(1,S\left(n\right)\right)\cdot S\left(n\right)\right)\right)\subseteq LEAK_{E}$. We define the hybrids $H_{0},\ldots ,H_{M}$, where $H_{i}$ is obtained by: (1) evaluating $\hat{C}$ on (an encoding of) *y*, then recomputing the internal wires of the first *i* gadgets *of ${\hat{C}}_{2}$* using their local reconstructors; (2) *re-computing the internal wires of ${\hat{C}}_{0-\mathrm{check}}$* that are influenced by this re-computation of the first *i* gadgets of ${\hat{C}}_{2}$ (i.e., gadgets of ${\hat{C}}_{0-\mathrm{check}}$ that are connected to one of these *i* gadgets); and (3) (using the functions of Lemma 7) *recomputing the internal wires of $C_{\mathrm{dec}}$* that were influenced by re-computing ${\hat{C}}_{0-\mathrm{check}}$. Then $H_{0}=H^{y},H_{M}=H_{\mathrm{in}}^{y}$, and we show that $\mathrm{SD}\left(\ell \left(H_{m}\right),\ell \left(H_{m-1}\right)\right)\leq \epsilon \left(n\right)\cdot \hat{n}\left(1,S\left(n\right)\right)$ for every $m\in \left[M\right]$, and any $\ell \in LEAK^{\prime }$. Denote the *m*’th gadget by G. We can fix all wires of ${\hat{C}}_{1}$, all wires of ${\hat{C}}_{2}$ except the internal wires W of G (its input and output wires *can* be fixed), and all the internal wires of ${\hat{C}}_{0-\mathrm{check}},C_{\mathrm{dec}}$ that are influenced by W. (These consist of the internal wires—but not the output—of any gadget $G^{\prime }$ of ${\hat{C}}_{0-\mathrm{check}}$ connected to G, and the internal wires—but not the outputs—in the decoding sub-circuits of $C_{\mathrm{dec}}$ that decode masking inputs of $G^{\prime }$.)

We now describe a distribution F over $SHALLOW\left(4,O\left({\hat{n}}^{4}\left(1,S\left(n\right)\right)\cdot S\left(n\right)\right)\right)$, where given the wire values of G (either the real wire values $W_{R}$ or the reconstructed wires $W_{S}$), f∈F generates the entire hybrid distribution (either $H_{m-1}$ or $H_{m}$, respectively), as follows. For every gadget $G^{\prime }$ of ${\hat{C}}_{0-\mathrm{check}}$ connected to G, F chooses a reconstructor ${\mathrm{rec}}_{G^{\prime }}\leftarrow \mathrm{REC}$ (see Lemma 7). The function *f* has all the hard-wired values of $H_{m}$ hard-wired into it. On input W it evaluates the ${\mathrm{rec}}_{G^{\prime }}$’s on the masking inputs of G (as reported in W) and the hard-wired values, to generate the internal wires (including the masking inputs) of $G^{\prime }$, and then uses the functions defined in Lemma 7 to reconstruct the internal wires of the decoding sub-circuits of $C_{\mathrm{dec}}$ that decode the masking inputs of $G^{\prime }$. Then $f\in SHALLOW\left(4,O\left({\hat{n}}^{4}\left(1,S\left(n\right)\right)\cdot S\left(n\right)\right)\right)$ because by Lemma 7 the reconstructors for the $G^{\prime }$’s (the decoding sub-circuits, respectively) are computable in $SHALLOW\left(2,O\left({\hat{n}}^{2}\left(1,S\left(n\right)\right)\right)\right)$ ($SHALLOW\left(2,O\left(\hat{n}\left(1,S\left(n\right)\right)\right)\right)$, respectively); the reconstructors of all the (at most) $O\left({\hat{n}}^{2}\left(1,S\left(n\right)\right)\cdot S\left(n\right)\right)$ gadgets $G^{\prime }$ can be computed in parallel); and the reconstructors for all of the (at most) $O\left({\hat{n}}^{3}\left(1,S\left(n\right)\right)\cdot S\left(n\right)\right)$ decoding sub-circuits (each $G^{\prime }$ is connected to $O\left(\hat{n}\left(1,S\left(n\right)\right)\right)$ decoding sub-circuits) can be computed in parallel.

Since $W_{R},W_{S}$ are $\left(LEAK^{\prime },\epsilon \left(n\right)\cdot \hat{n}\left(1,S\left(n\right)\right)\right)$-leakage resilient for any class $LEAK^{\prime }$ of leakage functions such that $LEAK^{\prime }\circ SHALLOW\left(3,O\left(\hat{n}\left(1,\sigma \right)\right)\right)\subseteq LEAK_{E}$ (this follows from Lemma 4 because $E^{\mathrm{in}}$ is $\left(LEAK_{E},\epsilon \left(n\right)\right)$-leakage resilient), Lemma 6 guarantees that $H_{m-1},H_{m}$ are $\left(LEAK^{\prime \prime },\epsilon \left(n\right)\cdot \hat{n}\left(1,S\left(n\right)\right)\right)$-leakage resilient for every family $LEAK^{\prime \prime }$ of leakage functions such that $LEAK^{\prime \prime }\circ SHALLOW\left(4,O\left({\hat{n}}^{4}\left(1,S\left(n\right)\right)\cdot S\left(n\right)\right)\right)\subseteq LEAK^{\prime }$, i.e., for any $LEAK^{\prime \prime }$ such that $LEAK^{\prime \prime }\circ SHALLOW\left(7,O\left({\hat{n}}^{4}\left(1,S\left(n\right)\right)\cdot S\left(n\right)\right)\right)\subseteq LEAK_{E}$.

**Bounding $\mathrm{SD}\left(\ell \left(H_{\mathrm{ext}}^{y}\right),\ell \left(H_{\mathrm{in}}^{y}\right)\right)$:** We show that $\mathrm{SD}\left(\ell \left(H_{\mathrm{ext}}^{y}\right),\ell \left(H_{\mathrm{in}}^{y}\right)\right)\leq \epsilon \left(n\right)\cdot S\left(n\right)$ for all $\ell \in LEAK^{\prime }$ such that $LEAK^{\prime }\circ SHALLOW\left(6,O\left({\hat{n}}^{4}\left(1,S\left(n\right)\right)\cdot S\left(n\right)\right)\right)\subseteq LEAK_{E}$. The proof is by a hybrid argument in which we replace the input bundles *of ${\hat{C}}_{2}$*, and the bundles at the output of gadget *of ${\hat{C}}_{2}$*, one at a time. More specifically, we define hybrids $H_{0},\ldots ,H_{M}$, where $H_{i}$ is generated from $H_{\mathrm{in}}^{y}$, by: (1) replacing the first *i* bundles with random encodings of random values (except for the bundle corresponding to the output of ${\hat{C}}_{2}$, which is set to a random encoding of 1); (2) recomputing the internal wires of the first *i* gadgets *of ${\hat{C}}_{2}$* using the gadget reconstructors; (3) re-computing the internal wires of ${\hat{C}}_{0-\mathrm{check}}$ that are influenced by this re-computation of the first *i* gadgets *of ${\hat{C}}_{2}$*; and (4) (using the functions of Lemma 7) recomputing the internal wires of $C_{\mathrm{dec}}$ that were influenced by re-computing ${\hat{C}}_{0-\mathrm{check}}$. Then $H_{0}=H_{\mathrm{in}}^{y}$ and $H_{M}=H_{\mathrm{ext}}^{y}$, and we show that $\mathrm{SD}\left(\ell \left(H_{m}\right),\ell \left(H_{m-1}\right)\right)\leq \epsilon \left(n\right)$ for all $m\in \left[M\right]$ and $\ell \in LEAK^{\prime }$. We denote by $G_{o}$ ($G_{i}$) the gadget whose output (input) is the *m*’th bundle, and fix all wires in $H_{m},H_{m-1}$ except for: the *m*’th bundle; the masking inputs, outputs, and internal wires of $G_{o}$; the masking inputs, and internal wires, of $G_{i}$, as well as its input wire corresponding to the *m*’th bundle; the internal wires of all gadgets $G^{\prime }$ of ${\hat{C}}_{0-\mathrm{check}}$ connected to $G_{o}$ or $G_{i}$; and the internal wires of the decoding sub-circuits of $C_{\mathrm{dec}}$ whose inputs are masking inputs of one of these $G^{\prime }$’s.

Let $W_{R}\leftarrow {\mathrm{Enc}}^{\mathrm{in}}\left(b,1^{S\left(n\right)}\right)$ (where *b* is the value encoded by the *m*’th bundle in $H_{\mathrm{in}}^{y}$), and $W_{S}\leftarrow {\mathrm{Enc}}^{\mathrm{in}}\left(r,1^{S\left(n\right)}\right)$ for a random *r* (except if *m* corresponds to the output bundle, in which case $W_{S}\leftarrow {\mathrm{Enc}}^{\mathrm{in}}\left(1,1^{S\left(n\right)}\right)$). We define a distribution F over $SHALLOW\left(6,O\left({\hat{n}}^{4}\left(1,S\left(n\right)\right)\cdot S\left(n\right)\right)\right)$ as follows. Sampling a function f←F is performed by sampling ${\mathrm{rec}}_{o},{\mathrm{rec}}_{i}$ from the distribution over reconstructors for $G_{o},G_{i}$, respectively (see Definition 14), and sampling reconstructors ${\mathrm{rec}}_{G^{\prime }}$ for the gadgets of ${\hat{C}}_{0-\mathrm{check}}$ connected to $G_{o}$ or $G_{i}$. The function *f* has all the hard-wired values of $H_{m-1}$ hard-wired into it. On input $e\in F^{\hat{n}\left(1,Sn\right)}$, *f*: (1) evaluates ${\mathrm{rec}}_{o}$ on the (hard-wired) inputs of $G_{o}$, and the output e (this reconstructs the masking inputs, and internal wires, of $G_{o}$); (2) evaluates ${\mathrm{rec}}_{i}$ on e as one of the inputs, and the other (hard-wired) input and output of $G_{i}$; (3) for every gadget $G^{\prime }$ of ${\hat{C}}_{0-\mathrm{check}}$ connected to $G_{o}$ or $G_{i}$, uses ${\mathrm{rec}}_{G^{\prime }}$ to generate its internal wires; and finally (4) uses the functions of Lemma 7 to generate the internal wires of the decoding sub-circuits that decode the masking inputs used in the $G^{\prime }$’s. Then $f\in SHALLOW\left(6,O\left({\hat{n}}^{4}\left(1,S\left(n\right)\right)\cdot S\left(n\right)\right)\right)$ because the reconstructors ${\mathrm{rec}}_{o},{\mathrm{rec}}_{i}\in SHALLOW\left(2,O\left({\hat{n}}^{2}\left(1,S\left(n\right)\right)\right)\right)$ (by Lemma 4) and can be evaluated in parallel, the $O\left({\hat{n}}^{2}\left(1,S\left(n\right)\right)\cdot S\left(n\right)\right)$ reconstructors ${\mathrm{rec}}_{G^{\prime }}\in SHALLOW\left(2,O\left({\hat{n}}^{2}\left(1,S\left(n\right)\right)\right)\right)$ (by Lemma 7), and can be evaluated in parallel, and the $O\left({\hat{n}}^{3}\left(1,S\left(n\right)\right)\cdot S\left(n\right)\right)$ reconstructors of the decoding sub-circuits are each computable in $SHALLOW\left(2,O\left(\hat{n}\left(1,S\left(n\right)\right)\right)\right)$ (by Lemma 7) and can be evaluated in parallel.

Consequently, if $W_{R}^{\prime }:=f\left(W_{R}\right),W_{S}^{\prime }:=f\left(W_{S}\right)$ for f←F, then $W_{R}^{\prime }\equiv H_{m-1},W_{S}^{\prime }\equiv H_{m}$ and by Lemma 6, $W_{R}^{\prime },W_{S}^{\prime }$ are $\left(LEAK^{\prime },\epsilon \left(n\right)\right)$-leakage resilient for any $LEAK^{\prime }$ such that $LEAK^{\prime }\circ SHALLOW\left(6,O\left({\hat{n}}^{4}\left(1,S\left(n\right)\right)\cdot S\left(n\right)\right)\right)\subseteq LEAK_{E}$.

**Bounding $\mathrm{SD}\left(\ell \left(H_{\mathrm{ext}}^{y}\right),\ell \left(H_{\mathrm{ext},2}^{y,x}\right)\right)$:** it holds that $\mathrm{SD}\left(\ell \left(H_{\mathrm{ext}}^{y}\right),\ell \left(H_{\mathrm{ext},2}^{y,x}\right)\right)=0$ for every *ℓ* because the hybrids are independent of the input for ${\hat{C}}_{2}$ (since the input is re-sampled as a fresh $z\in_{R}F^{n}$ in both).

**Bounding $\mathrm{SD}\left(\ell \left(H_{\mathrm{ext},2}^{y,x}\right),\ell \left(H_{\mathrm{in},2}^{y,x}\right)\right)$:** We show that $\mathrm{SD}\left(\ell \left(H_{\mathrm{ext},2}^{y,x}\right),\ell \left(H_{\mathrm{in},2}^{y,x}\right)\right)\leq \epsilon \left(n\right)\cdot S\left(n\right)$ for all $\ell \in LEAK^{\prime }$ such that $LEAK^{\prime }\circ SHALLOW\left(6,O\left({\hat{n}}^{4}\left(1,S\left(n\right)\right)\cdot S\left(n\right)\right)\right)\subseteq LEAK_{E}$. The proof is similar to the proof that $\mathrm{SD}\left(\ell \left(H_{\mathrm{ext}}^{y}\right),\ell \left(H_{\mathrm{in}}^{y}\right)\right)\leq \epsilon \left(n\right)\cdot S\left(n\right)$, because the argument was independent of the actual inputs used in ${\hat{C}}_{1},{\hat{C}}_{2}$ (as long as both hybrids use the same input in each copy).

**Bounding $\mathrm{SD}\left(\ell \left(H_{\mathrm{in},2}^{y,x}\right),\ell \left(H^{y,x}\right)\right)$:** We show that $\mathrm{SD}\left(\ell \left(H_{\mathrm{in},2}^{y,x}\right),\ell \left(H^{y,x}\right)\right)\leq \epsilon \left(n\right)\cdot \hat{n}\left(1,S\left(n\right)\right)\cdot S\left(n\right)$ for all $\ell \in LEAK^{\prime }$ such that $LEAK^{\prime }\circ SHALLOW\left(7,O\left({\hat{n}}^{4}\left(1,S\left(n\right)\right)\cdot S\left(n\right)\right)\right)\subseteq LEAK_{E}$. The proof is similar to the proof that $\mathrm{SD}\left(\ell \left(H^{y}\right),\ell \left(H_{\mathrm{in}}^{y}\right)\right)\leq \epsilon \left(n\right)\cdot \hat{n}\left(1,S\left(n\right)\right)\cdot S\left(n\right)$, because the argument was independent of the actual inputs used in ${\hat{C}}_{1},{\hat{C}}_{2}$.

**Bounding $\mathrm{SD}\left(\ell \left(H^{x}\right),\ell \left(H^{y}\right)\right)$ for $\ell \in LEAK^{\prime }$.** From this analysis, we can now conclude using the union bound that for every $\ell \in LEAK^{\prime }$ such that

$$LEAK^{\prime }\circ SHALLOW\left(7,O\left({\hat{n}}^{5}\left(1,S\left(n\right)\right)\cdot S\left(n\right)\right)\right)\subseteq LEAK_{E}$$

it holds that

$$\mathrm{SD}\left(\ell \left(H^{x}\right),\ell \left(H^{y}\right)\right)\leq 4\epsilon \left(n\right)\cdot S\left(n\right)\cdot \left(\hat{n}\left(1,S\left(n\right)\right)+1\right).$$

### 4.3. An SAT-Respecting LRCC against “Useful” Leakage

Ishai et al. [8] use Construction 10 to devise an SAT-respecting LRCC which they later employ in their WI-PCP construction (described in Section 4.4). This is performed in two steps. First, since the LRCC will be used to compile verification circuits of NP relations, we need the compiler to be *Boolean*. Second, we need to instantiate the internal encoding scheme E such that it would resist leakage computable by functions that: (1) apply the PCP-prover algorithm, and then (2) restrict the output to a small subset of bits. Indeed, this is exactly the “leakage” on the witness which a query-bounded verifier (even a malicious one) obtains by querying the proof. We now provide more details on each of these steps.

**Step (1): An SAT-Respecting LRCC for*****Boolean*****Circuits.** The high-level idea is to transform the Boolean circuit $C:{\left\{0,1\right\}}^{n}\to \left\{0,1\right\}$ into a functionally-equivalent arithmetic circuit $C_{3}$ over $F_{3}$ (i.e., the field with 3 elements), use Construction 10 over the field $F_{3}$ to compile $C_{3}$ into its leakage-resilient version $\hat{C_{3}}$, and then output the Boolean circuit $\hat{C}$ that emulates $\hat{C_{3}}$ using Boolean operations. This is an over-simplified description of the compiler, where the actual construction needs to address several subtleties. We now describe each of these steps, and the subtleties that arise, in more detail.

**From Boolean to Arithmetic Operations.** The circuit $C_{3}$ is obtained from *C* by representing each Boolean operation using an appropriate polynomial over $F_{3}$ in the natural way. While $C_{3}$ is guaranteed to be functionally equivalent to *C* on binary strings, two issues arise concerning the SAT-respecting property. First, satisfiability over $F_{2}$ means that $C\left(x\right)=1$ for some *x*, whereas satisfiability over $F_{3}$ means $C_{3}\left(y\right)=0$ for some *y*. In particular, we want the leakage-resilient circuit to output 1 only if there exists an *x* such that $C\left(x\right)$ outputs 1, whereas the SAT-respecting property guarantees only that if $\hat{C_{3}}\left(\hat{y}\right)=0$ for some $\hat{y}$, then $C_{3}\left(y\right)=0$ for some *y*. Therefore, we need to “translate” a 1-output of *C* into a 0-output of $C_{3}$, and a 0-output of *C* into a non-0-output of $C_{3}$. Thus, we will have that $\hat{C_{3}}=\left(\hat{y}\right)=0$ for some $\hat{y}$ only if $C_{3}\left(y\right)=0$ for some *y*. This brings us to the second issue: while we would like to use the function-equivalence of *C* and $C_{3}$ to claim that if $C_{3}\left(y\right)=0$ for some $y\in F_{3}^{n}$ then $C\left(x\right)=1$ for some $x\in F_{2}^{n}$, this is not necessarily the case, because $C_{3}$’s inputs are from $F_{3}^{n}$ and might *not* correspond to an input in $F_{2}^{n}$. To overcome this issue, we add to $C_{3}$ an “input checker” component which checks that each of its *n* inputs is a bit. We denote this “enhanced” version of $C_{3}$—which flips the output and checks validity of the inputs—by $C_{3}^{\prime }$.

**From Arithmetic Back To Boolean.** Once we generate the leakage-resilient version $\hat{C_{3}^{\prime }}$ of $C_{3}^{\prime }$, we need to represent it using a *Boolean* circuit. (Indeed, the original circuit *C* was Boolean, and its leakage-resilient version should also be a Boolean circuit.) We do so by replacing each field element with a binary string representing it, and implementing each gate over $F_{3}$ by a Boolean sub-circuit. We also flip the output of $\hat{C_{3}^{\prime }}$, so the resultant circuit would again be functionally-equivalent to *C*. There are two important points that we need to handle. First, while we can represent field elements using any (injective) encoding scheme, to preserve the SAT-respecting property it must also be onto. Otherwise, the Boolean circuit could potentially be satisfied using invalid encodings, namely ones that do not encode any field element, and thus the computation in the Boolean circuit would not correspond to a computation in $\hat{C_{3}^{\prime }}$. In particular, the Boolean sub-circuits implementing gates over $F_{3}$ must be defined for all possible encodings—even ones that would not be used in an honest evaluation of the circuit.

Second, these Boolean sub-circuits should have small depth and size. The reason is the reduction from the leakage resilience of the final circuit $\hat{C}$ to the leakage resilience of $\hat{C_{3}^{\prime }}$. More specifically, the reduction proceeds by assuming that a leakage function *ℓ* in some class LEAK can distinguish between the wire values $\left[\hat{C},\hat{x}\right]$ of $\hat{C}$ on an encoding $\hat{x}$ of some input *x*, and its wire values $\left[\hat{C},\hat{x^{\prime }}\right]$ on the encoding $\hat{x^{\prime }}$ of some other input $x^{\prime }$ such that $C\left(x\right)=C\left(x^{\prime }\right)$. It then uses this to break the leakage resilience of $\hat{C_{3}^{\prime }}$ for some leakage function $\ell_{3}$ in the leakage class $LEAK_{3}$ against which the arithmetic LRCC is secure. This is performed as follows: given the wire values of $\hat{C_{3}^{\prime }}$ on some input (these values are elements of $F_{3}$), the reduction first replaces the field elements with the corresponding encodings. The resultant values constitute only *part* of the wire values of $\hat{C}$. Specifically, these are the values of the wires *between the sub-circuits emulating the gates over $F_{3}$*, whereas $\hat{C}$ contains also the internal wires of these sub-circuits, namely wires which do not appear in $\hat{C_{3}^{\prime }}$. Thus, the leakage function $\ell_{3}$ must first generate these missing wires, and only then can it evaluate *ℓ*. In particular, $LEAK\subset LEAK_{3}$, where the difference between the two classes depends on the complexity of the Boolean sub-circuits implementing gates over $F_{3}$. Fortunately, these sub-circuits are both shallow and small.

In summary, Ishai et al. show [47] the following Boolean LRCC.

**Claim** **13** (Boolean SAT-respecting LRCC). *Let $LEAK,LEAK_{E}$ be families of functions, $S\left(n\right):N\to N$ be a size function, and $\epsilon \left(n\right):N\to R^{+}$. Let $E^{\mathrm{in}}=\left({\mathrm{Enc}}^{\mathrm{in}},{\mathrm{Dec}}^{\mathrm{in}}\right)$ be a linear, onto, $\left(LEAK_{E},\epsilon \left(n\right)\right)$-leakage-resilient encoding scheme with parameters n, σ and $\hat{n}=\hat{n}\left(n,\sigma \right)$, such that $LEAK_{E}=LEAK\circ BOOL\left(33,O\left({\hat{n}}^{6}\left(1,S\left(n\right)\right)\cdot S^{2}\left(n\right)\right)\right)$. Then there exist constants $c,c^{\prime }>0$ for which there exists an SAT-respecting, $\left(LEAK,c^{\prime }\cdot \epsilon \left(n\right)\cdot \left(\hat{n}\left(1,c\cdot S\left(n\right)\right)+1\right)\cdot c\cdot S\left(n\right),S\left(n\right)\right)$-LRCC over {0,1}. Moreover, for every $C:{\left\{0,1\right\}}^{n}\to \left\{0,1\right\}$, the compiled circuit $\hat{C}$ has size $\left|\hat{C}\right|=O\left({\hat{n}}^{6}\left(1,c\cdot S\left(n\right)\right)\cdot {\left|C\right|}^{2}\right)$.*

**Step (2): Leakage Resilience Against “Useful” Leakage.** The second component of the construction is an internal encoding scheme $E_{\mathrm{in}}$—resisting leakage from a “useful” class of leakage functions—with which we instantiate Claim 13. More specifically, the leakage class consists of $AC^{0}$ circuits (namely, constant-depth, polynomial-sized Boolean circuits over unbounded fan-in and fan-out ∧,∨,¬ gates), *augmented with a sublinear number of ⊕ gates of unbounded fan-in and fan-out*. Formally,

**Notation** **10** ($L_{n,d,s,⊕t}^{m}$ leakage family). *Let n,d,s∈N be length, depth and size parameters (respectively), and let t∈N be a parity gate bound. The family $L_{n,d,s,⊕t}$ consists of all functions computable by a Boolean circuit $C:{\left\{0,1\right\}}^{n}\to \left\{0,1\right\}$ of size at most s and depth d, with unbounded fan-in and fan-out ∧,∨,¬,⊕ gates, out of which at most t are ⊕ gates. We denote $L_{d,s,⊕t}=\cup_{n\in N}L_{n,d,s,⊕t}$.*

*For a length parameter m∈N, and a function $f:{\left\{0,1\right\}}^{n}\to {\left\{0,1\right\}}^{m}$, let $f_{i}\left(x_{1},\ldots ,x_{n}\right),i\in \left[m\right]$ denote the i’th output bit of f. We denote: $L_{n,d,s,⊕t}^{m}=\left\{f:{\left\{0,1\right\}}^{n}\to {\left\{0,1\right\}}^{m}:\forall 1\leq i\leq m,f_{i}\in L_{n,d,s,⊕t}\right\}$, and $L_{d,s,⊕t}^{m}:=\cup_{n\in N}\left(L_{n,d,s,⊕t}^{m}\right)$.*

The encodings scheme we use encodes elements $\gamma \in F_{3}$ as binary strings whose sum mod 3 is γ. Formally:

**Notation** **11.** *For γ∈{0,1,2} and n∈N, $U_{\gamma }^{n}$ denotes the uniform distribution over $\left\{v\in {\left\{0,1\right\}}^{3n}:\;{\#}_{1}\left(v\right)\equiv \gamma \;\mathrm{mod}\;3\right\}$, where ${\#}_{1}\left(v\right)$ denotes the number of 1’s in v.*

**Definition** **16.** *The encodings scheme $E_{3}=\left({\mathrm{Enc}}_{3},{\mathrm{Dec}}_{3}\right)$ is defined as follows. For every $\gamma \in F_{3}$, ${\mathrm{Enc}}_{3}\left(\gamma ,1^{n}\right)$ samples from $U_{\gamma }^{n}$, and ${\mathrm{Dec}}_{3}\left(v\right)$ returns $\left({\#}_{1}\left(v\right)\;\mathrm{mod}\;3\right)$. We note that $E_{3}$ is linear and onto.*

**Remark** **14.** *${\mathrm{Enc}}_{3}$ can be computed efficiently by repeating the following procedure $n^{2}$ times. Pick $v\in {\left\{0,1\right\}}^{3n}$ uniformly at random, compute $t:={\#}_{1}\left(v\right)$, and if t=γ then return v. If all iterations fail, return a fixed $v_{\gamma }\in {\left\{0,1\right\}}^{3n}$ such that ${\#}_{1}\left(v_{\gamma }\right)=\gamma$. Then the output of ${\mathrm{Enc}}_{3}$ is thus statistically close to $U_{\gamma }^{n}$.*

Ishai et al. [8] show that the encoding scheme of Definition 16 resists leakage from $AC^{0}$ circuits augmented with few ⊕ gates:

**Corollary** **3** (Corollary 3.44 in [47]). *For every constant depth parameter d∈N there exist constants $c,\epsilon \in \left(0,1\right)$, such that for every constant l∈N there exists a minimal length parameter $n_{0}\in N$ such that for every $n\geq n_{0}$ the encoding scheme ${\mathrm{Enc}}_{3}\left(\cdot ,1^{n}\right)$ of Definition 16 is $\left(L_{3n,d,n^{l},⊕n^{\epsilon }},2^{-n^{c}}\right)$-leakage resilient.*

Instantiating Claim 13 with the encoding scheme of Definition 16 as the internal encoding scheme, and using Corollary 3, ref. [8] show the existence of a Boolean LRCC resisting leakage from $AC^{0}$ circuits with few ⊕ gates:

**Theorem** **15** (Boolean SAT-respecting LRCC for $AC^{0}$ circuits with ⊕ gates, Theorem 3.37 in [47]). *Let n∈N be an input length parameter. For every positive constant d,c, polynomials $m=m\left(n\right),t=\left(n\right)$, and polynomial size bound $s=s\left(n\right)$, there exists a polynomial $l\left(n\right)$, such that the following holds. There exists an SAT-respecting $\left(L_{l,d,l^{c},⊕t}^{m},2^{-n^{c}},s\left(n\right)\right)$-LRCC over {0,1}, which on input a circuit $C:{\left\{0,1\right\}}^{n}\to \left\{0,1\right\}$ of size $\left|C\right|\leq s\left(n\right)$ outputs a circuit $\hat{C}$ of size $\left|\hat{C}\right|\leq l\left(n\right)$.*

### 4.4. The Witness-Indistinguishable PCP

In this section, we describe the WI-PCPs for NP of [8], which rely on the Boolean SAT-respecting LRCC of Theorem 15. They use also a PCP system $\left(P^{\prime },V^{\prime }\right)$ for the language 3SAT of all satisfiable 3CNF formulas, in which the prover algorithm can be implemented in a low complexity class.

**Figure 3:**
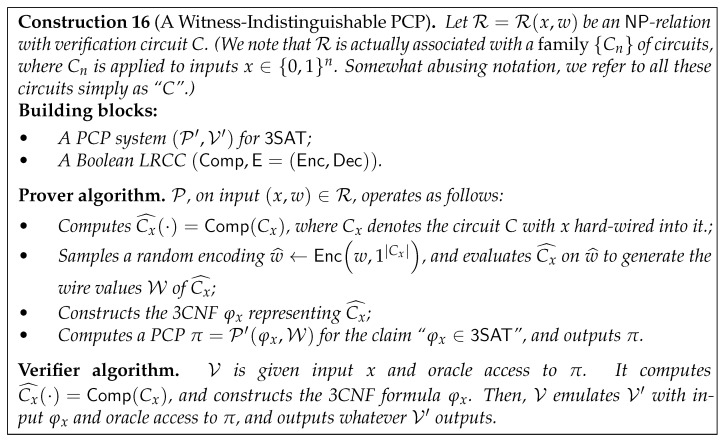
Witness-Indistinguishable PCPs from SAT-Respecting LRCCs [8].

The high-level idea of the construction for an NP-relation R with verification circuit *C* is that given input *x*, instead of verifying that $C_{x}\left(\cdot \right):=C\left(x,\cdot \right)$ is satisfiable (which holds if and only if x∈L for the corresponding NP-language L), the verifier will check that the *leakage-resilient version*$\hat{C_{x}}$ is satisfiable. For this, the prover and verifier will first represent $\hat{C_{x}}$ as a 3CNF formula φ in the natural way. That is, φ will have a variable for each wire of $\hat{C_{x}}$. It will contain, for each gate *g* of $\hat{C_{x}}$, a sub-formula verifying that the output wire of *g* is consistent with the input wires and the operation of *g*, and it will also check that the output wire of $\hat{C_{x}}$ is 1. A satisfying assignment for φ is the wire values of $\hat{C_{x}}$ when evaluated on (an encoding of) a witness *w* for *x*. Then, the prover and verifier will use the PCP system $\left(P^{\prime },V^{\prime }\right)$ to verify that φ∈3SAT. The construction is described in Figure 3.

The following theorem (which is a combination of ([47], Proposition 4.4) and ([47], Corollary 4.10)) asserts the connection between the properties of the LRCC and the resultant PCP system $\left(P,V\right)$.

**Theorem** **17.** *Let n∈N be a length parameter, $q^{*}=q^{*}\left(n\right),S=S\left(n\right)$ be query and size functions, $\epsilon ,\epsilon^{\prime }\in \left[0,1\right]$, and LEAK be a family of leakage functions. Assume that Construction 16 is instantiated with:*

- *A Boolean SAT-respecting $\left(LEAK,\epsilon ,S\right)$-LRCC such that there exists a polynomial $g\left(\cdot \right)$ for which $\left|\mathrm{Comp}\left(C\right)\right|\leq g\left(\left|C\right|\right)$ for every circuit C; and*
- *A PCP system $\left(P^{\prime },V^{\prime }\right)$ for 3SAT with proofs of length $\mathrm{len}\left(n\right)$, such that for every $\left(\varphi ,W\right)\in 3\mathrm{SAT}$, every subset Q of $q^{*}$ bits of an honestly-generated proof $\pi =\pi \left(\varphi ,W\right)$ is computable from W by a function $f_{\varphi ,Q}\in LEAK$.*

*Then for every NP-relation R with verification circuit C of size $\left|C\right|\leq S$, the PCP system $\left(P,V\right)$ is a $\left(q^{*},\epsilon^{*}\right)$-WI-PCP for R, where $\epsilon^{*}=O\left(\epsilon \cdot q^{*}\cdot {\mathrm{len}}^{2q^{*}}\left(t\right)\right)+e^{-\Omega \left(q^{*}\cdot {\mathrm{len}}^{q^{*}}\left(t\right)\right)}$ and $t=O\left(g\left(\left|C\right|\right)\right)$. Moreover, if $V^{\prime }$ is non-adaptive, the so is V.*

*Furthermore, the system is $\left(q^{*},\epsilon \right)$-WI against* non-adaptive *(possibly malicious) verifiers. Moreover, proofs generated by P have length $\mathrm{len}\left(t\right)$, and if $V^{\prime }$ has query complexity $q\left(n\right)$ and tosses $r\left(n\right)$ coins, then V has query complexity $q\left(t\right)$, tosses $r\left(t\right)$ coins.*

**Proof.**  We first analyze the parameters of the system. The wire assignment W to $\hat{C_{x}}$ has size $\left|W\right|=\left|\hat{C_{x}}\right|\leq g\left(\left|C_{x}\right|\right)\leq g\left(\left|C\right|\right)$, and $\left|\varphi_{x}\right|=O\left(\left|\hat{C_{x}}\right|\right)\leq O\left(g\left(\left|C\right|\right)\right)$. Therefore, the internal PCP system $\left(P^{\prime },V^{\prime }\right)$ is emulated using inputs of size $t=g\left(\left|C\right|\right)$.

**Completeness** follows directly from the completeness of the building blocks.

**Soundness** follows from a combination of the soundness of $\left(P^{\prime },V^{\prime }\right)$ and the SAT-respecting property of $\left(\mathrm{Comp},E\right)$, Indeed, if x∉L, then $C_{x}$ is not satisfiable, and so (by the SAT-respecting property) $\hat{C_{x}}$ is not satisfiable, i.e., $\varphi_{x}\notin 3\mathrm{SAT}$. Therefore, by the soundness of $\left(P^{\prime },V^{\prime }\right)$ we have that $\mathrm{Pr}\left[V^{\pi^{*}}\left(x\right)=1\right]=\mathrm{Pr}\left[V^{\prime \pi^{*}}\left(\varphi_{x}\right)=1\right]$ is negligible.

**Witness-indistinguishability.** Let x∈L, $\varphi_{x}$ be the 3CNF formula representing $\hat{C_{x}}$, and $w_{1},w_{2}$ be two witnesses for *x*. We first show witness indistinguishability against *non-adaptive* verifiers. Let $V^{*}$ be a non-adaptive $q^{*}$-query-bounded verifier, and let $\pi_{i}\leftarrow P\left(x,w_{i}\right)$ for i=1,2. Since $V^{*}$’s entire view can be generated from the oracle answers to its queries (and this cannot increase the statistical distance), it suffices to show that $\mathrm{SD}\left(\pi_{1}{{|}_{Q},\pi_{2}|}_{Q}\right)\leq \epsilon$ for every set Q of queries of $V^{*}$ such that size $\left|Q\right|\leq q^{*}$, where $\pi_{i}{|}_{Q}$ denotes the restriction of $\pi_{i}$ to the entries in Q. Since $\left|C\right|=\left|C_{x}\right|\leq S$, the leakage resilience of the LRCC guarantees that $\mathrm{SD}\left(\ell \left[\hat{C_{x}},{\hat{w}}_{1}\right],\ell \left[\hat{C_{x}},{\hat{w}}_{2}\right]\right)\leq \epsilon$ for every ℓ∈LEAK. We conclude the proof by noting that $f_{\varphi_{x},Q}\in LEAK$, and $\pi_{i}{|}_{Q}=f_{\varphi_{x},Q}\left[\hat{C_{x}},\hat{w_{i}}\right]$. We have shown that $\left(P,V\right)$ is $\left(q^{*},\epsilon \right)$-witness indistinguishable against *non-adaptive* verifiers $V^{*}$. Using Theorem 18 below, this implies that $\left(P,V\right)$ is $\left(q^{*},\epsilon^{*}\right)$-WI (even against *adaptive* verifiers), for $\epsilon^{*}=O\left(\epsilon \cdot q^{*}\cdot {\mathrm{len}}^{2q^{*}}\left(t\right)\right)+e^{-\Omega \left(q^{*}\cdot {\mathrm{len}}^{q^{*}}\left(t\right)\right)}$. □

The proof of Theorem 17 used the following theorem, which is implicit in [48] (see also [47], Theorem 4.11).

**Theorem** **18** (Implicit in [48]). *Let $\left(P,V\right)$ be a PCP system that is $\left(q^{*},\epsilon \right)$-WI against* non-adaptive *verifiers, with proofs of length len. Then $\left(P,V\right)$ is $\left(q^{*},\epsilon^{*}\right)$-WI against* adaptive *verifiers, where $\epsilon^{*}=O\left(\epsilon \cdot q^{*}\cdot {\mathrm{len}}^{2q^{*}}\right)+e^{-\Omega \left(q^{*}\cdot {\mathrm{len}}^{q^{*}}\right)}$.*

Theorem 7 now follows as a corollary of Theorem 17, using the SAT-respecting LRCC of Theorem 15, and a PCP system of [2], whose prover algorithm can be implemented in a low complexity class (the analysis of the prover complexity is due to ([47], Appendix B)):

**Theorem** **19** (PCPs for NP, [2]). *3SAT has a PCP system $\left(P,V\right)$ with soundness error 1/2 with an honest verifier that queries $O\left(\log^{2}n\right)$ proof bits. The proofs have length $\mathrm{poly}\left(n\right)$, where every proof bit can be generated by an $AC^{0}$ circuit with a single ⊕ gate of unbounded fan-in.*

We are now ready to prove Theorem 7.

**Proof of Theorem 7**.  We instantiate Construction 16 with the PCP system $\left(P^{\prime },V^{\prime }\right)$ of Theorem 19 and the LRCC of Theorem 15. By Theorem 19, there exist constants d,c∈N such that every bit in a proof generated by $P^{\prime }$ is computable from the NP-witness in $L_{d,n^{c},⊕1}$, where *n* is the witness length, and the proofs have length $n^{c^{\prime \prime }}$, for some constant $c^{\prime \prime }$.

Let R be an NP-relation with verification circuit *C*, then $\left|C\right|=n^{c^{\prime }}$ for some constant $c^{\prime }$. We instantiate Theorem 15 with parameters $d^{*}=d,s^{*}=\left|C\right|,n^{*}=n$, $t^{*}=1$, $m^{*}=q^{*}$, and $c^{*}\geq c$ which is a sufficiently large constant whose value is set below. Here, the superscript ${}^{*}$ is used to denote the parameters of Theorem 15, and $s^{*},t^{*},m^{*}$ are in $\mathrm{poly}\left(n\right)$. Let $\left(\mathrm{Comp},E\right)$ denote the LRCC obtained from Theorem 15. We compute $\hat{C}=\mathrm{Comp}\left(C\right)$, where $\left|\hat{C}\right|\leq l\left(n\right)$ which, because $\left|C\right|\leq s^{*}$, is $\left(L_{d,l^{c}\left(n\right),⊕1}^{q^{*}},2^{-n^{c^{*}}}\right)$-LR (where $l\left(n\right)$ is the polynomial whose existence is guaranteed by Theorem 15).

Let φ denote the 3CNF representing $\hat{C}$. Then by Theorem 19 (and using the fact that $\left|W\right|=\left|\hat{C}\right|\leq l\left(n\right)$), every bit of a proof generated by $P^{\prime }$ for φ can be generated from a wire assignment W of $\hat{C}$ in $L_{d,l^{c}\left(n\right),⊕1}$, so every $q^{*}$ proof bits are computable from W in $L_{d,l^{c}\left(n\right),⊕1}^{q^{*}}$. Therefore, Theorem 17 guarantees that the system $\left(P,V\right)$ of Construction 16 is a non-adaptive WI-PCP system for R, with $\left(q^{*},O\left(2^{-n^{c^{*}}}\cdot q^{*}\cdot {\mathrm{len}}^{2q^{*}}\left(l\left(n\right)\right)\right)+e^{-\Omega \left(q^{*}\cdot {\mathrm{len}}^{q^{*}}\left(l\left(n\right)\right)\right)}\right)$-WI (where $\mathrm{len}\left(l\left(n\right)\right)$ denotes the proof length), and soundness error 1/2 with an honest verifier that queries $O\left(\log^{2}\left(l\left(n\right)\right)\right)=\mathrm{polylog}\left(n\right)\leq \mathrm{polylog}\left(q^{*}\right)$ proof bits. We set $c^{*}$ to be sufficiently large, such that the statistical LR error satisfies

$$O\left(2^{-n^{c^{*}}}\cdot q^{*}\cdot {\mathrm{len}}^{2q^{*}}\left(l\left(n\right)\right)\right)+e^{-\Omega \left(q^{*}\cdot {\mathrm{len}}^{q^{*}}\left(l\left(n\right)\right)\right)}=\mathrm{negl}\left(q^{*}\right)\leq \mathrm{negl}\left(\kappa \right).$$

(We note that such a constant exists since we assume that $q^{*}=\mathrm{poly}\left(n\right)$.) We conclude the proof by noting that soundness can be amplified to $\mathrm{negl}\left(\kappa \right)$ with only a $\mathrm{poly}\left(\kappa \right)$ blowup in the query complexity of the honest verifier. □

## 5. Discussion

The works of [8,13] show a connection between ZK-PCPs and the seemingly-unrelated field of leakage-resilient cryptography, and use it to circumvent an inherent limitation of previous constructions—that the *honest* verifier is adaptive. Specifically, using tools from the leakage-resilience literature, [8,13] put forth two new paradigms of constructing ZK-PCPs, yielding PCPs with ZK against malicious verifiers, in which the honest verifier is *non-adaptive*. In the context of cryptographic applications of ZK-PCPs, non-adaptive verification translates into fewer communication rounds. The paradigm of [13] also extends to ZK-PCPs *of proximity*.

Despite this recent progress, several interesting questions remain open. The obvious open problem is to obtain ZK-PCPs and ZK-PCPPs with an exponential query gap as in [6,11] but which can be verified non-adaptively. One possible approach is to design a ZK-PCP variant over a large alphabet with negligible soundness error and an honest verifier that makes fewer queries than [15] (hopefully, polylogarithmic). Another interesting research direction is to extend the techniques of [8,13] to other related proof systems, such as interactive oracle proofs. Finding further applications of ZK-PCPs and ZK-PCPPs is also an interesting question. Finally, though in this survey we have focused on other parameters of ZK-PCPs, reducing the proof length is a fascinating open problem worthy of study. Whereas the locking-scheme-based ZK-PCPs of [6,11] inherently incur a polynomial blowup in proof length, another advantage of the leakage-resilience-based approach is that it opens up the possibility of reducing the proof length of ZK-PCPs, potentially even matching the proof length of non-ZK PCPs.

## Figures and Tables

**Table 1:** Comparison of Existing ZK-PCP constructions. Here, “Underlying LR primitive” refers to the type of building block used in the leakage-resilience based constructions; “WI” stands for witness-indistinguishable; “ZK quality” refers to the efficiency of the ZK simulator (which is efficient in ZK systems and inefficient in WI systems); “Query ratio” is $q^{*}/q$, where $q^{*}$ is the bound on the query complexity of a malicious verifier (ZK holds against any verifier querying at most $q^{*}$ proof bits), and *q* is the query complexity of the honest verifier (needed to achieve soundness); “Underlying PCP” describes the properties which the transformations needs the underlying PCP system to have; and “Honest verification” refers to the adaptivity of the honest verifier, where “NA” stands for non-adaptive.

	Underlying LR Primitive	ZK Quality	Query Ratio	Underlying PCP	Honest Verification
[8]	Circuits	WI	Exponential	Any standard PCP	Nonadaptive
[13]	Encodings	ZK	Square-root	ZK-PCP, large alphabet	Nonadaptive
[6,11]	—	ZK	Exponential	Any standard PCP	Adaptive

**Table 2:** Comparison Between Different Probabilistic Proof Systems. IOPPs are IOPs *of Proximity*, considered in, e.g., [28], in which the verifier has full access to the input, and oracle access to the witness. Here, “P↔V Communication” refers to whether there is direct communication between the prover and verifier (in particular, whether the verifier can send messages to the prover); “Access to Prover Messages” states whether the verifier reads prover messages in full, or has oracle access to them; similarly, “Access to Input” indicates whether the verifier reads the input in full, or only has oracle access to it; ”Soundness Guarantee” refers to the type of inputs which are guaranteed to be rejected, where ”Full” means that all inputs not in the languages are rejected, whereas ”Promise (input)” means that only inputs that are *far* (in relative Hamming distance) from the language are guaranteed to be rejected, and “Promise (witness)” only guarantees that V rejects when given oracle access to a witness which is far (in relative Hamming distance) from all valid witnesses for the input; “ZK Variant Hides” of a system X states, for the ZK variant ZK-X of system X, which input of the prover remains hidden from the verifier, where ”Witness” means verification reveals no information about the underlying NP witness, and ”Witness, input (partial)” (resp. “Witness (partial)”) roughly means that a verifier making *q* queries to the input and proof(s) (resp. witness) learns only *q* physical bits of the input (resp., witness).

	P↔V Communication	Access to Prover Messages	# Prover Messages	Access to Input	Soundness Guarantee	ZK Variant Hides
IP	Yes	Full	Multiple	Full	Full	Witness
PCP	No	Oracle	Single	Full	Full	Witness
IOP	Yes	Oracle	Multiple	Full	Full	Witness
PCPP	No	Oracle	Single	Oracle	Promise (input)	Witness, input (partial)
IOPP	Yes	Oracle	Multiple	Full	Promise (witness)	Witness (partial)

## Data Availability

Not applicable.
